# Indicators Associated With Job Morale Among Physicians and Dentists in Low-Income and Middle-Income Countries: A Systematic Review and Meta-analysis

**DOI:** 10.1001/jamanetworkopen.2019.13202

**Published:** 2020-01-10

**Authors:** Alina Sabitova, Rose McGranahan, Francesco Altamore, Nikolina Jovanovic, Emma Windle, Stefan Priebe

**Affiliations:** 1 Unit for Social and Community Psychiatry, World Health Organization Collaborating Centre for Mental Health Development, Queen Mary University of London, London, United Kingdom; 2 Department of Public Health, Astana Medical University, Nur-Sultan, Kazakhstan; 3 Department of Biomedical, Metabolic, and Neuronal Sciences, University of Modena and Reggio Emilia, Modena, Italy

## Abstract

**Importance:**

Improving health care in low- and middle-income countries (LMICs) requires a workforce with positive job morale. However, the level of job morale in this population remains unclear.

**Objective:**

To analyze studies measuring the job morale of physicians and dentists working in LMICs, using levels of job burnout, job satisfaction, and job motivation as indicators of job morale.

**Data Sources:**

A comprehensive search of Scopus, PubMed, PsycINFO, EMBASE, Web of Science, and the Cochrane Library, from database inception to October 30, 2018, and gray literature was performed.

**Study Selection:**

Studies were eligible if at least 50% of the sample were qualified physicians and/or dentists working in public health care settings in LMICs. Three indicators of job morale in this population were used: job burnout, job satisfaction, and job motivation. Of 12 324 records reviewed, 79 studies were included in the systematic review and 59 were eligible for the meta-analysis.

**Data Extraction And Synthesis:**

Data were extracted independently by several investigators in accordance with the Meta-analyses of Observational Studies in Epidemiology (MOOSE) reporting guidelines. Random-effects meta-analyses, planned subgroup analyses, and metaregression were performed. The Quality Assessment Tool for Observational Cohort and Cross-Sectional Studies was used to assess bias.

**Main Outcomes and Measures:**

Levels of job burnout, job satisfaction, and job motivation as indicators of job morale.

**Results:**

Overall, 12 324 records were identified. Of them, 80 records, representing 79 studies and involving 45 714 participants across 37 LMICs, met the inclusion criteria for the review; however, only 3 were from low-income countries. In 21 studies with 9092 participants working mainly in middle-income countries, 32% (95% CI, 27%-38%; *I*^2^ = 95.32%; *P* < .001) reported job burnout, and in 20 studies with 14 113 participants, 60% (95% CI, 53%-67%; *I*^2^ = 98.21%; *P* < .001) were satisfied with their job overall. Sufficient data were not available for a meta-analysis of job motivation.

**Conclusions and Relevance:**

The findings suggest that, among physicians and dentists working mainly in middle-income countries, more than half reported having positive job morale. Positive job morale among physicians and dentists may help with recruitment and retention and thus support programs for improving health care in LMICs.

## Key Points

**Question:**

What are the levels of job burnout, job satisfaction, and job motivation, as indicators of job morale, among physicians and dentists working in low- and middle-income countries?

**Findings:**

This systematic review and meta-analysis, including results from 79 studies with 45 714 participants, found that 32% of physicians and dentists working mainly in middle-income countries exceeded the high threshold for job burnout and that 60% were satisfied with their job overall.

**Meaning:**

Despite high workloads, poor working conditions, and low salaries, more than half of the physicians and dentists working mainly in middle-income countries reported positive job morale.

## Teaser

This systematic review and meta-analysis analyzes studies that measure job morale, indicated by levels of job burnout, satisfaction, and motivation, among physicians and dentists working in low- and middle-income countries.

## Introduction

Job morale is a complex phenomenon and currently has no universally agreed-on definition.^1,2^ In a broad sense, it is undecided whether it is a group^3,4^ or individual^2,5,6^ concept or whether it is generalizable or context dependent.^3,7,8,9,10^ There is also little agreement about how job morale should be measured.^1,2^ Despite this ongoing debate, much of the literature refers to the importance of job satisfaction, job motivation, and job burnout in the assessment of job morale. Therefore, these 3 factors can be seen as indicators of job morale^11^ and are often measured using standardized methods. By measuring these indicators, positive job morale of health care professionals has been found to be an important factor for the provision of high-quality health care. It has been associated with better recruitment and retention of medical staff^12^ as well as better quality of care.^13,14,15,16^ A number of studies have found that better job-related well-being among health care staff is associated with higher patient satisfaction and better patient experiences of care.^13,16,17^ The World Health Organization suggests that higher job morale can contribute positively to 3 domains, ie, the accessibility, safety, and acceptability of care.^18^ In low- and middle-income counties (LMICs), significant shortages, maldistribution, and absenteeism of health care staff have a negative impact on services and could potentially lower the morale of staff working within services.^19,20,21^ If interventions were targeted to improve job morale, it could be possible to reduce spending on staff turnover and absences from illness.^20^ Improved job morale could also address issues of inadequate job performance in settings with scarce resources.^22^ Finally, qualified physicians and dentists have received the most extensive and expensive training of health care professionals and are at the center of health care provision. However, data suggest that many qualified physicians are moving away from LMICs to work in high-income countries (HICs).^23^ Job morale may be a factor in this move and therefore must be explored to enable LMICs to retain qualified physicians and dentists.

While various studies and reviews on indicators of job morale in health care professionals have included data from LMICs, previous reviews have not drawn together results from multiple LMICs while addressing the multifaceted nature of job morale.^2,12,24,25,26,27^ Previous reviews also included all health care staff in LMICs^9,25,28,29^ and did not distinguish between staff who have completed training and those who are still in training, even though job morale levels are associated with professional group and training status.^9,16,30,31,32^ In previous research, job burnout, job satisfaction, and job motivation levels were different depending on whether the service was private or public^33,34,35^; therefore, the setting must also be considered. The public sector is more relevant to the public health perspective and therefore requires focus from researchers. Against this background, we conducted a systematic review and meta-analysis addressing the following question: what are the levels of 3 indicators of job morale (ie, job burnout, job satisfaction, and job motivation) among physicians and dentists working in LMICs?

## Methods

The protocol for this review was registered on PROSPERO (CRD42017079713) in advance. This study followed the Meta-analysis of Observational Studies in Epidemiology (MOOSE) reporting guideline.^36^

### Search Strategy

The following 6 databases were searched by the first reviewer (A.S.) on January 31, 2018, and updated on October 30, 2018, with no date limits applied: Scopus, PubMed, PsycINFO, EMBASE, Web of Science, and the Cochrane Library. Search terms were developed by information scientists and combined 3 overlapping areas with key words such as *morale *OR *job motivation* OR *job satisfaction* OR *burnout* AND *physicians* OR *dentists* AND *LMICs* (eAppendix 1 in the Supplement). Publication bias was reduced by searching conference records and unpublished literature, using Google Scholar, OpenGrey, EThOS, the British Library Catalogue, and Copac theses. Additionally, backward and forward citation tracking was used for included studies and review records, and hand searches were performed in the following journals: *Human Resources for Health* and *The BMJ*, along with the journal indexes from 2003 to 2018 and from 1994 to 2018, respectively.

### Study Selection

Studies were eligible if they met the 3 following conditions: (1) used quantitative methods to assess at least 1 job morale indicator, ie, burnout, job satisfaction, or job motivation; (2) at least 50% of the sample was qualified physicians and/or dentists from LMICs, as defined by World Bank criteria^37^; and (3) if participant qualifications were not defined in the study, at least 50% of the sample had more years of experience than the maximum length of medical residency in the country of interest, defined by Wijnen-Meijer et al.^38^ Records were excluded if they met any of the 4 following criteria: (1) 50% or more of the sample was undertaking training at the time of the study (ie, medical students, residents, trainees, registrars, or junior physicians); (2) 50% or more of the sample was employed in private health care settings; (3) neither qualifications nor years of experience were reported; or (4) articles were only available in languages other than Latin script, Russian, or Kazakh.

### Identification and Data Extraction

The titles and abstracts of identified records were exported to EndNote X8 (Clarivate Analytics) and screened by 1 of us (A.S.) to exclude irrelevant studies and duplicates. A random subsample of 20% of titles and abstracts was screened by 1 of us (R.M.) to ensure accuracy of selection. Full-text articles were inspected by 3 of us (A.S., R.M., and F.A.) for relevance according to the inclusion criteria.

Data from included studies were extracted into a spreadsheet by 1 of us (A.S.), and 40% of the data were reviewed by 2 of us (R.M. and F.A.). Discrepancies were addressed by involving a fourth author (S.P.). The level of agreement between A.S. and R.M. was 85% and between A.S. and F.A., 80%. Articles written in languages other than English and Russian (ie, Spanish,^39,40,41,42,43,44,45,46,47^ Portuguese,^48,49^ French,^50^ and Turkish^51^) were extracted by native speakers. In articles with mixed samples, only data focusing on the sample of interest were extracted if possible.^52,53,54,55^ When data presented were unclear, study authors were contacted by 2 of us (A.S. and F.A.). In 5 instances, clarification was obtained from authors via email.^56,57,58,59,60^ A random subsample of 20% of the results used for meta-analysis were checked by another of us (E.W.).

### Quality Assessment

Risk of bias of the included records was assessed using the 14-item Quality Assessment Tool for Observational Cohort and Cross-Sectional Studies^61^ in accordance with 9 criteria, as 5 were not applicable. One of us (A.S.) completed full quality assessment and ensured accuracy by independently assessing 20% of records.

### Data Synthesis and Statistical Analysis

All meta-analyses were conducted in Stata statistical software version 15.1 (StataCorp). All meta-analysis commands are summarized in eAppendix 1 in the Supplement.

Dichotomous and continuous data were analyzed separately. The meta-analysis of dichotomous data aimed to determine the prevalence of burnout dimensions (ie, emotional exhaustion, depersonalization, and personal accomplishment) and the pooled proportion of satisfied physicians and dentists among physicians and dentists in LMICs. Prevalence rates for each indicator were defined from raw proportions reported in the included studies using the metaprop command.^62^ The Freeman-Tukey double arcsine transformation was used to stabilize the study-specific variances and avoid the squeezing of variance effect.^62,63^

The meta-analysis of continuous data aimed to define the pooled mean values for burnout and job satisfaction. Mean and SD values for each indicator were extracted from the included studies. The metan command^64^ for means and SEs was used with a calculation of SEs in advance.

Variances of raw proportions or means were pooled in a random-effects model.^65^ Heterogeneity between studies was estimated using the *I*^2^ test. The issue of publication bias was addressed by examining funnel plots^66,67,68^ and performing Egger tests.^69^

Sources of heterogeneity were examined by exploratory subgroup analyses for meta-analyses with at least 10 studies included^65,70^ using the following covariates: country income group, according to the World Bank classification^37^; physician specialty; geographical region, according to the United Nations classification^71^; and thresholds used (for burnout reported as dichotomous data only). Because subgroup analysis does not explain residual heterogeneity and targets the effects in each subgroup individually, univariate random-effect metaregression analysis was performed in studies that showed a difference in a subgroup analysis by using a metareg command.^72^ The metaregression aimed to explore whether differences in pooled estimates remained statistically significant among covariates, which showed differences in subgroup analyses and contained more than 10 studies per covariate.^65,73^ Sensitivity analyses^74^ were used to assess whether the robustness and stability of the meta-analyses^75^ were influenced by the exclusion of studies that were more susceptible to risk of bias (ie, where 5 of 9 risk-of-bias criteria were rated unclear or no), of studies in which respondents were not only qualified physicians or dentists, and of studies in which the health care setting was not reported.

## Results

**Figure 1.**
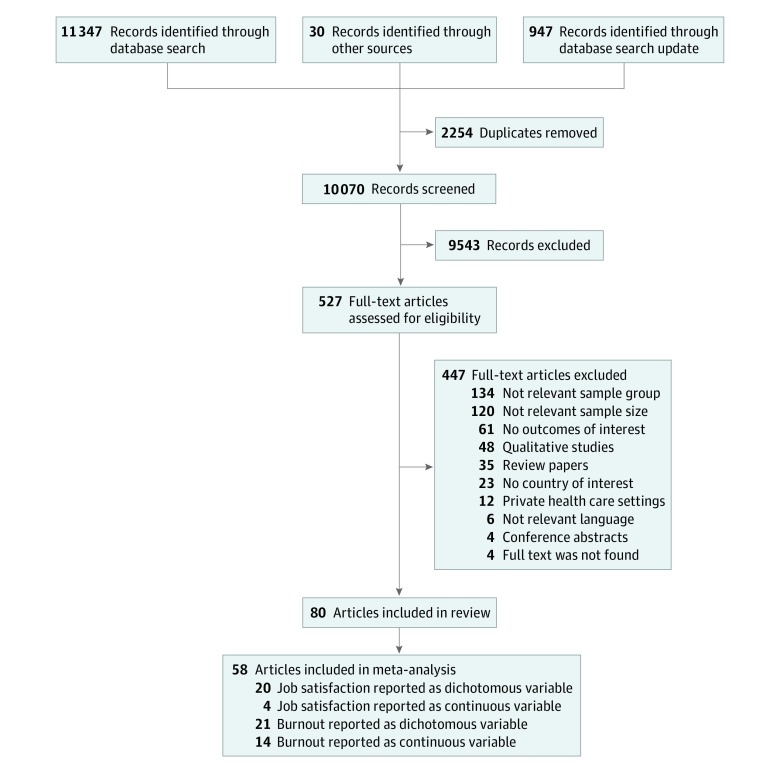
Flow Diagram

The original search was conducted in January 2018, and an update was performed in October 2018. Full texts were not available for 4 studies. In total, 80 articles, representing 79 studies, were deemed eligible for inclusion in the review. One study was reported in 2 records.^40,41^ The detailed selection process is presented in a flow diagram (Figure 1).

Studies were published between 1982 and 2018, primarily in English (65 studies [82%]), with others in Spanish (9 [11%]), Portuguese (2 [2%]), Russian (1 [1%]), Turkish (1 [1%]), and French (1 [1%]). Three studies (4%) used data from more than 1 country.^76,77,78^ In total, studies assessed 45 714 participants from 37 LMICs (eAppendix 2 in the Supplement). Only 1 study took place in a low-income country^52^; 2 were multicentered and included data from both low- and middle-income countries.^76,77^

**Figure 2.**
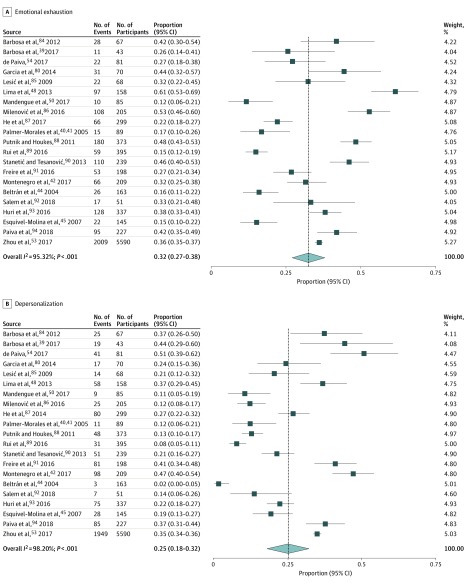
Meta-analysis of the Prevalence of Burnout Among Physicians and Dentists in Low-Income and Middle-Income Countries

Overall, 77 studies (97%) were cross-sectional surveys, 2 (3%) used mixed methods,^52,79^ and 1 (1%) was an observational cohort.^80^ In the cohort study, only baseline data were included in the analysis. Sample sizes varied between 40^81^ and 11 530^76^ participants, with a median (interquartile range) sample size of 198 (108-314.5) participants. The response rate varied between 12.3%^82^ and 97.4%.^83^ A summary of study characteristics is shown in eAppendix 3 in the Supplement. Of 26 studies reporting burnout results as dichotomous data, 21 studies used the 22-item Maslach Burnout Inventory–Human Services Survey and reported sufficient data to be included in the meta-analysis, with a total sample of 9092 participants. In the pooled random-effect estimates of prevalence in these 21 studies, 32% (95% CI, 27%-38%; *I*^2^ = 95.32%; *P* < .001) of physicians and dentists exceeded the high threshold for emotional exhaustion, and 25% (95% CI, 18%-32%; *I*^2^ = 98.20%; *P* < .001) were above the high threshold for depersonalization (Figure 2).^39,40,41,42,44,45,48,50,53,54,80,84,85,86,87,88,89,90,91,92,93,94^ In 20 studies, 33% (95% CI 22%-45%; *I*^2^ = 99.25%; *P* < .001) were below the low threshold for personal accomplishment (eAppendix 4 in the Supplement). Subgroup analyses suggested that the prevalence of burnout dimensions varied depending on the country’s geographical region (*P *for heterogeneity < .001), physician specialties (*P *for heterogeneity < .001), and thresholds used (emotional exhaustion and personal accomplishment: *P *for heterogeneity = .007; depersonalization: *P *for heterogeneity < .001). High levels of within-group heterogeneity and uneven covariate distribution among groups were present, demonstrating that these subgroups could not account for the variance between studies. Further, the metaregression showed that the prevalence of high emotional exhaustion was significantly higher in South America (regression coefficient, 0.259; 95% CI, 0.352-0.483; *P* = .03) and Southern Europe (regression coefficient, 0.337; 95% CI, 0.101-0.572; *P* = .008) compared with studies from other geographical regions used in the current metaregression. The study from Cameroon, Africa,^50^ was excluded from the metaregression because of the collinearity of results (eAppendix 4 in the Supplement).

**Figure 3.**
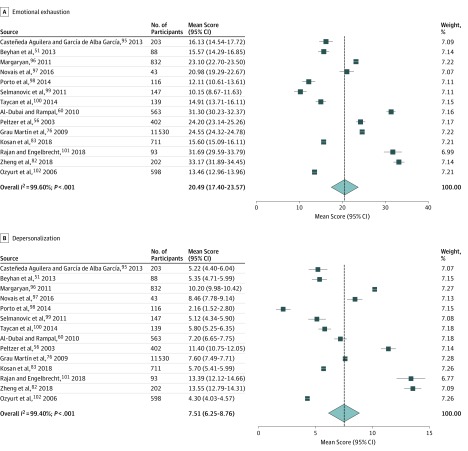
Meta-analysis of the Mean Score for Burnout Among Physicians and Dentists in Low-Income and Middle-Income Countries

Of 20 studies that reported burnout results as continuous data, 14 studies used a similar scale (Maslach Burnout Inventory–Human Services Survey or Maslach Burnout Inventory–General Survey) and reported sufficient data to be included in a meta-analysis with a total sample of 15 520 participants. The following random-effects estimates of the weighted mean scores were obtained: emotional exhaustion, 20.49 (95% CI, 17.40-23.57; *I*^2^ = 99.6%; *P* < .001); depersonalization, 7.51 (95% CI, 6.25-8.76; *I*^2^ = 99.4%; *P* < .001) (Figure 3).^51,56,60,76,82,83,95,96,97,98,99,100,101,102^ In 13 studies measuring personal accomplishment, the random-effects estimate of the weighted mean score was 28.92 (95% CI 23.54-34.29; *I*^2^ = 99.9%; *P* < .001) (eAppendix 4 in the Supplement). Heterogeneity was extremely high in all analyses. Subgroup analyses showed that mean values varied depending on the geographical region (*P *for heterogeneity < .001 ) and specialty (*P *for heterogeneity < .001). The results of subgroup analyses might be uncertain because of uneven covariate distribution among groups and an insufficient number of studies per group. Further, in the metaregression, the mean value for emotional exhaustion was significantly higher only in studies from China (regression coefficient, 23.02; 95% CI, 2.84-43.20; *P* = .03) and South Africa (regression coefficient, 17.76; 95% CI, 0.25-35.26; *P* = .047) (eAppendix 4 in the Supplement).

**Figure 4.**
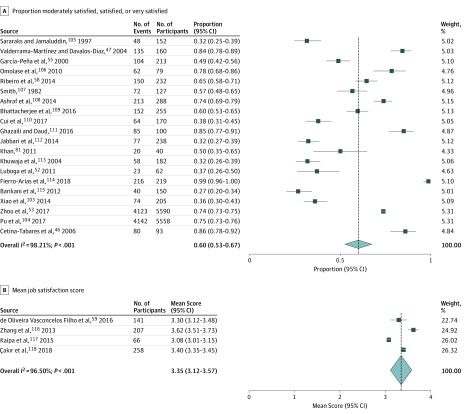
Meta-analysis of Job Satisfaction Among Physicians and Dentists in Low-Income and Middle-Income Countries A, The proportion of physicians’ and dentists’ job satisfaction is based on results provided as dichotomous data. B, The mean score of physicians’ and dentists’ job satisfaction is based on results provided as continuous data on a scale ranging from 1 to 5.

Of 21 studies that presented job satisfaction levels as dichotomous data, 20 studies were included in a meta-analysis, with a total sample of 14 113 participants. The number of moderately satisfied, satisfied, and very satisfied physicians and dentists and sample sizes were pooled, resulting in an overall weighted percentage of 60% (95% CI 53%-67%), representing 14 113 physicians and dentists with substantial heterogeneity (*I*^2^ = 98.21%; *P* < .001) (Figure 4A).^46,47,52,53,55,58,59,81,103,104,105,106,107,108,109,110,111,112,113,114,115,116,117,118^ Subgroup analyses found that job satisfaction levels varied depending on the country’s income group (*P *for heterogeneity = .005) and physician specialty (*P *for heterogeneity < .001) but not on region (*P *for heterogeneity = .19). The results of subgroup analyses might be uncertain because of an insufficient number of studies within each group. This concern was supported by a metaregression of dichotomous data, which suggested that job satisfaction levels did not differ significantly across countries with different income levels, but it was significantly higher among dermatologists (regression coefficient, 0.45; 95% CI, 0.13-0.89; *P* = .045) compared with other specialties (eAppendix 5 in the Supplement).

Of the 10 studies that presented job satisfaction scores as continuous data, 4 studies^59,116,117,118^ reported similar scales and had sufficient data to be included in a meta-analysis, with a total sample of 672 participants. Study results, which were reported based on a scale ranging from 1 (ie, extremely dissatisfied) to 5 (ie, extremely satisfied), were pooled, resulting in a mean value of 3.35 (95% CI, 3.12-3.57; *I*^2^ = 96.5%, *P* < .001) (Figure 4B).

The sensitivity analyses explored the robustness and stability of the meta-analyses on job satisfaction and burnout against studies with lower quality and in which health care setting was not reported. The pooled prevalence levels remained similar and still showed substantial heterogeneity (eAppendix 4 and eAppendix 5 in the Supplement). A sensitivity analysis excluding studies in which the sample included respondents who were not qualified physicians or dentists did not substantially change job burnout results. However, job satisfaction level increased to 83% (95% CI 63%-97%; *I*^2^ = 97.77%; *P* < .001) in a sensitivity analysis of 4 studies, suggesting that job satisfaction results might be sensitive to this criterion (eAppendix 5 in the Supplement).

Of 4 studies measuring job motivation, only 1 study^119^ presented quantifiable data. Data were reported based on the Warr-Cook-Wall likert scale from 1 (extremely dissatisfied) to 7 (extremely satisfied) with a total sample of 149 participants. The mean (SD) value of job motivation was 6.28 (0.97) and indicated that participants were generally motivated to do their work.

According to the Quality Assessment Tool for Observational Cohort and Cross-Sectional Studies, participation rate of eligible persons was at least 50% in 47 studies (59%), and 18 studies (23%) reported a sample size justification. Key potential confounding variables were measured and adjusted statistically in 40 studies (51%) (eAppendix 6 in the Supplement). Visual inspection of the funnel plots defined an asymmetry in all distributions for job satisfaction and burnout studies, although the Egger tests suggested the possibility of small-study effects (bias, −2.57; SE, 0.93; *P* = .01) only in the meta-analysis for job satisfaction reported as dichotomous data. In other cases, Egger tests did not show significant results and suggested little evidence for publication bias (eAppendix 4 and eAppendix 5 in the Supplement).

## Discussion

This study pooled findings from 79 unique studies with 45 714 participants. Considering emotional exhaustion as a core dimension of burnout,^120,121^ our findings suggest that 32% of physicians and dentists working mainly in middle-income countries experienced professional burnout. They also suggest that 60% of physicians and dentists working mainly in middle-income countries were satisfied with their job. In general, physicians and dentists working mainly in middle-income countries were motivated and pleased with their work.

The levels of burnout found primarily in middle-income countries varied across studies but were overall noticeably lower than those found in HICs. In France, the level of burnout is estimated to be 49%,^122^ in the United States, 50%,^123^ and in Austria, 51%.^124^ These results may be influenced by differences in cutoff points as well as a focus on particular specialties and the inclusion of medical residents in some surveys in HICs because physicians from emergency or surgical specialties and residents tend to show higher levels of burnout.^31,122,125,126,127,128,129^ These discrepancies highlight the importance of standardizing measurement tools and definitions of burnout.

The present meta-analysis showed that the prevalence of job burnout varied across different countries and geographical regions, which may be associated with cultural differences among countries and regions. To our knowledge, these differences have scarcely been explored in previous research but may play a significant role in understanding job morale. For example, Lo et al^130^ proposed that burnout in China has been affected by an ongoing shift from the traditional status of a physician as someone with a sacred mission to someone delivering and maintaining a medical business. Also, there is a Chinese philosophy of working to fulfill duty “without complaining [about] tiredness or fatigue until the end of… life,”^130^ which may influence the reporting of burnout symptoms.

The current study found a pooled prevalence of 60% job satisfaction. This is similar to the 59% reported in European hospitals^131^ and the medium to high prevalence reported in America,^132,133^ despite wide-ranging differences in health care systems between middle-income countries and HICs. These informal comparisons might suggest that physicians working in HICs and middle-income countries have similar satisfying and dissatisfying experiences in their work practice despite the fact that working conditions, rewards, and organizational structures are all expected to be poorer in middle-income countries. Some studies conducted in HICs^134,135,136,137^ explored the satisfaction of physicians focusing on particular specialties and working circumstances. They found that certain groups had much lower levels of job satisfaction, highlighting the need for detailed analyses and the consideration of specific subgroups and contexts.

In accordance with the findings of this study, the following suggestions are made. Although limiting workload and improving financial and nonfinancial rewards sound straightforward, it is difficult when resources are limited and demands are high. Still, meeting the demand by increasing the workload might result in an inefficient balance, as job morale drops and quality of care is compromised. Finding the right balance is surely necessary yet challenging and might vary by context. Awareness and support for physicians needs to be improved; interventions aimed at supporting these professionals should be popularized and funded by organizations. Increasing the resilience of medical students might be an effective way to improve the job morale of future physicians. Because monitoring physicians’ and dentists’ job morale level is crucial, standardized tools should be developed and disseminated. Further, findings in this review were informally compared with findings from several HICs, which provided no evidence that burnout and job satisfaction levels as indicators of job morale were lower in LMICs than in HICs. More research applying standardized methods in both LMICs and HICs is required to make definite conclusions.

To navigate practice implications, future studies should address several research gaps. First, evidence on job morale levels and associated factors is needed in low-income settings, particularly in Africa, Southern Europe, and Central, Southern, and Southeastern Asia. Second, similar methods should be used to make comparisons among countries. Third, nation- and culture-specific factors should be explored further by introducing additional normative variables or scrutinizing contextual features, which could be explored through qualitative research. Future research may also benefit from using study designs that allow for the examination of longitudinal changes in job morale.

### Strengths and Limitations

This study has a number of strengths. The review used a holistic approach, considering 3 indicators of job morale, ie, job burnout, job satisfaction, and job motivation, which took into account the lack of an overall standardized measure for job morale. We conducted a systematic and reproducible search of published literature. Robust statistical procedures were used, and included records were not limited to English-language reports.

The study also has several limitations. First, there were not enough results on job motivation to conduct a meta-analysis; therefore, the final interpretation of job morale was made on 2 other indicators (ie, job burnout and job satisfaction). Second, although sensitivity analyses that excluded studies involving other health care professionals and medical residents showed the stability of the job burnout meta-analyses, job satisfaction results increased when all participants were qualified physicians or dentists. Third, the included studies were of variable quality. However, sensitivity analyses excluding studies of lower quality did not substantially alter the results. Fourth, significant heterogeneity was observed across studies and not explained by subgroup analyses or metaregressions. The comparability of findings across the included studies may be limited owing to wide variability in job characteristics, cultural differences, and nation-specific circumstances. The influence of the sociocultural context may be lost when diverse studies are combined; however, this is a widely used approach and to some extent inevitable in reviews of findings from many countries. Moreover, heterogeneity is a central characteristic of prevalence meta-analyses and expected to be high.^138,139^ Fifth, despite the use of a comprehensive search strategy, almost all included studies were from middle-income countries, possibly reflecting the shortage of resources for such studies in low-income countries. This means that our findings cannot be generalized to low-income countries. Also, relatively fewer findings were available from Africa, Southern Europe, and Central, Southern, and Southeastern Asia, which made it challenging to generalize conclusions about LMICs.

## Conclusions

This systematic review and meta-analysis found that job morale among physicians and dentists working in mainly middle-income countries was overall rather positive. Considering the high heterogeneity and limited quality of included studies, conclusions are tentative. Future research should robustly explore job morale using standardized methods and measures, and more research should be conducted across low-income countries and particularly in African, Asian, and Southern European regions. Such positive morale may help to deliver good quality care and improve recruitment and retention and may be used as evidence to support any policies and interventions aiming to develop health care in LMICs.

## Notes

Supplement. **eAppendix 1. **Search Strategy **eAppendix 2.** Geographical Allocation of Included Studies **eAppendix 3.** Summary Table **eAppendix 4. **Burnout **eAppendix 5.** Job Satisfaction **eAppendix 6.** Risk of Bias of Included Studies **eReferences.**
