# Supplementary material for: Indicators Associated With Job Morale Among Physicians and Dentists in Low-Income and Middle-Income Countries: A Systematic Review and Meta-analysis
Source: JAMA Netw Open. 2020 Jan 10;3(1):e1913202. doi: 10.1001/jamanetworkopen.2019.13202 (PMC6991249; doi:10.1001/jamanetworkopen.2019.13202)
Supplement: Supplement. — eAppendix 1. Search Strategy eAppendix 2. Geographical Allocation of Included Studies eAppendix 3. Summary Table eAppendix 4. Burnout eAppendix 5. Job Satisfaction eAppendix 6. Risk of Bias of Included Studies eReferences. [file jamanetwopen-3-e1913202-s001.pdf]

Supplementary Online Content

Sabitova A, McGranahan R, Altamore F, Jovanovic N, Windle E, Priebe S. Indicators associated with job morale among physicians and dentists in low-income and middle-income countries: a systematic review and meta-analysis. *JAMA Netw Open*. 2020;3(1):e1913202. doi:10.1001/jamanetworkopen.2019.13202

eAppendix 1. Search Strategy

eAppendix 2. Geographical Allocation of Included Studies

eAppendix 3. Summary Table

eAppendix 4. Burnout

eAppendix 5. Job Satisfaction

eAppendix 6. Risk of Bias of Included Studies

eReferences.

This supplementary material has been provided by the authors to give readers additional information about their work.

eAppendix 1. Search Strategy

1. **Search terms**

morale OR well-being OR “well being” OR wellbeing OR “job satisfaction” OR burnout OR burn-out OR “burn out” OR “job motivation” OR resilience OR depression OR “depression symptoms” OR “moral distress” OR “psychological distress” OR “depressive symptoms”

AND

“health workers” OR “healthcare professionals” OR “medical doctors” OR physicians OR “medical specialists” OR clinicians OR “clinical professionals” OR “medical professionals” OR “healthcare specialists” OR audiologists OR allergists OR andrologists OR anaesthesiologists OR cardiologists OR dentists OR dermatologists OR endocrinologists OR epidemiologists OR “family doctors” OR gastroenterologists OR gynaecologists OR haematologists OR hepatologists OR immunologists OR “infectious disease specialists” OR “internal medicine specialists” OR internists OR neonatologist OR nephrologists OR neurologist OR neurosurgeons OR obstetricians OR oncologists OR ophthalmologists OR “orthopaedic surgeons” OR “ENT specialists” OR otolaryngologists OR perinatologists OR “paleo pathologists” OR parasitologists OR pathologists OR paediatricians OR physiologists OR physiatrists OR podiatrists OR psychiatrists OR pulmonologists OR radiologists OR rheumatologists OR surgeons OR urologists OR “emergency doctors”

AND

“low- and middle-income countries” OR LMICs OR “low and middle income countries” OR Afghanistan OR “Gambia The” OR Niger OR Benin OR Guinea OR Rwanda OR “Burkina Faso” OR Guniea-Bisau OR “Sierra Leone” OR Burundi OR Haiti OR Somalia OR Cambodia OR “Korea, Dem. Rep.” OR “South Sudan” OR “Central African Republic” OR Liberia OR Tanzania OR Chad OR Madagascar OR Togo OR Comoros OR Malawi OR Uganda OR “Congo, Dem. Rep.” OR Mali OR Zimbabwe OR Eritrea OR Mozambique OR Ethiopia OR Nepal OR Armenia OR Indonesia OR Samoa OR Bangladesh OR Kenya OR “Sao Tome and Principe” OR Bhutan OR Kiribati OR Senegal OR Bolivia OR Kosovo OR “Solomon Islands” OR “Cabo Verde” OR “Kyrgyz Republic” OR “Sri Lanka” OR Cameroon OR “Lao PDR” OR Sudan OR “Congo, Rep.” OR Lesotho OR Swaziland OR “Cote divoire” OR Mauritania OR “Syrian Arab Republic” OR Djibouti OR “Micronesia, Fed. Sts.” OR Tajikistan OR “Egypt, Arab Rep.” OR Moldova OR Timor-Leste OR “El Salvador” OR Morocco OR Ukraine OR Georgia OR Myanmar OR Uzbekistan OR Ghana OR Nicaragua OR Vanuatu OR Guatemala OR Nigeria OR Vietnam OR Guyana OR Pakistan OR “West Bank and Gaza” OR Honduras OR “Papua New Guinea” OR “Yemen, Rep.” OR India OR Philippines OR Zambia OR Albania OR Fiji OR Namibia OR Algeria OR Gabon OR Palau OR “American Samoa” OR Grenada OR Panama OR Angola OR “Iran, Islamic Rep.” OR Paraguay OR Azerbaijan OR Iraq OR Peru OR Belarus OR Jamaica OR Romania OR Belize OR Jordan OR Serbia OR “Bosnia and Herzegovina” OR Kazakhstan OR “South Africa” OR Botswana OR Lebanon OR “St. Lucia” OR Brazil OR Libya OR “St. Vincent and the Grenadines” OR Bulgaria OR “Macedonia, FYR” OR Suriname OR China OR Malaysia OR Thailand OR Colombia OR Maldives OR Tonga OR “Costa Rica” OR “Marshall Islands” OR Tunisia OR Cuba OR Mauritius OR Turkey OR Dominica OR Mexico OR Turkmenistan OR “Dominican Republic” OR Mongolia OR Tuvalu Ecuador OR Montenegro

© 2020 Sabitova A et al. *JAMA Network Open.*

2. Search strategies

2.1. Pubmed

#4 Add Search ((((morale[Title/Abstract] OR well-being[Title/Abstract] OR “well being”[Title/Abstract] OR wellbeing[Title/Abstract] OR “job satisfaction”[Title/Abstract] OR burnout[Title/Abstract] OR burn-out[Title/Abstract] OR “burn out”[Title/Abstract] OR “job motivation”[Title/Abstract] OR resilience[Title/Abstract] OR depression[Title/Abstract] OR “depression symptoms”[Title/Abstract] OR “moral distress”[Title/Abstract] OR “psychological distress”[Title/Abstract] OR “depressive symptoms”[Title/Abstract]))) AND ((“health workers”[Title/Abstract] OR “healthcare professionals”[Title/Abstract] OR “medical doctors”[Title/Abstract] OR physicians[Title/Abstract] OR “medical specialists”[Title/Abstract] OR clinicians[Title/Abstract] OR “clinical professionals”[Title/Abstract] OR “medical professionals”[Title/Abstract] OR “healthcare specialists”[Title/Abstract] OR audiologists[Title/Abstract] OR allergists[Title/Abstract] OR andrologists [Title/Abstract] OR anaesthesiologists [Title/Abstract] OR cardiologists[Title/Abstract] OR dentists[Title/Abstract] OR dermatologists[Title/Abstract] OR endocrinologists[Title/Abstract] OR epidemiologists[Title/Abstract] OR “family doctors”[Title/Abstract] OR gastroenterologists[Title/Abstract] OR gynaecologists[Title/Abstract] OR haematologists[Title/Abstract] OR hepatologists[Title/Abstract] OR immunologists[Title/Abstract] OR “infectious disease specialists”[Title/Abstract] OR “internal medicine specialists”[Title/Abstract] OR internists[Title/Abstract] OR neonatologist[Title/Abstract] OR nephrologists[Title/Abstract] OR neurologist[Title/Abstract] OR neurosurgeons[Title/Abstract] OR obstetricians[Title/Abstract] OR oncologists[Title/Abstract] OR ophthalmologists[Title/Abstract] OR “orthopaedic surgeons”[Title/Abstract] OR “ENT specialists”[Title/Abstract] OR otolaryngologists[Title/Abstract] OR perinatologists[Title/Abstract] OR “paleo pathologists”[Title/Abstract] OR parasitologists[Title/Abstract] OR pathologists[Title/Abstract] OR paediatricians[Title/Abstract] OR physiologists[Title/Abstract] OR physiatrists[Title/Abstract] OR podiatrists[Title/Abstract] OR psychiatrists[Title/Abstract] OR pulmonologists[Title/Abstract] OR radiologists[Title/Abstract] OR rheumatologists[Title/Abstract] OR surgeons[Title/Abstract] OR urologists[Title/Abstract] OR “emergency doctors”[Title/Abstract]))) AND (("low- and middle-income countries" OR lmics OR "low and middle income countries" OR afghanistan OR "Gambia The" OR niger OR benin OR guinea OR rwanda OR "Burkina Faso" OR guinea bissau OR "Sierra Leone" OR burundi OR haiti OR somalia OR cambodia OR "Korea, Dem. Rep." OR "South Sudan" OR "Central African Republic" OR liberia OR tanzania OR chad OR madagascar OR togo OR comoros OR malawi OR uganda OR "Congo, Dem. Rep." OR mali OR zimbabwe OR eritrea OR mozambique OR ethiopia OR nepal OR armenia OR indonesia OR samoa OR bangladesh OR kenya OR "Sao Tome and Principe" OR bhutan OR kiribati OR senegal OR bolivia OR kosovo OR "Solomon Islands" OR "Cabo Verde" OR "Kyrgyz Republic" OR "Sri Lanka" OR cameroon OR "Lao PDR" OR sudan OR "Congo, Rep." OR lesotho OR swaziland OR "Cote divoire" OR mauritania OR "Syrian Arab Republic" OR djibouti OR "Micronesia, Fed. Sts." OR tajikistan OR "Egypt, Arab Rep." OR moldova OR timor-leste OR "El Salvador" OR morocco OR ukraine OR georgia OR myanmar OR uzbekistan OR ghana OR nicaragua OR vanuatu OR guatemala OR nigeria OR vietnam OR guyana OR pakistan OR "West Bank and Gaza" OR honduras OR "Papua New Guinea" OR "Yemen, Rep." OR india OR philippines OR zambia OR albania OR fiji OR namibia OR algeria OR gabon OR palau OR "American Samoa" OR grenada OR panama OR angola OR "Iran, Islamic Rep." OR paraguay OR azerbaijan OR iraq OR peru OR belarus OR jamaica OR romania OR belize OR jordan OR serbia OR "Bosnia and Herzegovina" OR kazakhstan OR "South Africa" OR botswana OR lebanon OR "St. Lucia" OR brazil OR libya OR "St. Vincent and the Grenadines" OR bulgaria OR "Macedonia, FYR" OR suriname OR china OR malaysia OR thailand OR colombia OR maldives OR tonga OR "Costa Rica" OR "Marshall Islands" OR tunisia OR cuba OR mauritius OR turkey OR dominica OR mexico OR turkmenistan OR "Dominican Republic" OR mongolia OR tuvalu ecuador OR montenegro)) Sort by: PublicationDate 2475 06:23:48

© 2020 Sabitova A et al. *JAMA Network Open.*

#3 Add Search ("low- and middle-income countries" OR lmics OR "low and middle income countries" OR afghanistan OR "Gambia The" OR niger OR benin OR guinea OR rwanda OR "Burkina Faso" OR guinea bissau OR "Sierra Leone" OR burundi OR haiti OR somalia OR cambodia OR "Korea, Dem. Rep." OR "South Sudan" OR "Central African Republic" OR liberia OR tanzania OR chad OR madagascar OR togo OR comoros OR malawi OR uganda OR "Congo, Dem. Rep." OR mali OR zimbabwe OR eritrea OR mozambique OR ethiopia OR nepal OR armenia OR indonesia OR samoa OR bangladesh OR kenya OR "Sao Tome and Principe" OR bhutan OR kiribati OR senegal OR bolivia OR kosovo OR "Solomon Islands" OR "Cabo Verde" OR "Kyrgyz Republic" OR "Sri Lanka" OR cameroon OR "Lao PDR" OR sudan OR "Congo, Rep." OR lesotho OR swaziland OR "Cote divoire" OR mauritania OR "Syrian Arab Republic" OR djibouti OR "Micronesia, Fed. Sts." OR tajikistan OR "Egypt, Arab Rep." OR moldova OR timor-leste OR "El Salvador" OR morocco OR ukraine OR georgia OR myanmar OR uzbekistan OR ghana OR nicaragua OR vanuatu OR guatemala OR nigeria OR vietnam OR guyana OR pakistan OR "West Bank and Gaza" OR honduras OR "Papua New Guinea" OR "Yemen, Rep." OR india OR philippines OR zambia OR albania OR fiji OR namibia OR algeria OR gabon OR palau OR "American Samoa" OR grenada OR panama OR angola OR "Iran, Islamic Rep." OR paraguay OR azerbaijan OR iraq OR peru OR belarus OR jamaica OR romania OR belize OR jordan OR serbia OR "Bosnia and Herzegovina" OR kazakhstan OR "South Africa" OR botswana OR lebanon OR "St. Lucia" OR brazil OR libya OR "St. Vincent and the Grenadines" OR bulgaria OR "Macedonia, FYR" OR suriname OR china OR malaysia OR thailand OR colombia OR maldives OR tonga OR "Costa Rica" OR "Marshall Islands" OR tunisia OR cuba OR mauritius OR turkey OR dominica OR mexico OR turkmenistan OR "Dominican Republic" OR mongolia OR tuvalu ecuador OR montenegro) Sort by: PublicationDate 3321846 06:23:29

#2 Add Search (“health workers”[Title/Abstract] OR “healthcare professionals”[Title/Abstract] OR “medical doctors”[Title/Abstract] OR physicians[Title/Abstract] OR “medical specialists”[Title/Abstract] OR clinicians[Title/Abstract] OR “clinical professionals”[Title/Abstract] OR “medical professionals”[Title/Abstract] OR “healthcare specialists”[Title/Abstract] OR audiologists[Title/Abstract] OR allergists[Title/Abstract] OR andrologists [Title/Abstract] OR anaesthesiologists [Title/Abstract] OR cardiologists[Title/Abstract] OR dentists[Title/Abstract] OR dermatologists[Title/Abstract] OR endocrinologists[Title/Abstract] OR epidemiologists[Title/Abstract] OR “family doctors”[Title/Abstract] OR gastroenterologists[Title/Abstract] OR gynaecologists[Title/Abstract] OR haematologists[Title/Abstract] OR hepatologists[Title/Abstract] OR immunologists[Title/Abstract] OR “infectious disease specialists”[Title/Abstract] OR “internal medicine specialists”[Title/Abstract] OR internists[Title/Abstract] OR neonatologist[Title/Abstract] OR nephrologists[Title/Abstract] OR neurologist[Title/Abstract] OR neurosurgeons[Title/Abstract] OR obstetricians[Title/Abstract] OR oncologists[Title/Abstract] OR ophthalmologists[Title/Abstract] OR “orthopaedic surgeons”[Title/Abstract] OR “ENT specialists”[Title/Abstract] OR otolaryngologists[Title/Abstract] OR perinatologists[Title/Abstract] OR “paleo pathologists”[Title/Abstract] OR parasitologists[Title/Abstract] OR pathologists[Title/Abstract] OR paediatricians[Title/Abstract] OR physiologists[Title/Abstract] OR physiatrists[Title/Abstract] OR podiatrists[Title/Abstract] OR psychiatrists[Title/Abstract] OR pulmonologists[Title/Abstract] OR radiologists[Title/Abstract] OR rheumatologists[Title/Abstract] OR surgeons[Title/Abstract] OR urologists[Title/Abstract] OR “emergency doctors”[Title/Abstract]) Sort by: PublicationDate 601950 06:22:58

#1 Add Search (morale[Title/Abstract] OR well-being[Title/Abstract] OR “well being”[Title/Abstract] OR wellbeing[Title/Abstract] OR “job satisfaction”[Title/Abstract] OR burnout[Title/Abstract] OR burn-out[Title/Abstract] OR “burn out”[Title/Abstract] OR “job motivation”[Title/Abstract] OR resilience[Title/Abstract] OR depression[Title/Abstract] OR “depression symptoms”[Title/Abstract] OR “moral distress”[Title/Abstract] OR “psychological distress”[Title/Abstract] OR “depressive symptoms”[Title/Abstract]) Sort by: PublicationDate 393635 06:22:33

© 2020 Sabitova A et al. *JAMA Network Open.*

2.2. Scopus
4231 results

(KEY (morale OR well-being OR "well being" OR wellbeing OR "job satisfaction" OR burnout OR burn-out OR "burn out" OR "job motivation" OR resilience OR depression OR "depression symptoms" OR "moral distress" OR "psychological distress" OR "depressive symptoms")) AND (KEY ("health workers" OR "healthcare professionals" OR "medical doctors" OR physicians OR "medical specialists" OR clinicians OR "clinical professionals" OR "medical professionals" OR "healthcare specialists" OR audiologists OR allergists OR andrologists OR anaesthesiologists OR cardiologists OR dentists OR dermatologists OR endocrinologists OR epidemiologists OR “family doctors” OR gastroenterologists OR gynaecologists OR haematologists OR hepatologists OR immunologists OR “infectious disease specialists” OR “internal medicine specialists” OR internists OR neonatologist OR nephrologists OR neurologist OR neurosurgeons OR obstetricians OR oncologists OR ophthalmologists OR “orthopaedic surgeons” OR “ENT specialists” OR otolaryngologists OR perinatologists OR “paleo pathologists” OR parasitologists OR pathologists OR paediatricians OR physiologists OR physiatrists OR podiatrists OR psychiatrists OR pulmonologists OR radiologists OR rheumatologists OR surgeons OR urologists OR “emergency doctors”)) AND (EXCLUDE (AFFILCOUNTRY , "United States") OR EXCLUDE (AFFILCOUNTRY , "United Kingdom") OR EXCLUDE (AFFILCOUNTRY , "Australia") OR EXCLUDE (AFFILCOUNTRY , "Germany") OR EXCLUDE (AFFILCOUNTRY , "Canada") OR EXCLUDE (AFFILCOUNTRY , "Netherlands") OR EXCLUDE (AFFILCOUNTRY , "France") OR EXCLUDE (AFFILCOUNTRY , "Italy") OR EXCLUDE (AFFILCOUNTRY , "Switzerland") OR EXCLUDE (AFFILCOUNTRY , "Spain") OR EXCLUDE (AFFILCOUNTRY , "Sweden") OR EXCLUDE (AFFILCOUNTRY , "Japan") OR EXCLUDE (AFFILCOUNTRY , "Israel") OR EXCLUDE (AFFILCOUNTRY , "Norway") OR EXCLUDE (AFFILCOUNTRY , "New Zealand") OR EXCLUDE (AFFILCOUNTRY , "Belgium") OR EXCLUDE (AFFILCOUNTRY , "Denmark") OR EXCLUDE (AFFILCOUNTRY , "Finland") OR EXCLUDE (AFFILCOUNTRY , "Ireland") OR EXCLUDE (AFFILCOUNTRY , "Austria") OR EXCLUDE (AFFILCOUNTRY , "Poland") OR EXCLUDE (AFFILCOUNTRY , "South Korea") OR EXCLUDE (AFFILCOUNTRY , "Greece") OR EXCLUDE (AFFILCOUNTRY , "Singapore") OR EXCLUDE (AFFILCOUNTRY , "Hungary") OR EXCLUDE (AFFILCOUNTRY , "Portugal") OR EXCLUDE (AFFILCOUNTRY , "Saudi Arabia") OR EXCLUDE (AFFILCOUNTRY , "United Arab Emirates") OR EXCLUDE (AFFILCOUNTRY , "Luxembourg") OR EXCLUDE (AFFILCOUNTRY , "Malta")) AND (EXCLUDE (SRCTYPE , "Undefined")) AND (EXCLUDE (AFFILCOUNTRY , "Hong Kong") OR EXCLUDE (AFFILCOUNTRY , "Slovenia") OR EXCLUDE (AFFILCOUNTRY , "Romania") OR EXCLUDE (AFFILCOUNTRY , "Lithuania") OR EXCLUDE (AFFILCOUNTRY , "Bulgaria") OR EXCLUDE (AFFILCOUNTRY , "Estonia"))

2.3. PsycINFO
Search Screen - Advanced Search

Database - PsycINFO 1,710

S3 TX “low- and middle-income countries” OR LMICs OR “low and middle income countries” OR Afghanistan OR “GambiaThe” OR Niger OR Benin OR Guinea OR Rwanda OR “Burkina Faso” OR Guniea-Bisau OR “Sierra Leone” OR Burundi OR Haiti OR Somalia OR Cambodia OR “Korea, Dem. Rep.” OR “South Sudan” OR “Central African Republic” OR Liberia OR Tanzania OR Chad OR Madagascar OR Togo OR Comoros OR Malawi OR Uganda OR “Congo, Dem. Rep.” OR Mali OR Zimbabwe OR Eritrea OR Mozambique OR Ethiopia OR Nepal OR Armenia OR Indonesia OR Samoa OR Bangladesh OR Kenya OR “Sao Tome and Principe” OR Bhutan OR Kiribati OR Senegal OR Bolivia OR Kosovo OR “Solomon Islands” OR “Cabo Verde” OR “Kyrgyz Republic” OR “Sri Lanka” OR Cameroon OR “Lao PDR” OR Sudan OR “Congo, Rep.” OR Lesotho OR Swaziland OR “Cote divoire” OR Mauritania OR “Syrian Arab Republic” OR Djibouti OR “Micronesia, Fed. Sts.” OR

© 2020 Sabitova A et al. *JAMA Network Open.*

Tajikistan OR “Egypt, Arab Rep.” OR Moldova OR Timor-Leste OR “El Salvador” OR Morocco OR Ukraine OR Georgia OR Myanmar OR Uzbekistan OR Ghana OR Nicaragua OR Vanuatu OR Guatemala OR Nigeria OR Vietnam OR Guyana OR Pakistan OR “West Bank and Gaza” OR Honduras OR “Papua New Guinea” OR “Yemen, Rep.” OR India OR Philippines OR Zambia OR Albania OR Fiji OR Namibia OR Algeria OR Gabon OR Palau OR “American Samoa” OR Grenada OR Panama OR Angola OR “Iran, Islamic Rep.” OR Paraguay OR Azerbaijan OR Iraq OR Peru OR Belarus OR Jamaica OR Romania OR Belize OR Jordan OR Serbia OR “Bosnia and Herzegovina” OR Kazakhstan OR “South Africa” OR Botswana OR Lebanon OR “St. Lucia” OR Brazil OR Libya OR “St. Vincent and the Grenadines” OR Bulgaria OR “Macedonia, FYR” OR Suriname OR China OR Malaysia OR Thailand OR Colombia OR Maldives OR Tonga OR “Costa Rica” OR “Marshall Islands” OR Tunisia OR Cuba OR Mauritius OR Turkey OR Dominica OR Mexico OR Turkmenistan OR “Dominican Republic” OR Mongolia OR Tuvalu Ecuador OR Montenegro Search modes - Boolean/Phrase Interface - EBSCOhost Research Databases

Search Screen - Advanced Search

Database - PsycINFO 426,644

S2 TX “health workers” OR “healthcare professionals” OR “medical doctors” OR physicians OR “medical specialists” OR clinicians OR “clinical professionals” OR “medical professionals” OR “healthcare specialists” OR audiologists OR allergists OR andrologists OR anaesthesiologists OR cardiologists OR dentists OR dermatologists OR endocrinologists OR epidemiologists OR “family doctors” OR gastroenterologists OR gynaecologists OR haematologists OR hepatologists OR immunologists OR “infectious disease specialists” OR “internal medicine specialists” OR internists OR neonatologist OR nephrologists OR neurologist OR neurosurgeons OR obstetricians OR oncologists OR ophthalmologists OR “orthopaedic surgeons” OR “ENT specialists” OR otolaryngologists OR perinatologists OR “paleo pathologists” OR parasitologists OR pathologists OR paediatricians OR physiologists OR physiatrists OR podiatrists OR psychiatrists OR pulmonologists OR radiologists OR rheumatologists OR surgeons OR urologists OR “emergency doctors” Search modes - Boolean/Phrase Interface - EBSCOhost Research Databases

Search Screen - Advanced Search

Database - PsycINFO 256,488

S1 KW morale OR well-being OR “well being” OR wellbeing OR “job satisfaction” OR burnout OR burn-out OR “burn out” OR “job motivation” OR resilience OR depression OR “depression symptoms” OR “moral distress” OR “psychological distress” OR “depressive symptoms” Search modes - Boolean/Phrase Interface - EBSCOhost Research Databases

Search Screen - Advanced Search

Database - PsycINFO 183,349

2.4. Embase

#1 AND #2 AND #3

37

#3

('low- and middle-income countries' OR lmics OR 'low and middle income countries' OR 'afghanistan'/exp OR afghanistan OR 'gambiathe' OR 'niger'/exp OR niger OR 'benin'/exp OR benin OR 'guinea'/exp OR guinea OR 'rwanda'/exp OR rwanda OR 'burkina faso'/exp OR 'burkina faso' OR

© 2020 Sabitova A et al. *JAMA Network Open.*

'guniea bisau' OR 'sierra leone'/exp OR 'sierra leone' OR 'burundi'/exp OR burundi OR 'haiti'/exp OR haiti OR 'somalia'/exp OR somalia OR 'cambodia'/exp OR cambodia OR 'korea, dem. rep.' OR 'south sudan'/exp OR 'south sudan' OR 'central african republic'/exp OR 'central african republic' OR 'liberia'/exp OR liberia OR 'tanzania'/exp OR tanzania OR 'chad'/exp OR chad OR 'madagascar'/exp OR madagascar OR 'togo'/exp OR togo OR 'comoros'/exp OR comoros OR 'malawi'/exp OR malawi OR 'uganda'/exp OR uganda OR 'congo, dem. rep.' OR 'mali'/exp OR mali OR 'zimbabwe'/exp OR zimbabwe OR 'eritrea'/exp OR eritrea OR 'mozambique'/exp OR mozambique OR 'ethiopia'/exp OR ethiopia OR 'nepal'/exp OR nepal OR 'armenia'/exp OR armenia OR 'indonesia'/exp OR indonesia OR 'samoa'/exp OR samoa OR 'bangladesh'/exp OR bangladesh OR 'kenya'/exp OR kenya OR 'sao tome and principe'/exp OR 'sao tome and principe' OR 'bhutan'/exp OR bhutan OR 'kiribati'/exp OR kiribati OR 'senegal'/exp OR senegal OR 'bolivia'/exp OR bolivia OR 'kosovo'/exp OR kosovo OR 'solomon islands'/exp OR 'solomon islands' OR 'cabo verde'/exp OR 'cabo verde' OR 'kyrgyz republic'/exp OR 'kyrgyz republic' OR 'sri lanka'/exp OR 'sri lanka' OR 'cameroon'/exp OR cameroon OR 'lao pdr' OR 'sudan'/exp OR sudan OR 'congo, rep.' OR 'lesotho'/exp OR lesotho OR 'swaziland'/exp OR swaziland OR 'cote divoire' OR 'mauritania'/exp OR mauritania OR 'syrian arab republic'/exp OR 'syrian arab republic' OR 'djibouti'/exp OR djibouti OR 'micronesia, fed. sts.' OR 'tajikistan'/exp OR tajikistan OR 'egypt, arab rep.' OR 'moldova'/exp OR moldova OR 'timor leste'/exp OR 'timor leste' OR 'el salvador'/exp OR 'el salvador' OR 'morocco'/exp OR morocco OR 'ukraine'/exp OR ukraine OR 'georgia'/exp OR georgia OR 'myanmar'/exp OR myanmar OR 'uzbekistan'/exp OR uzbekistan OR 'ghana'/exp OR ghana OR 'nicaragua'/exp OR nicaragua OR 'vanuatu'/exp OR vanuatu OR 'guatemala'/exp OR guatemala OR 'nigeria'/exp OR nigeria OR 'vietnam'/exp OR vietnam OR 'guyana'/exp OR guyana OR 'pakistan'/exp OR pakistan OR 'west bank and gaza' OR 'honduras'/exp OR honduras OR 'papua new guinea'/exp OR 'papua new guinea' OR 'yemen, rep.' OR 'india'/exp OR india OR 'philippines'/exp OR philippines OR 'zambia'/exp OR zambia OR 'albania'/exp OR albania OR 'fiji'/exp OR fiji OR 'namibia'/exp OR namibia OR 'algeria'/exp OR algeria OR 'gabon'/exp OR gabon OR 'palau'/exp OR palau OR 'american samoa'/exp OR 'american samoa' OR 'grenada'/exp OR grenada OR 'panama'/exp OR panama OR 'angola'/exp OR angola OR 'iran, islamic rep.' OR 'paraguay'/exp OR paraguay OR 'azerbaijan'/exp OR azerbaijan OR 'iraq'/exp OR iraq OR 'peru'/exp OR peru OR 'belarus'/exp OR belarus OR 'jamaica'/exp OR jamaica OR 'romania'/exp OR romania OR 'belize'/exp OR belize OR 'jordan'/exp OR jordan OR 'serbia'/exp OR serbia OR 'bosnia and herzegovina'/exp OR 'bosnia and herzegovina' OR 'kazakhstan'/exp OR kazakhstan OR 'south africa'/exp OR 'south africa' OR 'botswana'/exp OR botswana OR 'lebanon'/exp OR lebanon OR 'st. lucia'/exp OR 'st. lucia' OR 'brazil'/exp OR brazil OR 'libya'/exp OR libya OR 'st. vincent and the grenadines'/exp OR 'st. vincent and the grenadines' OR 'bulgaria'/exp OR bulgaria OR 'macedonia, fyr' OR 'suriname'/exp OR suriname OR 'china'/exp OR china OR 'malaysia'/exp OR malaysia OR 'thailand'/exp OR thailand OR 'colombia'/exp OR colombia OR 'maldives'/exp OR maldives OR 'tonga'/exp OR tonga OR 'costa rica'/exp OR 'costa rica' OR 'marshall islands'/exp OR 'marshall islands' OR 'tunisia'/exp OR tunisia OR 'cuba'/exp OR cuba OR 'mauritius'/exp OR mauritius OR 'turkey'/exp OR turkey OR 'dominica'/exp OR dominica OR 'mexico'/exp OR mexico OR 'turkmenistan'/exp OR turkmenistan OR 'dominican republic'/exp OR 'dominican republic' OR 'mongolia'/exp OR mongolia OR 'tuvalu'/exp OR tuvalu) AND ('ecuador'/exp OR ecuador) OR 'montenegro'/exp OR montenegro

13,077

#2

'health workers' OR 'healthcare professionals' OR 'medical doctors' OR 'physicians'/exp OR physicians OR 'medical specialists' OR clinicians OR 'clinical professionals' OR 'medical professionals' OR 'healthcare specialists' OR 'audiologists'/exp OR audiologists OR 'allergists'/exp OR allergists OR andrologists OR anaesthesiologists OR 'cardiologists'/exp OR cardiologists OR 'dentists'/exp OR dentists OR 'dermatologists'/exp OR dermatologists OR 'endocrinologists'/exp OR endocrinologists OR 'epidemiologists'/exp OR epidemiologists OR 'family doctors' OR

© 2020 Sabitova A et al. *JAMA Network Open.*

'gastroenterologists'/exp OR gastroenterologists OR gynaecologists OR 'haematologists'/exp OR haematologists OR hepatologists OR 'immunologists'/exp OR immunologists OR 'infectious disease specialists' OR 'internal medicine specialists' OR internists OR 'neonatologist'/exp OR neonatologist OR 'nephrologists'/exp OR nephrologists OR 'neurologist'/exp OR neurologist OR 'neurosurgeons'/exp OR neurosurgeons OR 'obstetricians'/exp OR obstetricians OR 'oncologists'/exp OR oncologists OR 'ophthalmologists'/exp OR ophthalmologists OR 'orthopaedic surgeons' OR 'ent specialists' OR 'otolaryngologist'/exp OR otolaryngologist OR perinatologists OR 'paleo pathologists' OR parasitologists OR 'pathologists'/exp OR pathologists OR 'paediatricians'/exp OR paediatricians OR physiologists OR 'physiatrists'/exp OR physiatrists OR podiatrists OR psychiatrists OR 'pulmonologists'/exp OR pulmonologists OR 'radiologists'/exp OR radiologists OR 'rheumatologists'/exp OR rheumatologists OR 'surgeons'/exp OR surgeons OR 'urologists'/exp OR urologists OR 'emergency doctors'

1,647,549

#1

'morale'/exp OR morale OR 'well being'/exp OR 'well being' OR 'wellbeing'/exp OR wellbeing OR 'job satisfaction'/exp OR 'job satisfaction' OR 'burnout'/exp OR burnout OR 'burn out' OR 'job motivation'/exp OR 'job motivation' OR 'resilience'/exp OR resilience OR 'depression'/exp OR depression OR 'depression symptoms' OR 'moral distress'/exp OR 'moral distress' OR 'psychological distress'/exp OR 'psychological distress' OR 'depressive symptoms'/exp OR 'depressive symptoms'

2.5. The Cochrane Library

#1

morale or well-being or "well being" or wellbeing or "job satisfaction" or burnout or burn-out or "burn out" or "job motivation" or resilience or depression or "depression symptoms" or "moral distress" or "psychological distress" or "depressive symptoms":ti,ab,kw (Word variations have been searched)

56503

#2

"health workers" or "healthcare professionals" or "medical doctors" or physicians or "medical specialists" or clinicians or "clinical professionals" or "medical professionals" or "healthcare specialists" or audiologists or allergists or andrologists or anaesthesiologists or cardiologists or dentists or dermatologists or endocrinologists or epidemiologists or "family doctors" or gastroenterologists or gynaecologists or haematologists or hepatologists or immunologists or "infectious disease specialists" or "internal medicine specialists" or internists or neonatologist or nephrologists or neurologist or neurosurgeons or obstetricians or oncologists or ophthalmologists or "orthopaedic surgeons" or "ENT specialists" or otolaryngologists or perinatologists or "paleo pathologists" or parasitologists or pathologists or paediatricians or physiologists or physiatrists or podiatrists or psychiatrists or pulmonologists or radiologists or rheumatologists or surgeons or urologists or "emergency doctors":ti,ab,kw (Word variations have been searched)

63443

#3

"low- and middle-income countries" or LMICs or "low and middle income countries" or Afghanistan or "GambiaThe" or Niger or Benin or Guinea or Rwanda or "Burkina Faso" or Guniea-Bisau or "Sierra Leone" or Burundi or Haiti or Somalia or Cambodia or "Korea, Dem. Rep." or "South Sudan" or "Central African Republic" or Liberia or Tanzania or Chad or Madagascar or Togo or Comoros or

© 2020 Sabitova A et al. *JAMA Network Open.*

Malawi or Uganda or "Congo, Dem. Rep." or Mali or Zimbabwe or Eritrea or Mozambique or Ethiopia or Nepal or Armenia or Indonesia or Samoa or Bangladesh or Kenya or "Sao Tome and Principe" or Bhutan or Kiribati or Senegal or Bolivia or Kosovo or "Solomon Islands" or "Cabo Verde" or "Kyrgyz Republic" or "Sri Lanka" or Cameroon or "Lao PDR" or Sudan or "Congo, Rep." or Lesotho or Swaziland or "Cote divoire" or Mauritania or "Syrian Arab Republic" or Djibouti or "Micronesia, Fed. Sts." or Tajikistan or "Egypt, Arab Rep." or Moldova or Timor-Leste or "El Salvador" or Morocco or Ukraine or Georgia or Myanmar or Uzbekistan or Ghana or Nicaragua or Vanuatu or Guatemala or Nigeria or Vietnam or Guyana or Pakistan or "West Bank and Gaza" or Honduras or "Papua New Guinea" or "Yemen, Rep." or India or Philippines or Zambia or Albania or Fiji or Namibia or Algeria or Gabon or Palau or "American Samoa" or Grenada or Panama or Angola or "Iran, Islamic Rep." or Paraguay or Azerbaijan or Iraq or Peru or Belarus or Jamaica or Romania or Belize or Jordan or Serbia or "Bosnia and Herzegovina" or Kazakhstan or "South Africa" or Botswana or Lebanon or "St. Lucia" or Brazil or Libya or "St. Vincent and the Grenadines" or Bulgaria or "Macedonia, FYR" or Suriname or China or Malaysia or Thailand or Colombia or Maldives or Tonga or "Costa Rica" or "Marshall Islands" or Tunisia or Cuba or Mauritius or Turkey or Dominica or Mexico or Turkmenistan or "Dominican Republic" or Mongolia or Tuvalu Ecuador or Montenegro:ti,ab,kw (Word variations have been searched)

40380

#4

#1 and #2 and #3

239

2.6. Web of Science

# 4	2,653	#3 AND #2 AND #1<br><br>*Indexes=SCI-EXPANDED, SSCI, A&HCI, CPCI-S, CPCI-SSH, ESCI Timespan=All years*	Edit
# 3	2,097,264	TS=(“low- and middle-income countries” OR LMICs OR “low and middle income countries” OR Afghanistan OR “GambiaThe” OR Niger OR Benin OR Guinea OR Rwanda OR “Burkina Faso” OR Guniea-Bisau OR “Sierra Leone” OR Burundi OR Haiti OR Somalia OR Cambodia OR “Korea, Dem. Rep.” OR “South Sudan” OR “Central African Republic” OR Liberia OR Tanzania OR Chad OR Madagascar OR Togo OR Comoros OR Malawi OR Uganda OR “Congo, Dem. Rep.” OR Mali OR Zimbabwe OR Eritrea OR Mozambique OR Ethiopia OR Nepal OR Armenia OR Indonesia OR Samoa OR Bangladesh OR Kenya OR “Sao Tome and Principe” OR Bhutan OR Kiribati OR Senegal OR Bolivia OR Kosovo OR “Solomon Islands” OR “Cabo Verde” OR “Kyrgyz Republic” OR “Sri Lanka” OR Cameroon OR “Lao PDR” OR Sudan OR “Congo, Rep.” OR Lesotho OR Swaziland OR “Cote divoire” OR Mauritania OR “Syrian Arab Republic” OR Djibouti OR “Micronesia, Fed. Sts.” OR Tajikistan OR “Egypt, Arab Rep.” OR Moldova OR Timor-Leste OR “El Salvador” OR Morocco OR Ukraine OR Georgia OR Myanmar OR Uzbekistan OR Ghana OR Nicaragua OR Vanuatu OR Guatemala OR	Edit

Nigeria OR Vietnam OR Guyana OR Pakistan OR “West Bank and Gaza” OR Honduras OR “Papua New Guinea” OR “Yemen, Rep.” OR India OR Philippines OR Zambia OR Albania OR Fiji OR Namibia OR Algeria OR Gabon OR Palau OR “American Samoa” OR Grenada OR Panama OR Angola OR “Iran, Islamic Rep.” OR Paraguay OR Azerbaijan OR Iraq OR Peru OR Belarus OR Jamaica OR Romania OR Belize OR Jordan OR Serbia OR “Bosnia and Herzegovina” OR Kazakhstan OR “South Africa” OR Botswana OR Lebanon OR “St. Lucia” OR Brazil OR Libya OR “St. Vincent and the Grenadines” OR Bulgaria OR “Macedonia, FYR” OR Suriname OR China OR Malaysia OR Thailand OR Colombia OR Maldives OR Tonga OR “Costa Rica” OR “Marshall Islands” OR Tunisia OR Cuba OR Mauritius OR Turkey OR Dominica OR Mexico OR Turkmenistan OR “Dominican Republic” OR Mongolia OR Tuvalu Ecuador OR Montenegro)

Indexes=SCI-EXPANDED, SSCI, A&HCI, CPCI-S, CPCI-SSH, ESCI Timespan=All years

2 752,444 TS = (“health workers” OR “healthcare professionals” OR “medical doctors” OR physicians OR “medical specialists” OR clinicians OR “clinical professionals” OR “medical professionals” OR “healthcare specialists” OR audiologists OR allergists OR andrologists OR anaesthesiologists OR cardiologists OR dentists OR dermatologists OR endocrinologists OR epidemiologists OR “family doctors” OR gastroenterologists OR gynaecologists OR haematologists OR hepatologists OR immunologists OR “infectious disease specialists” OR “internal medicine specialists” OR internists OR neonatologist OR nephrologists OR neurologist OR neurosurgeons OR obstetricians OR oncologists OR ophthalmologists OR “orthopaedic surgeons” OR “ENT specialists” OR otolaryngologist OR perinatologists OR “paleo pathologists” OR parasitologists OR pathologists OR paediatricians OR physiologists OR physiatrists OR podiatrists OR psychiatrists OR pulmonologists OR radiologists OR rheumatologists OR surgeons OR urologists OR “emergency doctors”) Edit

Indexes=SCI-EXPANDED, SSCI, A&HCI, CPCI-S, CPCI-SSH, ESCI Timespan=All years

1 862,434 TS=(morale OR well-being OR well being OR wellbeing OR job satisfaction OR burnout OR burn-out OR burn out OR job motivation OR resilience OR depression OR depression symptoms OR moral distress OR psychological distress OR depressive symptoms)

Indexes=SCI-EXPANDED, SSCI, A&HCI, CPCI-S, CPCI-SSH, ESCI Timespan=All years

© 2020 Sabitova A et al. *JAMA Network Open.*

3. Summary table of STATA commands used for the meta-analysis

Command	Aim
metaprop	to perform meta-analysis of dichotomous data
metaan, metan	to perform meta-analysis of continuous data
ftt	to perform the Freeman-Turkey double arcsine transformation
cimethod(score)	to compute the study specific confidence intervals using the score method
cimethod(exact)	to compute the study specific confidence intervals using the exact method
ml, random	to create a random-effects model
metareg	to perform meta-regression
metafunnel	to plot funnel plots
metabias	to perform statistical tests for funnel plot asymmetry

**Job satisfaction (dichotomous)**	metaprop Satisfied Samplesize, random ftt cimethod(score) label(namevar=Title, yearvar=Year)// metaprop Satisfied Samplesize, random by(Incomegroup) ftt cimethod(score) label(namevar=Title, yearvar=Year)// metaprop Satisfied Samplesize, random ftt cimethod(score) label(namevar=Title, yearvar=Year) // metabias _ES _seES, egger// tabulate Incomegroup, generate(Incomegroup)// metareg _ES Incomegroup1 Incomegroup2 Incomegroup3, wsse(_seES) graph//
**Job satisfaction (continues)**	gen SE=SD/sqrt(Samplesize)// metan Mean SE, ml label(namevar=Author, yearvar=Year)
**Burnout (dichotomous)**	metaprop EEn Samplesizen, random cimethod(exact) label(namevar=Author, yearvar=Year)// metaprop EEn Samplesizen, random by(Region) cimethod(exact) label(namevar=Author, yearvar=Year)// metaprop EEn Samplesizen, random by(ThresholdEE) cimethod(exact) label(namevar=Author, yearvar=Year)// metaprop DPn Samplesizen, random by(ThresholdDP) cimethod(exact) label(namevar=Author, yearvar=Year)// metaprop PAn Samplesizen, random by(ThresholdPA) cimethod(exact) label(namevar=Author, yearvar=Year)// metabias _ES _seES, egger// metafunnel _ES _seES// tabulate Region, generate(Region)// metareg _ES Region1 Region 2 Region 3, Region4, Region5, Region6, wsse(_seES) graph//
**Burnout (continuous)**	gen SE=SD/sqrt(Samplesize)// metan EEMean SEEE,random label(namevar=Author, yearvar=Year)// metan EEMean SEEE,random by(Incomegroup) label(namevar=Author, yearvar=Year)// metafunnel mean SE// tabulate Region, generate(Region)// metareg EEMean Region1 Region2 Region3 Region4 Region5 Region6, wsse(SEEE)//

© 2020 Sabitova A et al. *JAMA Network Open.*

eAppendix 2. Geographical Allocation of Included Studies

Geographical region		Country (N of studies)
Africa		South Africa (2 studies) ; Cameroon (1 study); Uganda (1 study); Nigeria (1 study)
Asia	Central Asia	Kazakhstan (1 study)
	Western Asia	Armenia (1 study); Iraq (1 study); Yemen (1 study); Turkey (10 studies); Lebanon (1 study); Yemen (1 study)
	Southern Asia	India (3 studies); Pakistan (4 studies); Iran (2 studies);
	Eastern Asia	China (15 studies)
	South-Eastern Asia	Malaysia (4 studies); Thailand (1 study)
America	Central America	Guatemala (2 studies); Costa Rica (1 study); El Salvador (1 study); Honduras (1 study); Nicaragua (1 study); Panama (1 study); Mexico (11 studies)
	South America	Brazil (14 studies); Argentina (3 studies); Colombia (2 studies); Ecuador (2 studies); Peru (2 studies); Bolivia (1 study); Paraguay (1 study);
	Caribbean	Cuba (1 study); Dominican Republic (1 study)
Europe	Eastern	Russia (4 studies); Romania (1 study)
	Southern	Bosnia and Herzegovina (2 studies); Serbia (3 studies)

© 2020 Sabitova A et al. *JAMA Network Open.*

eAppendix 3. Summary Table

Study/ Country (income group)	Sampling	Study population	Sample size (n) and characteristics (gender and age range/mean)	Response rate	Outcome(s) of interest	Measure/ assessment instrument	Prevalence Mean(SD) / % of high scores
Aguilera and De Alba Garcia, 2013[1]/ Mexico (upper-middle income)	not stated	dentists (not yet graduates were excluded from the study)	203<br>males 56.0% females 44%<br>not stated/41.5±10.1	88,3%	burnout (B)	Maslach Burnout Inventory - Human Services Survey (MBI-HSS)	EE=16.13(11.54); DP=5.22(5.93); PA=39.25(11.24).
Barbosa et al., 2012[2]/ Brazil (upper-middle income)	not stated	ICUs physicians (77.61% had a specialist degree)	67<br>males 44.78% females 55.22%<br>not stated/43.85±8.95	not stated	burnout (B)	Maslach Burnout Inventory - Human Services Survey (MBI-HSS)	EE=41.73%; DP=37.31%;<br>PA=58.2%.
Barbosa et al., 2017[3]/ Brazil (upper-middle income)	Random	anaesthesiologists (97.68% had a specialist degree)	43<br>males 51.16% females 48.84%<br>not stated/49.82	not stated	burnout (B)	Maslach Burnout Inventory - Human Services Survey (MBI-HSS)	EE=25.58%; DP=44.19%;<br>PA=51.16%.
Beyhan et al., 2013[4]/ Turkey (upper-middle income)	not stated	anaesthesiologists (68.2% - specialists; 30.75% - residents)	88<br>males 36.4% females 63.6%<br>25-45+/not stated	not stated	burnout (B)	Maslach Burnout Inventory - Human Services Survey (MBI-HSS)	EE=15.57(6.11); DP=5.35(3.08); PA=9.26(3.41).

© 2020 Sabitova A et al. *JAMA Network Open.*

de Paiva, 2017[5]/ Brazil (upper-middle income)	convenience	mixed (physicians from different specialities) (50.6% had a specialist degree; nurses and nursing practitioners were also included)	81<br>males 56.8% females 43.2%<br>not stated	not stated	burnout (B)	Maslach Burnout Inventory - Human Services Survey (MBI-HSS)	EE=27.2%; DP=50.6%;<br>PA=58.02%.
Erdur et al., 2015[6]/ Turkey (upper-middle income)	census	emergency physicians (88.5% had more than 6 years of work experience)	174<br>males 79.9% females 20.1%<br>24-59/36.8±5.8	85%	burnout (B)	Maslach Burnout Inventory - Human Services Survey (MBI-HSS)	not stated
Garcia et al., 2014[7]/ Brazil (upper-middle income)	convenience	physicians from different specialities (physicians with board certification only)	70<br>males 21% females 79%<br>not stated/36.2± 8.4	90%	burnout (B)	Maslach Burnout Inventory - Human Services Survey (MBI-HSS)	EE=44.3%; DP=24.3%; PA=17.1%.
Jugale et al., 2016[8]/ India (lower-middle income)	random	dentists (registered and practicing dentists only)	68<br>not stated<br>not stated	58.60%	burnout (B)	6 items from Maslach Burnout Inventory - Human Services Survey (MBI-HSS)	Not polled for meta-analysis because a short version of MBI-HSS was used.<br>Mean scores were given for males (m)

© 2020 Sabitova A et al. *JAMA Network Open.*

							and females (f) separately.<br>m: EE=4.98(3.93); DP=0.29(0.99); PA=4.40(1.91);<br>f: EE=5.00(3.86);<br>DP=0.12(0.43); PA=0.12(0.43).
Lesic et al., 2009[9]/ Serbia (upper-middle income)	not stated	physicians from different specialities (30 orthopaedic surgeons: 66,67%-specialists; 33,33% - residents<br>38 GPs: 2,63% - residents; 50% GPs; 47.37% - specialists of general medicine)	68<br>orthopaedic surgeons: males 83,33%; females 16.67%<br>GPs: males 21.05%; females 78.95%<br>26-60 /41.8 25-59/42.2	not stated	burnout (B)	Maslach Burnout Inventory - Human Services Survey (MBI-HSS)	EE=34.4%; DP=20.6%;<br>PA=39.7%.
Liadova et al., 2017[10]/ Russia (upper-middle income)	not stated	emergency physicians (certified physicians only)	50<br>males 60% females 40%<br>not stated	not stated	burnout (B)	Maslach Burnout Inventory (MBI)	Not pooled for meta-analysis because results were not stated clearly.<br>78% showed clear signs of B; 10% showed a high risk of DP.

© 2020 Sabitova A et al. *JAMA Network Open.*

Lima et al., 2013[11]/ Brazil (upper-middle income)	not stated	physicians from different specialities (83% had a specialist degree)	158<br><br>males 16.5%<br>females 83.5%<br><br>not stated/37.5	92.40%	burnout (B)	Maslach Burnout Inventory - Human Services Survey (MBI-HSS)	EE=61.4%;<br>DP=36.7%;<br>PA=13.3%.
Mandengue et al., 2017[12]/ Cameroon (lower-middle income)	census	GPs (55.3% had seniority in profession from 2 to 4 years)	85<br><br>males 51.8%<br>females 48.2%<br><br>24-42/not stated	64%	burnout (B)	Maslach Burnout Inventory - Human Services Survey (MBI-HSS)	EE=11.8%;<br>DP=10.6%;<br>PS=30.06%.
Margaryan, 2011[13]/ Armenia (lower-middle income)	random	physicians from different specialities (50.8% graduated more than 20 years ago)	832<br><br>males 24.4%<br>females 75.6%<br><br>22 - 79/45.3±0.4	79.20%	burnout (B)	Maslach Burnout Inventory - Human Services Survey (MBI-HSS)	EE=23.1(5.9);<br>DP=10.2(3.3);<br>PA=33.9(5.2).
Milenovic et al., 2016[14]/ Serbia (upper-middle income)	census	Anaesthesiologists (all had a specialist degree)	205<br><br>males 29.3%<br>females 70.7%<br><br>34-64/48.2± 8.3	76.20%	burnout (B)	Maslach Burnout Inventory - Human Services Survey (MBI-HSS)	EE=52.7%;<br>DP=12.2%;<br>PA=28.8%.
Novais et al., 2016[15]/ Brazil (upper-middle income)	not stated	on-call surgeons (88.37% had a specialization in surgery)	43<br><br>males 95.35%<br>females 4.65%<br><br>not stated/43.9±8.35	not stated	burnout (B)	Maslach Burnout Inventory - Human Services Survey (MBI-HSS)	EE=20.98(5.64);<br>DP=8.46(2.27);<br>PA=35.40(6.62).

© 2020 Sabitova A et al. *JAMA Network Open.*

Hamama et al., 2016[16]/ `-centred	not stated	mixed (HIV/AIDS physicians) (66% had a second and third academic degree)	319<br>males 13%<br>females 87%<br>not stated/39.85±9.16 (Russia)<br>not stated/40.13±9.73 (Kazakhstan)<br>not stated/46.41±9.67 (Israel)	90%	burnout (B)	Maslach Burnout Inventory - Human Services Survey (MBI-HSS)	Not polled for meta-analysis because results were not stated clearly.<br>Mean scores were given by countries. SDs were not stated.<br>B(Russia)=1.65; B(Kazakhstan)=1.88; B(Israel)=1.31.
He et al., 2017[17]/ China(upper-middle income)	not stated	dual and single role oncologists (certified oncologists only)	299<br>Gender<br>dual role:<br>males 29.8%; females 70.2%<br>single role:<br>males 31.5%; females 68.5%<br>20 – 60/not stated	59% for dual role; 38% for single role	burnout (B)	Maslach Burnout Inventory - Human Services Survey (MBI-HSS)	EE=22%; DP=27%; PA=not given.
Palmer-Morales et al., 2005[18,19] / Mexico (upper-middle income)	not stated	anaesthesiologists (practicing anaesthesiologists with at least 2 years of seniority)	89<br>males 72.8%<br>females 27.2%<br>31-56/43.7±7.2; 31-62/45.8±6.9	96.70%	burnout (B)	Maslach Burnout Inventory - Human Services Survey (MBI-HSS)	EE=17%; DP=12.3%; PA=10%.

© 2020 Sabitova A et al. *JAMA Network Open.*

Parfenov et al., 2012[20]/ Russia (upper-middle income)	not stated	physicians from different specialities (certified GPs, surgeons, dentists and physicians with managerial role)	504<br>Gender<br>Physicians with managerial role: males 34.6%; females 65.4%<br>GPs: males 57.8%; females 42.2%<br>surgeons: males 65.6%; females 34.4%<br>dentists: males 48.8%; females 51.2%<br>not stated/45.9	not stated	burnout (B)	Maslach Burnout Inventory (MBI)<br>Boiko's "emotional burnout" assessment method	Not pooled for meta-analysis because SDs were not stated.<br>EE=13.3; DP=11.2.
Popa et al., 2010[21]/ Romania (upper-middle income)	random	emergency physicians (7.4-mean years of work experience)	263<br>males 68% females 32%<br>not stated/39	69%	burnout (B)	Maslach Burnout Inventory (MBI)	Not pooled for meta-analysis because SDs were not stated.<br>EE=2.75
Porto et al., 2014[22]/ Brazil (upper-middle income)	not stated	oral and maxillofacial surgeons (64.66% had more than 6 years of work experience)	116<br>males 81.9% females 18.1%<br>30-41/not stated	23.20%	burnout (B)	Maslach Burnout Inventory - Human Services Survey (MBI-HSS)	EE=12.11(8.26); DP=2.16(3.54); PA=30.56(5.83).

© 2020 Sabitova A et al. *JAMA Network Open.*

Putnik and Houkes, 2011[23]/ Serbia (upper-middle income)	census	physicians from different specialities (19.8 - mean years of work experience for males; 19.6 for females)	373<br>males 16% females 84%<br>not stated/47±10.16 for men<br>not stated/47±8.48 for women	44%	burnout (B)	Maslach Burnout Inventory (MBI - HSS)	EE=48.26%; DP=12.87%; PA=5.09%.
Rui et al., 2016[24]/ China (upper-middle income)	random	anaesthesiologists (percentage of residents were below 40)	395<br>males 58.23% females 41.77%<br>23-64/31.2±8.4	94.90%	burnout (B)	Maslach Burnout Inventory (MBI - HSS)	EE=15.3%; DP=8.1%;<br>PA=34.6%.
Selmanovic et al., 2011[25]/ Bosnia and Herzegovina (upper-middle income)	census	physicians from different specialities (66% had a specialist degree, 34% - residents)	147<br>not stated<br>not stated	34,7%	burnout (B)	Maslach Burnout Inventory - Human Services Survey (MBI-HSS)	EE=10.15(9.14); DP=5.12(4.81); PA=38.11(11.39).
Stanetic and Tesanovic, 2013[26]/ Bosnia and Herzegovina (upper-middle income)	census	physicians from different specialities (40.2% had a specialist degree, 59.8% - GPs)	239<br>males 16.7% females 83.3%<br><35=32.6%; 36-45=28.9%; 46+=38.5%/not stated	50%	burnout (B)	Maslach Burnout Inventory (MBI-HSS)	EE=46%; DP=21.3%; PA=43.1%.

© 2020 Sabitova A et al. *JAMA Network Open.*

Tejada and Gomez, 2012[27]/ Colombia (upper-middle income)	not stated	Psychiatrists (non-graduates were excluded)	121<br>males 56.2% females 43.8%<br>27-82/40.94±9.9	not stated	burnout (B)	Questionnaire for the Evaluation of Burning Syndrome by the Work (CESQT)	Not pooled for meta-analysis because CESQT was used<br>B=9.9%
Zhou et al., 2016[28]/ China (upper-middle income)	random	physicians from different specialities (residents were excluded)	1129<br>males 41.82%. females 58.17%<br>not stated/38.04±7.74	88.60%	burnout (B)	Maslach Burnout Inventory – General Survey (MBI - GS)	not stated
Freire et al., 2016[29]/ Brazil (upper-middle income)	not stated	anaesthesiologists (68.1% had more than 14 years of work experience)	198<br>males 68.5% females 31.5%<br>27-56+/not stated	19.60%	burnout (B)	Maslach Burnout Inventory - Human Services Survey (MBI-HSS)	EE=26.9%; DP=41.3%; PA=37.2%.
Montenegro et al., 2017[30]/ Argentina (upper-middle income)	not stated	anaesthesiologists (52.2% had a specialist degree)	209<br>males 51% females 49%<br>not stated/52.2±10.0	not stated	burnout (B)	Maslach Burnout Inventory - Human Services Survey (MBI-HSS)	EE=31.6%; DP=46.9%; PA=14.4%.
Sánchez-Cruz and Mugártegui-Sánchez, 2013[31]/ Mexico (upper-middle income)	convenience	family physicians (physicians with less than 3 years of work experience were excluded)	130<br>males 52% females 48%<br>not stated/46 ± 2.5	77.80%	burnout (B)	Maslach Burnout Inventory (MBI)	Not pooled for meta-analysis because results were not stated clearly.<br>B=45% (59 cases).

© 2020 Sabitova A et al. *JAMA Network Open.*

Taycan et al., 2014[32]/ Turkey (upper-middle income)	census	physicians from different specialities (54.7% - GPs; 45.3% - specialists)	139<br><br>males 66.9%<br>females 33.1%<br><br>not stated/31.05± 4.84	71.20%	burnout (B)	Maslach Burnout Inventory - Human Services Survey (MBI-HSS)	EE=14.91(7.2);<br>DP=5.80(3.33);<br><br>PA=20.35(3.85).
Beltran et al., 2004[33]/ Mexico (upper-middle income)	random	family physicians (76.7% had a specialists degree)	163<br>males 63.8%<br>females 36.2%<br><br>not stated/47	not stated	burnout (B)	Maslach Burnout Inventory - Human Services Survey (MBI-HSS)	EE=16%;<br><br>DP=1.8%;<br><br>PA=84.7%.
Aftab et al., 2016[34]/ Pakistan (lower-middle income)	convenience	physicians from different specialities (60.85% graduated more than 7 years ago)	74<br><br>males 87.8%<br>females 12.2%<br><br>25-more than 55/not stated	not stated	burnout (B)	Abbreviated Maslach Burnout Inventory	Not pooled for meta-analysis, because Abbreviated MBI was used.<br><br>B=39.2%
Al-Dubai and Rampal, 2010[35]/ Yemen (lower-middle income)	census	physicians from different specialities (30% - specialists; 70% - GPs)	563<br><br>males 59.5%<br>females 40.5%<br><br>25 - 55/ 33.3±5.7	70.40%	burnout (B)	Maslach Burnout Inventory - Human Services Survey (MBI-HSS)	EE=31.1(13.0);<br>DP=7.2(6.7);<br>PA=35.7(10.1).
Peltzer et al., 2003[36]/ South Africa (upper-middle income)	random	physicians from different specialities (15.6 - mean years of work experience)	402<br><br>males 59.5%<br>females 40.5%<br><br>23-75/41.1±13.4	33.50%	burnout (B)	Maslach Burnout Inventory - Human Services Survey (MBI-HSS)	EE=24.2(10.8);<br><br>DP =11.4(6.7);<br><br>PA= 17.4(6.8).

© 2020 Sabitova A et al. *JAMA Network Open.*

Grau et al., 2009[37]/ multi-centred	convenience	mixed (physicians from different specialities) (85.4% - specialist physicians; 4% - nurses; 2.3% - dentists; 2.0% - psychologists; 1.3% - nutritionists; 5% - others)	11530<br>males 51% females 49%<br>not stated/41.7	not stated	burnout (B)	Maslach Burnout Inventory - Human Services Survey (MBI-HSS)	EE=24.55(12.6);<br>DP=7.6(6.15); PA=37.7(7.85).
Kosan et al., 2018[38]/ Turkey (upper-middle income)	census	physicians from different specialities (29.1% - GPs; 47.8% - physicians from internal medical sciences; 9.9% - physicians from basic medical sciences)	711<br>males 65% females 35%<br>not stated/34.4±7.7	97.40%	burnout (B)	Maslach Burnout Inventory - Human Services Survey (MBI-HSS)	EE= 15.6 (7.0);<br>DP= 5.7(3.9);<br>PA= 21.0(4.4).
Rajan and Engelbrecht, 2018[39]/ South Africa (upper-middle income)	census	emergency physicians (49.5% - medical officers; 38.7% - registrars; 7.5% - specialists; 4.3% - community service medical officers)	93<br>males 49% females 51%<br>20-59/not stated	80.60%	burnout (B)	Maslach Burnout Inventory - Human Services Survey (MBI-HSS)	EE= 31.69(10.32); DP=13.39(6.27);<br>PA= 34.87(6.54).
Salem et al., 2018[40]/ Lebanon	not stated	oncologists (76.5% - specialists; 23.5% - fellows)	51<br>males 68.6% females 31.4%	73%	burnout (B)	Maslach Burnout Inventory (MBI) - HSS	EE=33.3%; DP=13.7%; PA=19.6%.

© 2020 Sabitova A et al. *JAMA Network Open.*

(upper-middle income)			25 - more than 60/not stated				
Zheng et al., 2018[41]/ China (upper-middle income)	not stated	reconstructive surgeons (87.6% - had a specialist degree; 12.4% - residents)	202<br>males 100%<br>less than 30 – more than 60/not stated	12.30%	burnout (B)	Maslach Burnout Inventory (MBI) - HSS	EE=33.17(9.31); DP=13.55(5.51);<br>PA=not stated.
Huri et al., 2016[42]/ Turkey (upper-middle income)	not stated	dentists (63.20% - dentists; 36.79% - dentists with a doctoral degree)	337<br>males 51.92% females 48.07%<br>25-65/not stated	76%	burnout (B)	Maslach Burnout Inventory - Human Services Survey (MBI-HSS)	EE=38%;<br>DP=22.2%;<br>PA=12%.
Esquivel-Molina et al., 2007[43]/ Mexico (upper-middle income)	convenience	physicians from different specialities (all specialists: 66.5% - medical; 33.1% - surgical)	145<br>males 75.2% females 24.8%<br>not stated/41.9±6.12	87.40%	burnout (B)	Maslach Burnout Inventory - Human Services Survey (MBI-HSS)	EE=15.2%; DP=19.3%;<br>PA=4.1%.
Paiva et al., 2018[44]/ Brazil (upper-middle income)	census	mixed (61.2% - specialists; 38.8% - residents)	227<br>males 63% females 37%<br>not stated/34 (median)	70.50%	burnout (B)	Maslach Burnout Inventory - Human Services Survey (MBI-HSS)	EE=41.9%; DP=31.6%; PA=50.9%.
Xiao et al., 2014[45]/ China	not stated	emergency physicians (9.3 –	205	82%	burnout (B) job	Maslach Burnout	Not pooled for B meta-analysis

© 2020 Sabitova A et al. *JAMA Network Open.*

(upper-middle income)		mean years of experience from males; 8.7 – mean years of experience for females)	males 61% females 39%		satisfaction (JS)	Inventory – General Survey (MBI - GS) Minnesota Satisfaction Questionnaire (MSQ)	because short version of MBI-GS was used.<br><br>EE=6.98(5.79);<br>Cynism= 3.37(4.35);<br>PA=24.79(10.81);<br>JS=35.90%.
Zhou et al., 2017[46]/ China (upper-middle income)	not stated	mixed (neurologists: 69.2% - specialists; 30.8% - residents)	5590<br>males 46% females 54%<br>20-50/not stated	68.3% for directors 60.7% for neurologist s	burnout (B) job satisfaction (JS)	Maslach Burnout Inventory - Human Services Survey (MBI-HSS)<br>Consultants' Mental Health Questionnaire	EE=37.4%; DP=36.3%; PA=55.2%;<br>JS=74.3%.
Pu et al., 2017[47]/ China (upper-middle income)	not stated	neurologists (34.2% male-residents; 26.7% - female residents)	5558<br>males 46% females 54%<br>20-50/not stated	60.7% - responded, but 553 excluded - not complete	burnout (B) job satisfaction (JS)	Maslach Burnout Inventory (MBI) Questionnaire designed by Ramirez to measure job stress and satisfaction	Not pooled for B meta-analysis because medians and interquartile range were reported for males (m) and females (f) separately.<br><br>m: EE=17.31; DP=4.11; PA=25.39.

© 2020 Sabitova A et al. *JAMA Network Open.*

							f: EE=16.31; DP= 4.11; PA=25.39;<br><br>JS=74.5%
Ozyurt et al., 2006[48]/ Turkey (upper-middle income)	random	physicians from different specialities (32.9% - attending physicians; 18.6% - associate chief physicians; 5.1% - chief physicians; 32.1% - residents: 11.3% - medical assistants)	598<br><br>males 64% females 36%<br><br>less than 29-50/not stated	78%	burnout (B) job satisfaction (JS)	Maslach Burnout Inventory - Human Services Survey (MBI-HSS)<br><br>Minnesota Satisfaction Questionnaire (MSQ)	EE=13.46(6.18);<br><br>DP=4.3(3.35);<br><br>PA=22.60(4.18).<br><br>Not pooled for JS meta-analysis because mean scores were given for males (m) and females (f) separately.<br><br>m: JS summary mean score=70.84(11.86);<br><br>f: JS summary mean score=68.74(10.69)
Zhang and Feng, 2011[49]/ China (upper-middle income)	random	mixed (physicians from different specialities)	1451<br><br>males 66.2%. females 33.8%<br><br>less than 30 – more than 51/not stated	93.60%	job satisfaction (JS) burnout (B)	Chinese Physicians' Job Satisfaction Questionnaire (CPJSQ) Maslach Burnout Inventory – General Survey (MBI - GS)	JS=not stated;<br><br>B=not stated

© 2020 Sabitova A et al. *JAMA Network Open.*

Cetina-Tabares et al., 2006[50]/ Mexico (upper-middle income)	not stated	family physicians (practicing family physicians with at least 4 years of seniority)	93<br>males 53.8%<br>females 46.2%<br>not stated/44	94.70%	job satisfaction (JS)<br>burnout (B)	Font Roja-AP questionnaire<br>Maslach Burnout Inventory - Human Services Survey (MBI-HSS)	JS=86%;<br>Not pooled for B meta-analysis because results<br>were not stated clearly.<br>B(low)=50.5%;<br>B(moderate)=29%; B(high)=20.5%.
Sararaks et al., 1997[51]/ Malaysia (upper-middle income)	census	physicians from different specialities (70.4% - medical officers: 24.3% - specialists; 5.3% - house officers)	152<br>males 42.8%<br>females 57.2%<br>28.5-55/34.6±7.4	69.40%	job satisfaction (JS)	self-administered from 2 validated questionnaires	JS=31.3%
Cordoba et al., 2009[52]/ multi-centred	random	psychiatrists (52.7% had a sub-specialty)	1292<br>males 63.8%<br>females 36.2%<br>25-75/48.2±10.5	52.40%	job satisfaction (JS)	self-administered questionnaire	Not pooled for JS meta-analysis due to unclear sample size.<br>JS= 86.4%.
Wu et al., 2014[53]/ China (upper-middle income)	purposive	physicians from different specialities (58.4% had an undergraduate degree or higher)	202<br>males 51.98%<br>females 48.02%<br>not stated/35.2±7.6	81%	job satisfaction (JS)	self-administered questionnaire, based on 4 existing questionnaires	JS=not stated

© 2020 Sabitova A et al. *JAMA Network Open.*

Valderrama-Martinez and Davalos-Diaz, 2004[54]/ Mexico (upper-middle income)	random	family physicians (61.3% specialised in primary care)	160<br>males 58%<br>females 42%<br>21-51/not stated	88.90%	job satisfaction (JS)	self-administered questionnaire	JS=86%
Pau and Sabri, 2015[55]/ Malaysia (upper-middle income)	not stated	dentists (newly qualified dentists only)	342<br>males 23.4%<br>females 76.6%<br>not stated/27±1.7	58.90%	job satisfaction (JS)	Dentist Satisfaction Survey Questionnaire	Not pooled for meta-analysis because summary mean score was reported<br>JS summary mean score=145.48 (17.95)
Lu et al., 2017[56]/ China (upper-middle income)	random	physicians from different specialities (20.3% - chief physicians and associate-chief physicians; 30.6% - attending physicians; 39.5% - residents; 9.5% - no title)	3563<br>males 62.1%<br>females 37.9%<br>19 -more than 45/37.6±8.5	89.91%	job satisfaction (JS)	self-administered questionnaire (adapted from the Job Descriptive Index)	Not pooled for meta-analysis as six point Likert-scale was used<br>JS=3.93(1)
Ali Jadoo et al., 2015[57]/ Iraq (upper-middle income)	random	physicians from different specialities (39.8% - specialists; 60.2% - non-specialists physicians)	576<br>males 46.2%<br>females 53.8%<br>27-56/40.43±8.59	87.30%	job satisfaction (JS)	Warr-Cook-Wall Job Satisfaction Scale	Not pooled for meta-analysis because summary mean scoe was reported.

© 2020 Sabitova A et al. *JAMA Network Open.*

							JS summary mean score = 42.44(14.78)
Garcia-Pena et al., 2000[58]/ Mexico (upper-middle income)	random	physicians from different specialities (53.3% had a specialist degree)	213<br>physicians from the IMSS: males 61.7%; females 38.3%<br>physicians from the SSA: males 57.5%; females 42.5%<br>not stated/42.29; 40.15	not stated (non-response rate more than 10% in each group)	job satisfaction (JS)	self-administered questionnaire	JS=49%
O'Leary et al., 2009[59]/ Russia (upper-middle income)	convenience	physicians from different specialities (13.95 – mean years of experience)	203<br>males 28% females 72%<br>not stated/43.23±11.189 (hospitals); 48.21±10.042 (polyclinics)	67%	job satisfaction (JS)	Based on Physician Worklife Survey (PWS), but some new questions were developed	not stated
Omolase et al., 2010[60]/ Nigeria (lower-middle income)	random	ophthalmologists (60% - consultants; 35% - residents; 5% - diplomates)	80<br>males 42.5% females 57.5%<br>28-59-42.8±8	73%	job satisfaction (JS)	self-administered questionnaire	JS=78.5%
Ribeiro et al., 2014[61]/ Brazil	random	physicians from different specialities	232	87.20%	job satisfaction (JS)	self-administered questionnaire	JS=64.9%

© 2020 Sabitova A et al. *JAMA Network Open.*

(upper-middle income)		(fully qualified physicians only)	males 47% females 53%<br><br>not stated/41.4±9.9				
Smith, 1982[62]/ Thailand (upper-middle income)	purposive	physicians from different specialities (56% pursued post-graduate training)	127<br><br>males 75% females 25%<br><br>less than 35 – more than 55/not stated	62.30%	job satisfaction (JS)	self-administered questionnaire	JS=57%;
Yao et al., 2014[63]/ China (upper-middle income)	not stated	physicians from different specialities (11.16 – mean years of experience)	758<br><br>males 39.7%. females 60.3%<br><br>not stated/34.4±8.75	92.70%	job satisfaction (JS)	Quality of Working Life 7–32 Scale (QWL7–32 Scale)	Not pooled for meta-analysis because summary mean score was reported.<br><br>JS summary mean score= 24.15(6.29)
Zhang et al., 2013[64]/ China (upper-middle income)	purposive	physicians from different specialities (10.93 – mean years of experience)	207<br><br>males 36.2% females 63.8%<br><br>25-50/not stated	41%	job satisfaction (JS)	A three-item scale developed by Cammann, Fichman, Jenkins and Klesh	JS=3.62(0.84)
Ashraf et al., 2014[65]/ Pakistan (lower-middle income)	random	family physicians (fully qualified physicians only)	288<br><br>males 77.5% females 22.5%<br><br>26-72/37±9	24%	job satisfaction (JS)	self-administered questionnaire	JS=74%

© 2020 Sabitova A et al. *JAMA Network Open.*

Bhattacherjee et al., 2016[66]/ India (lower-middle income)	census	physicians from different specialities (82% had a postgraduate degree)	255<br>males 73.3%<br>females 26.7%<br>not stated/43.31±8.84	92.70%	job satisfaction (JS)	self-administered questionnaire specifically developed by Kumar and Khan	JS=59.6%
Cui et al., 2017[67]/ China (upper-middle income)	not stated	dentists (94.1% - finished a specialty training program)	170<br>males 37.6%<br>females 62.4%<br>20-45/33.17±5.81	47.40%	job satisfaction (JS)	Dentist Satisfaction Survey (CDSS)	JS=37.6%
Ghazaili et al., 2016[68]/ Malaysia (upper-middle income)	random	family physicians (6.6 – mean years of practice)	100<br>males 18%<br>females 82%<br>not stated/42.5	85.50%	job satisfaction (JS)	Warr-Cook-Wall Job Satisfaction Scale	JS=85%
Jabbari et al., 2014[69]/ Iran (upper-middle income)	not stated	family physicians (5.23 – mean years of practice)	238<br>males 50%<br>females 50%<br>not stated/32.92±5.04	64,5%	job satisfaction (JS)	self-administered questionnaire	JS=32.4%
Kaipa et al., 2015[70]/ India (lower-middle income)	convenience	dentists (fully qualified dentists only)	66<br>males 86.4%<br>females 13.6%<br>not stated/30.04±4.1	82.50%	job satisfaction (JS)	Dentists Satisfaction Survey (DSS)	JS=3.08(0.28)

© 2020 Sabitova A et al. *JAMA Network Open.*

Khan, 2011[71]/ Pakistan (lower-middle income)	census	anaesthesiologists (82.5% - specialists; 17.5% - trainees)	40<br>not stated<br>27-69/41.77±10.39	23%	job satisfaction (JS)	self-administered questionnaire	JS=50%
Khuwaja et al., 2004[72]/ Pakistan (lower-middle income)	convenience	physicians from different specialities (38% - consultants; 29% - family/general practitioners; 29% – trainees)	182<br>males 58% females 42%<br>more than 30 – 61%	70%	job satisfaction (JS)	self-administered questionnaire	JS=32%
Kisa and Kisa, 2006[73]/ Turkey (upper-middle income)	not stated	physicians from different specialities (60.1% - specialists; 39.9% - GPs)	168<br>males 52.4% females 47.6%<br>not stated/35.87±7.80	74.70%	job satisfaction (JS)	self-administered questionnaire	not stated
Luboga et al., 2011[74]/ Uganda (low-income)	random	mixed (physicians from different specialities) (10 – mean years of experience)	62<br>males 90% females 10%<br>26-70/36	not stated	job satisfaction (JS)	self-administered questionnaire	JS=37%
Cakir et al., 2018[75]/ Turkey (upper-middle income)	random	physicians from different specialities (74% - GPs; 26% - specialists)	258<br>males 83.7% females 16.3%<br>29-73/51.5±9.06	not stated	job satisfaction (JS)	Minnesota Satisfaction Questionnaire (MSQ)	JS=3.4(0.4)

© 2020 Sabitova A et al. *JAMA Network Open.*

Fierro-Arias et al., 2018[76]/ Mexico (upper-middle income)	not stated	dermatologists (fully qualified dermatologists only)	219<br>males 27.4% females 72.6%<br>28-79/45.68	not stated	job satisfaction (JS)	self-administered questionnaire	JS=98.5%
Barikani et al., 2013[77]/ Iran (upper-middle income)	random	GPs (75.3% had more than 5 years of work experience)	150<br>males 27.3% females 72.2%<br>less than 30 – more than 40/not stated	not stated	job satisfaction (JS) job motivation (JM)	self-administered questionnaire	JS=26.7%
Chew et al., 2013[78]/ Malaysia (upper-middle income)	census	physicians from different specialities (41.3% - family medicine specialists; 36.4% - medical officers)	149<br>males 14.8% females 85.2%<br>26-63/39.1±8.0	33.10%	job satisfaction (JS) job motivation (JM)	Warr-Cook-Wall Job Satisfaction Scale	Not pooled for JS meta-analysis because summary mean score was reported.<br>JS summary mean score=71.2(13.3);<br>JM summary score=37.7±4.2; JM mean score= 6.28(0.27).
de Oliveira Vasconcelos Filho, 2016[79]/ Brazil (upper-middle income)	random	physicians from different specialities (fully qualified physicians only)	141<br>males 63% females 37%<br>28-67-43.3±10.56	not stated	job satisfaction (JS) job motivation (JM)	Physician Worklife Survey (PWS)	JS=3.3(1.09);<br>JM=not stated

© 2020 Sabitova A et al. *JAMA Network Open.*

Mendes et al., 2013[80]/ Brazil (upper-middle income)	random	mixed (physicians from different specialities) (84.8% - physicians: 95.2% - specialists; 4.8% - residents; 15.2% - nurses)	126<br>males 57.1%; females 42.9%<br>25 – more than 45/not stated	not stated	job motivation (JM)	Scale of Values Relating to Work (EVT)	not stated

NB: Either prevalence rates (%) or mean scores (Mean(SD)) were reported for each outcome of interest.

Burnout dimensions: EE – emotional exhaustion, DP – depersonalization, PA – personal accomplishment.

GP – general practitioner.

© 2020 Sabitova A et al. *JAMA Network Open.*

eAppendix 4. Burnout

Meta-analysis of the Prevalence of Low Personal Accomplishment Among Physicians and Dentists in LMICs (Based on Results Provided as Dichotomous Data)

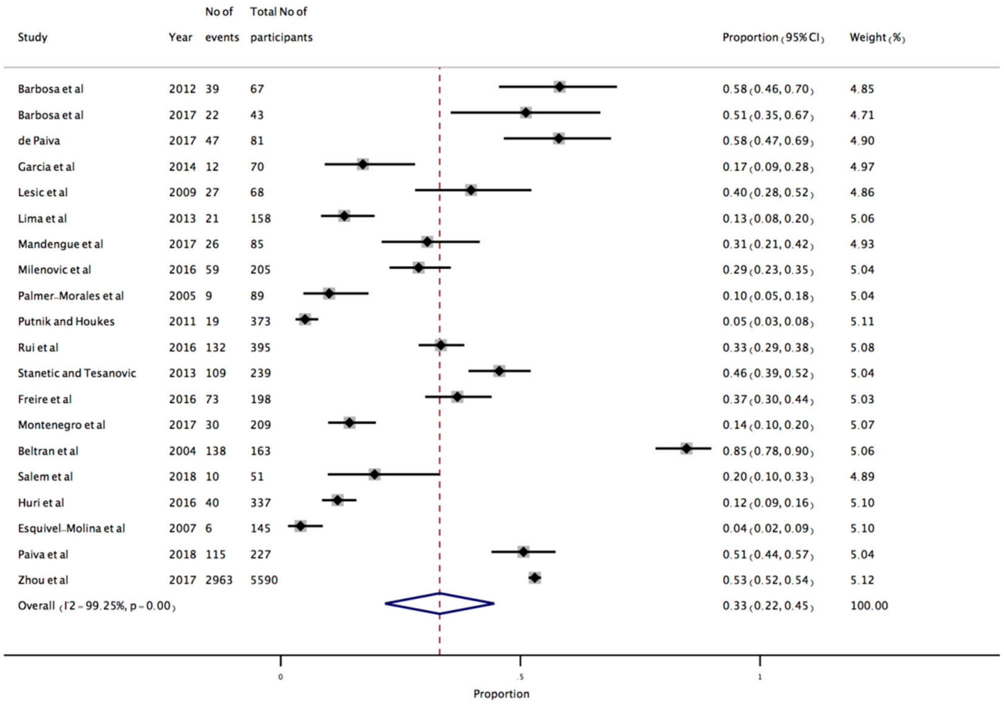

Meta-analysis of the Prevalence of High Emotional Exhaustion' Among Physicians in LMICs (Based on Results Provided as Dichotomous Data), Stratified by Geographical Region

NB: ES=Proportion

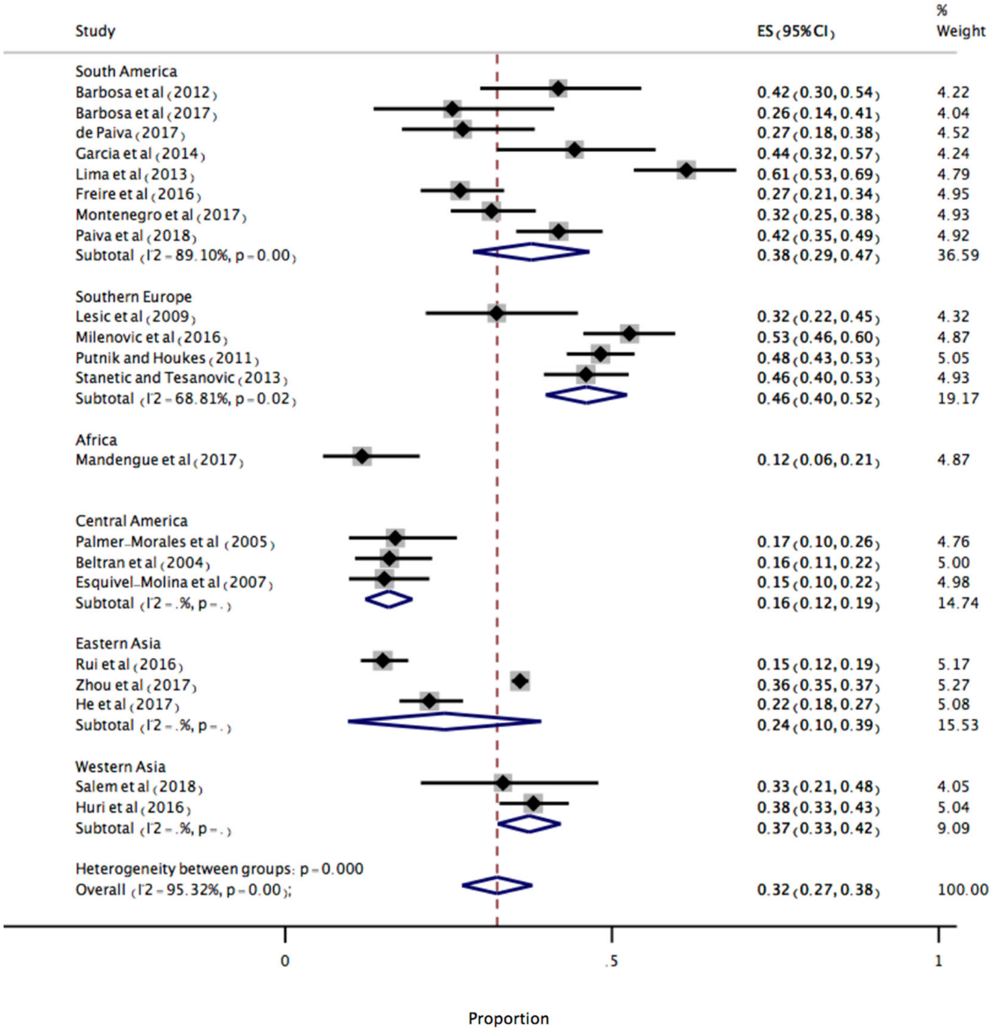

© 2020 Sabitova A et al. *JAMA Network Open.*

Metaregression of the Prevalence of High Emotional Exhaustion Among Physicians in LMICs (Based on Results Provided as Dichotomous Data), Stratified by Geographical Region

Meta-regression	Number of obs = 21
REML estimate of between-study variance	tau2 = .008496
% residual variation due to heterogeneity	I-squared_res = 93.10%
Proportion of between-study variance explained	Adj R-squared = 53.13%
Joint test for all covariates	Model F(5,15) = 4.83
With Knapp-Hartung modification	Prob > F = 0.0079

_ES	Coef.	Std. Err.	t	P>|t|	[95% Conf.	Interval]
Central America	.0420762	.1136235	0.37	0.716	-.2001066	.284259
Eastern Asia	.1269973	.1124798	1.13	0.277	-.1127477	.3667422
South America	.2592608	.1051273	2.47	0.026	.0351873	.4833343
Southern Europe	.3366872	.1104669	3.05	0.008	.1012327	.5721417
Western Asia	.2427873	.1227908	1.98	0.067	-.0189351	.5045098
_cons	.1176471	.0985762	1.19	0.251	-.0924631	.3277572

Meta-analysis of the Prevalence of High Depersonalization Among Physicians in LMICs (Based on Results Provided as Dichotomous Data), Stratified by Geographical Region

NB: ES=Proportion

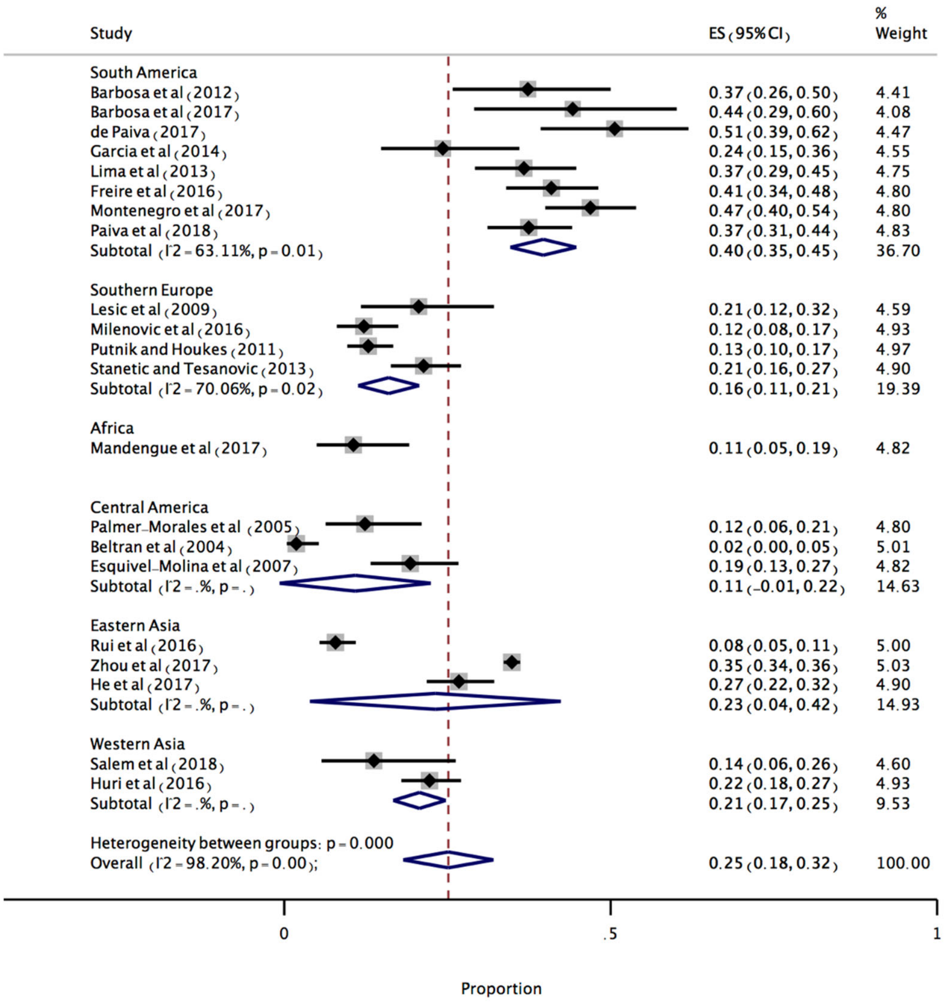

© 2020 Sabitova A et al. *JAMA Network Open.*

Metaregression of the Prevalence of High Depersonalization Among Physicians in LMICs (Based on Results Provided as Dichotomous Data), Stratified by Geographical Region

Meta-regression		Number of obs	= 21
REML estimate of between-study variance		tau2	= .006591
% residual variation due to heterogeneity		I-squared_res	= 96.16%
Proportion of between-study variance explained		Adj R-squared	= 64.06%
Joint test for all covariates		Model F(5,15)	= 7.11
With Knapp-Hartung modification		Prob > F	= 0.0014

_ES	Coef.	Std. Err.	t	P>|t|	[95% Conf. Interval]	
Central America	.0014572	.1007856	0.01	0.989	-.2133623	.2162767
Eastern Asia	.1257409	.099983	1.26	0.228	-.0873678	.3388496
South America	.2910616	.0938695	3.10	0.007	.0909836	.4911397
Southern Europe	.0588527	.097867	0.60	0.557	-.1497458	.2674512
Western Asia	.0788362	.1079746	0.73	0.477	-.1513063	.3089787
_cons	.1058824	.0877786	1.21	0.246	-.0812134	.2929781

© 2020 Sabitova A et al. *JAMA Network Open.*

Meta-analysis of the Prevalence of Low Personal Accomplishment Among Physicians in LMICs (Based on Results Provided as Dichotomous Data), Stratified by Geographical Region

NB: ES=Proportion

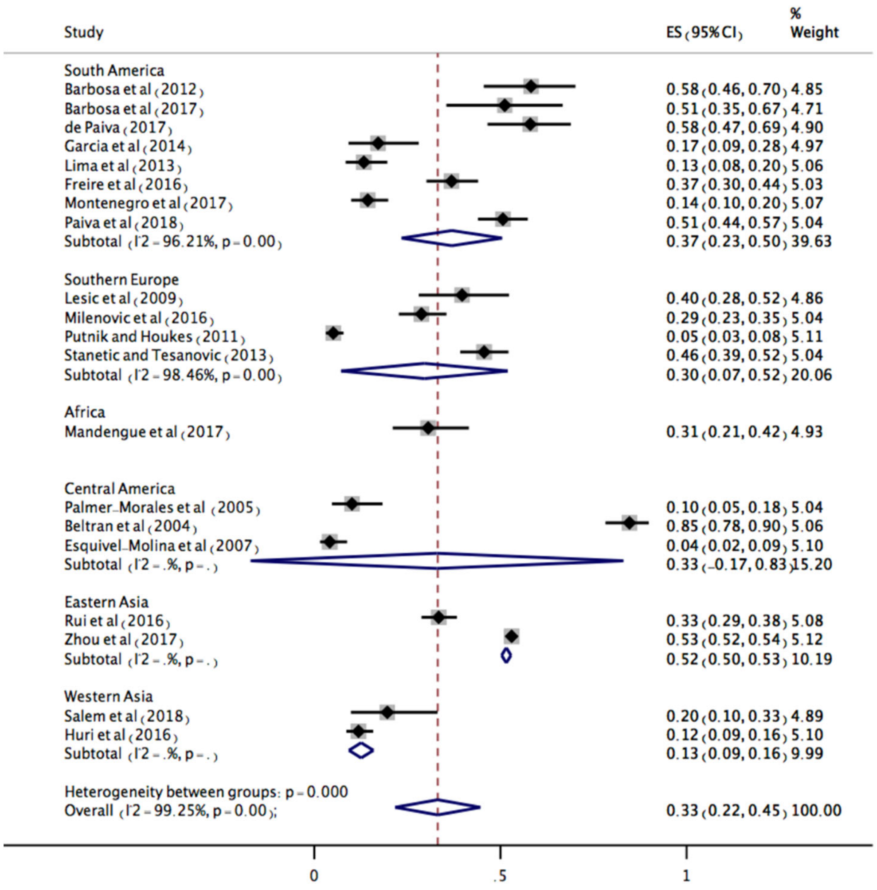

© 2020 Sabitova A et al. *JAMA Network Open.*

Metaregression of the Prevalence of Low Personal Accomplishment Among Physicians in LMICs (Based on Results Provided as Dichotomous Data), Stratified by Geographical Region.

_ES	Coef.	Std. Err.	t	P>|t|	[95% Conf. Interval]	
Central America	.0232366	.2785402	0.08	0.935	-.5741728	.620646
Eastern Asia	.1266831	.2947221	0.43	0.674	-.5054328	.7587991
South America	.0654168	.2567746	0.25	0.803	-.48531	.6161436
Southern Europe	-.0099572	.2702279	-0.04	0.971	-.5895384	.5696239
Western Asia	-.1494259	.2958772	-0.51	0.621	-.7840194	.4851676
_cons	.3058824	.2421551	1.26	0.227	-.2134887	.8252535

© 2020 Sabitova A et al. *JAMA Network Open.*

Meta-analysis of the Prevalence of High Emotional Exhaustion Among Physicians in LMICs (Based on Results Provided as Dichotomous Data), Stratified by Physicians' Specialities
NB: ES=Proportion

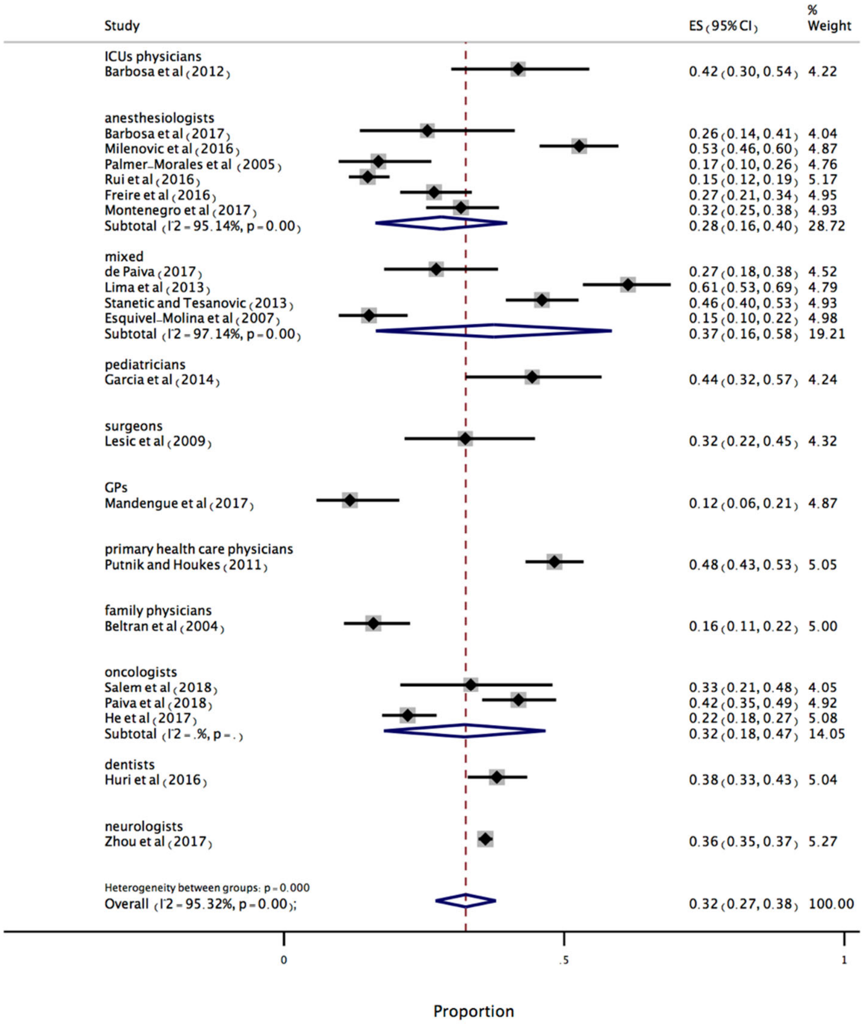

© 2020 Sabitova A et al. *JAMA Network Open.*

Metaregression of the Prevalence of High Emotional Exhaustion Among Physicians in LMICs (Based on Results Provided as Dichotomous Data), Stratified by Physicians' Specialities

Meta-regression	Number of obs = 21
REML estimate of between-study variance	tau2 = .02293
% residual variation due to heterogeneity	I-squared_res = 95.68%
Proportion of between-study variance explained	Adj R-squared = -26.49%
Joint test for all covariates	Model F(10,10) = 0.59
With Knapp-Hartung modification	Prob > F = 0.7910

_ES	Coef.	Std. Err.	t	P>|t|	[95% Conf. Interval]	
GPs	-.2058824	.224266	-0.92	0.380	-.7055781	.2938134
ICUs physicians	.094381	.2295752	0.41	0.690	-.4171443	.6059064
anaesthesiologists	-.0429189	.1738453	-0.25	0.810	-.4302702	.3444325
dentists	.0562925	.2230986	0.25	0.806	-.440802	.5533871
family physicians	-.1640202	.2233752	-0.73	0.480	-.6617312	.3336907
neurologists	.0358624	.2216194	0.16	0.875	-.4579365	.5296612
oncologists	-.0004723	.1855261	-0.00	0.998	-.4138503	.4129057
paediatricians	.1193277	.2293442	0.52	0.614	-.391683	.6303385
mixed	.0507821	.1795587	0.28	0.783	-.3492996	.4508638
primary health care physicians	.1590443	.2230323	0.71	0.492	-.3379027	.6559913
_cons	.3235294	.161698	2.00	0.073	-.0367562	.683815

© 2020 Sabitova A et al. *JAMA Network Open.*

Meta-analysis of the Prevalence of High Depersonalization Among Physicians in LMICs (Based on Results Provided as Dichotomous Data), Stratified by Physicians' Specialities
NB: ES=Proportion

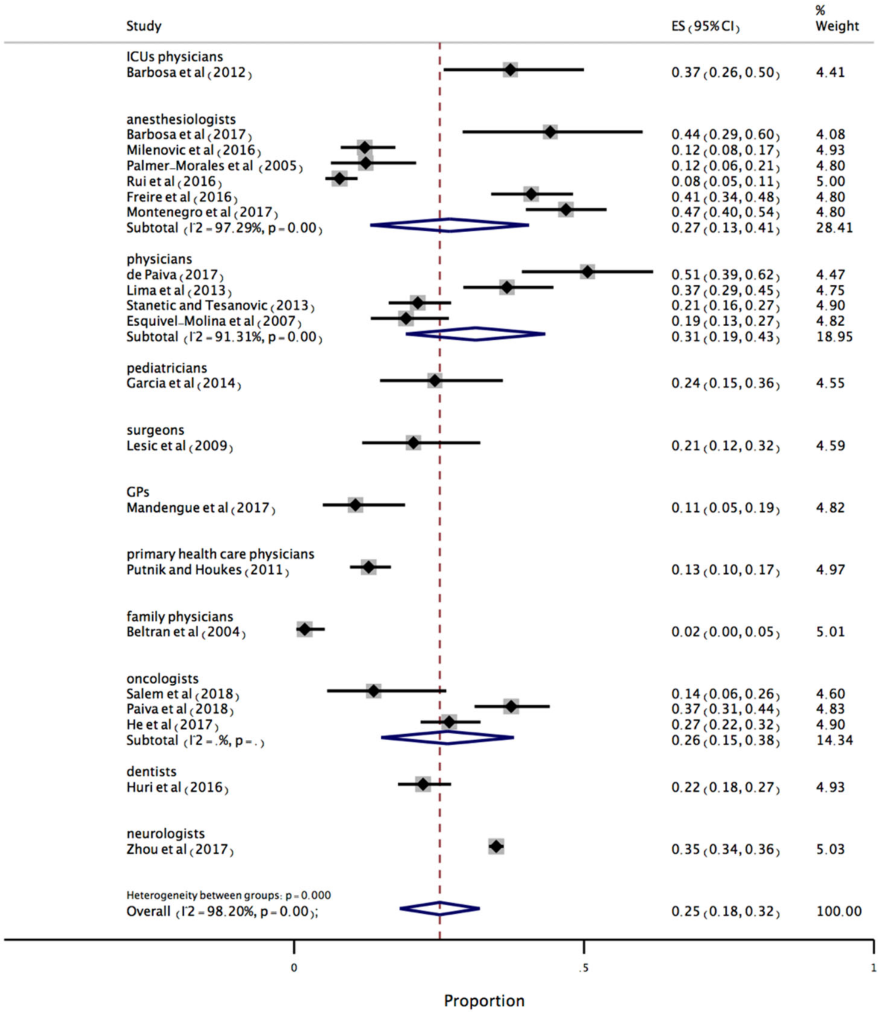

© 2020 Sabitova A et al. *JAMA Network Open.*

Metaregression of the Prevalence of High Depersonalization Among Physicians in LMICs (Based on Results Provided as Dichotomous Data), Stratified by Physicians' Specialities
NB: ES=Proportion

Meta-regression	Number of obs = 21
REML estimate of between-study variance	tau2 = .02428
% residual variation due to heterogeneity	I-squared_res = 95.78%
Proportion of between-study variance explained	Adj R-squared = -32.38%
Joint test for all covariates	Model F(10,10) = 0.54
With Knapp-Hartung modification	Prob > F = 0.8270

ES	Coef.	Std. Err.	t	P>|t|	[95% Conf. Interval]	
GPs	-.1	.2287092	-0.44	0.671	-.6095959	.4095959
ICUs physicians	.167252	.2338722	0.72	0.491	-.3538477	.6883517
anaesthesiologists	.0617425	.1764304	0.35	0.734	-.331369	.4548541
dentists	.0166696	.2273869	0.07	0.943	-.4899799	.5233191
family physicians	-.1874774	.2264958	-0.83	0.427	-.6921415	.3171866
neurologists	.142776	.22634	0.63	0.542	-.3615411	.647093
oncologists	.0559144	.1880747	0.30	0.772	-.3631422	.474971
paediatricians	.0369748	.2320084	0.16	0.877	-.4799722	.5539218
mixed	.1099799	.1824521	0.60	0.560	-.2965487	.5165084
primary health care physicians	-.077196	.2269163	-0.34	0.741	-.582797	.4284049
_cons	.2058824	.1637138	1.26	0.237	-.1588947	.5706594

© 2020 Sabitova A et al. *JAMA Network Open.*

Meta-analysis of the Prevalence of Low Personal Accomplishment Among Physicians in LMICs (Based on Results Provided as Dichotomous Data), Stratified by Physicians' Specialities

NB: ES=Proportion

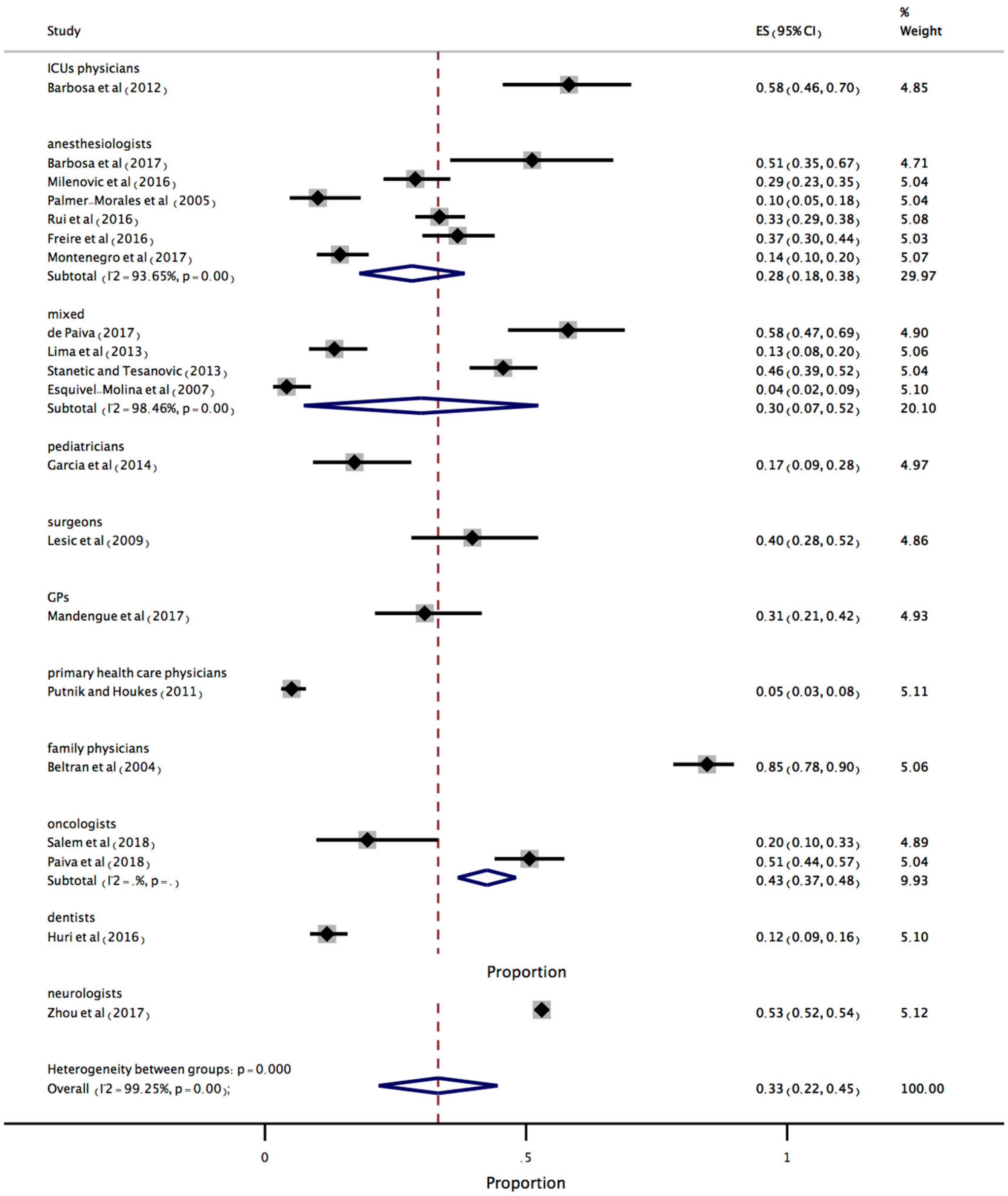

© 2020 Sabitova A et al. *JAMA Network Open.*

Metaregression of the Prevalence of Low Personal Accomplishment Among Physicians in LMICs (Based on Results Provided as Dichotomous Data), Stratified by Physicians' Specialities

Meta-regression Number of obs = 20
REML estimate of between-study variance tau2 = .03764
% residual variation due to heterogeneity I-squared_res = 96.97%
Proportion of between-study variance explained Adj R-squared = 18.71%
Joint test for all covariates Model F(10,9) = 1.38
With Knapp-Hartung modification Prob > F = 0.3185

ES	Coef.	Std. Err.	t	P>|t|	[95% Conf. Interval]	
GPs	-.0911765	.2865503	-0.32	0.758	-.7393982	.5570453
ICUs physicians	.1850307	.28854	0.64	0.537	-.4676921	.8377536
anaesthesiologists	-.1102592	.2194982	-0.50	0.627	-.6067986	.3862803
dentists	-.2783644	.2826688	-0.98	0.350	-.9178056	.3610767
family physicians	.4495669	.2835361	1.59	0.147	-.1918364	1.09097
neurologists	.1329949	.2821934	0.47	0.649	-.505371	.7713607
oncologists	-.0418282	.2482459	-0.17	0.870	-.6033993	.519743
paediatricians	-.2256302	.285723	-0.79	0.450	-.8719806	.4207201
mixed	-.0994533	.2266888	-0.44	0.671	-.6122591	.4133524
primary health care physicians	-.3461205	.2823456	-1.23	0.251	-.9848305	.2925896
_cons	.3970588	.2038923	1.95	0.083	-.0641775	.8582951

© 2020 Sabitova A et al. *JAMA Network Open.*

Meta-analysis of the Prevalence of High Emotional Exhaustion Among Physicians in LMICs (Based on Results Provided as Dichotomous Data), Stratified by Threshold

NB: ES=Proportion

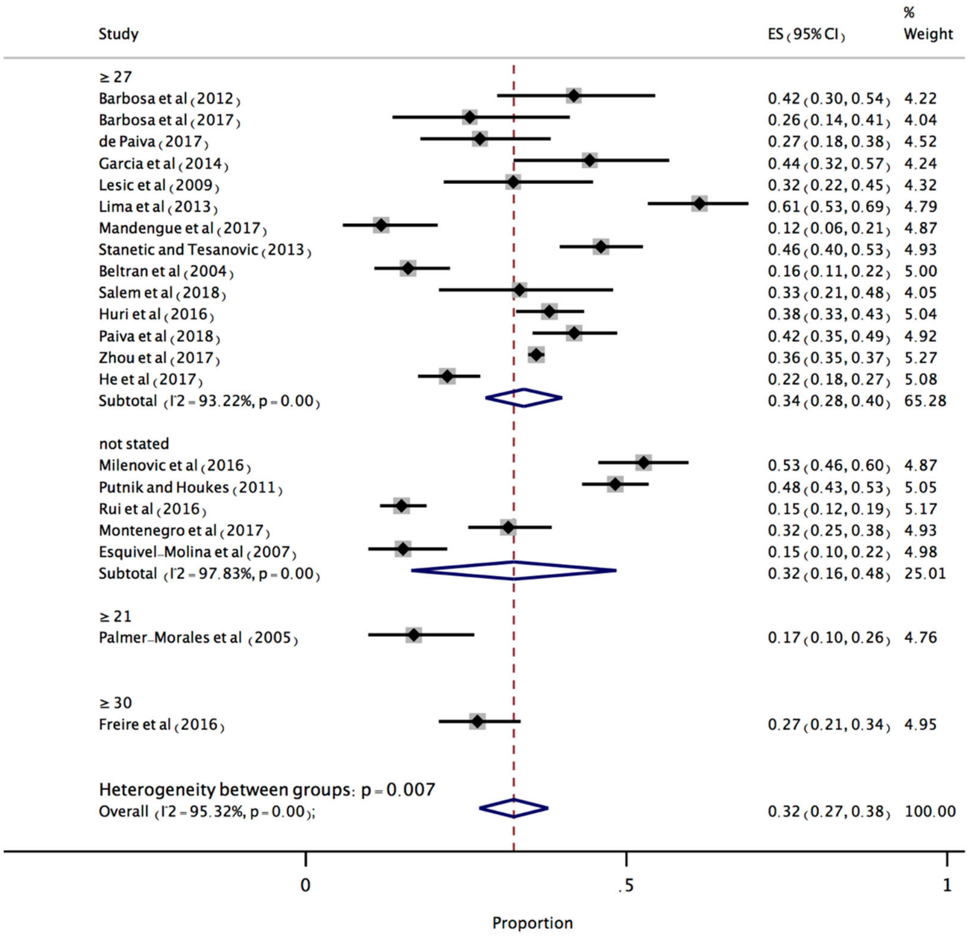

© 2020 Sabitova A et al. *JAMA Network Open.*

Metaregression of the Prevalence of High Emotional Exhaustion Among Physicians in LMICs (Based on Results Provided as Dichotomous Data), Stratified by Threshold

Meta-regression Number of obs = 21
REML estimate of between-study variance tau2 = .01978
% residual variation due to heterogeneity I-squared_res = 95.48%
Proportion of between-study variance explained Adj R-squared = -9.14%
Joint test for all covariates Model F(3,17) = 0.49
With Knapp-Hartung modification Prob > F = 0.6962

_ES	Coef.	Std. Err.	t	P>|t|	[95% Conf.	Interval]
≥ 21	-.1554497	.1596187	-0.97	0.344	-.4922158	.1813164
≥ 27	.0165109	.0753182	0.22	0.829	-.1423966	.1754185
≥ 30	-.0563123	.1577772	-0.36	0.726	-.389193	.2765684
_cons	.323989	.0641932	5.05	0.000	.1885532	.4594249

© 2020 Sabitova A et al. *JAMA Network Open.*

Meta-analysis of the Prevalence of High Depersonalization Among Physicians in LMICs (Based on Results Provided as Dichotomous Data), Stratified by Threshold

NB: ES=Proportion

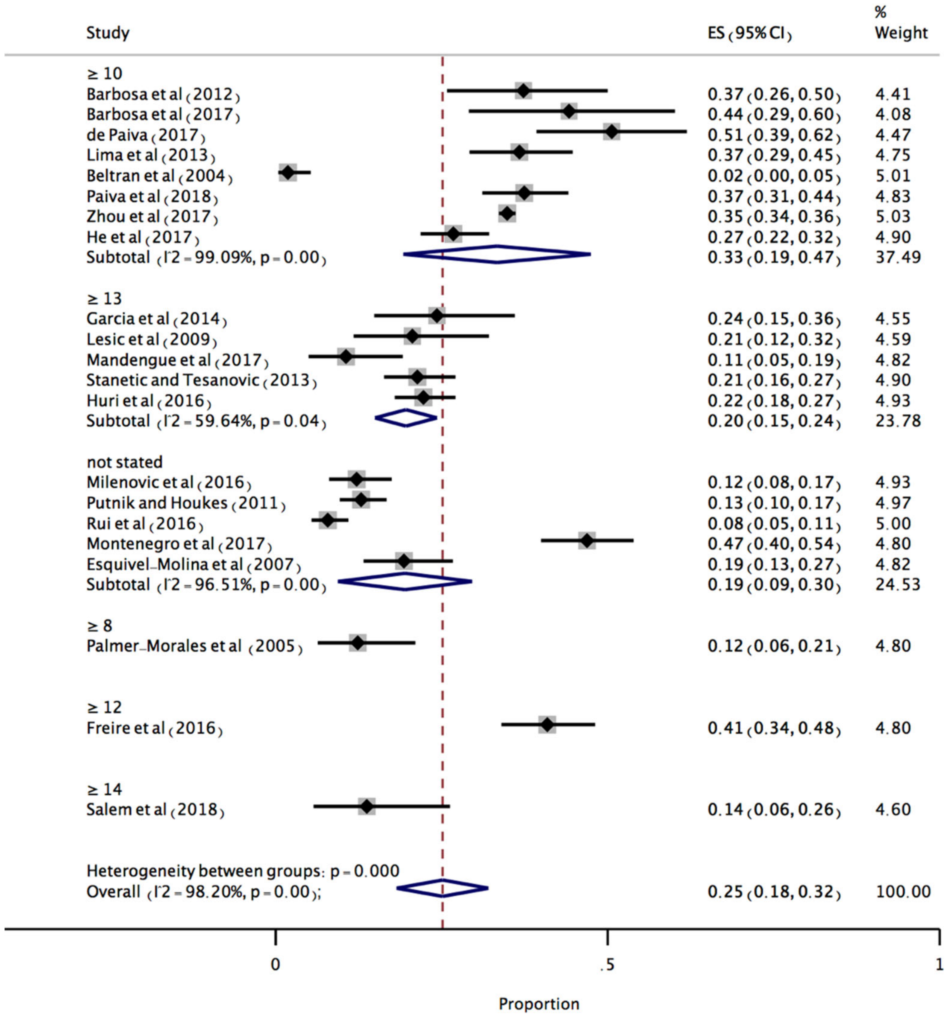

© 2020 Sabitova A et al. *JAMA Network Open.*

Metaregression of the Prevalence of High Depersonalization Among Physicians in LMICs (Based on Results Provided as Dichotomous Data), Stratified by Threshold

```
Meta-regression                                          Number of obs  =     21
REML estimate of between-study variance            tau2          =  .01665
% residual variation due to heterogeneity               I-squared_res  =  98.32%
Proportion of between-study variance explained      Adj R-squared  =   9.22%
Joint test for all covariates                           Model F(5,15)  =    1.46
With Knapp-Hartung modification                    Prob > F       =  0.2600
------------------------------------------------------------------------------
    _ES |     Coef.      Std. Err.      t      P>|t|     [95% Conf. Interval]
------------------------------------------------------------------------------
≥ 8     | -.0719461   .1460204    -0.49   0.629    -.3831811    .2392889
≥ 10    |  .1334082   .0759046     1.76   0.099    -.0283786    .2951951
≥ 12    |  .2135493   .1460334     1.46   0.164    -.0977135    .5248122
≥ 13    |  .0019966   .0841051     0.02   0.981    -.1772691    .1812624
≥ 14    | -.0582867   .1497556    -0.39   0.703    -.3774833    .2609099
_cons   |  .1955416   .0588073     3.33   0.005     .0701969    .3208863
```

© 2020 Sabitova A et al. *JAMA Network Open.*

Meta-analysis of the Prevalence Low Personal Accomplishment Among Physicians in LMICs (Based on Results Provided as Dichotomous Data), Stratified by Threshold

NB: ES=Proportion

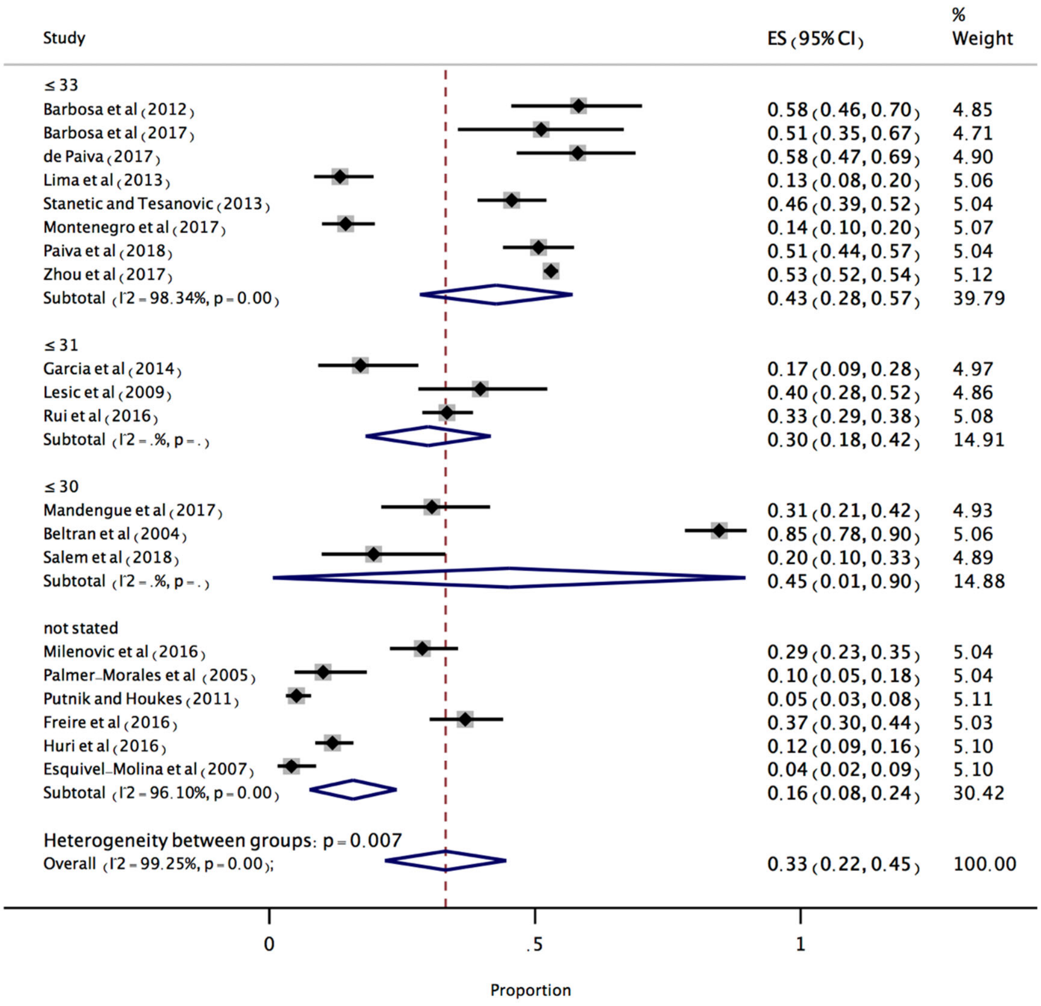

© 2020 Sabitova A et al. *JAMA Network Open.*

Metaregression of the Prevalence Low Personal Accomplishment Among Physicians in LMICs (Based on Results Provided as Dichotomous Data), Stratified by Threshold

```
Meta-regression                                      Number of obs  =      20
REML estimate of between-study variance           tau2          =  .03633
% residual variation due to heterogeneity            I-squared_res  =  97.79%
Proportion of between-study variance explained     Adj R-squared  =  21.54%
Joint test for all covariates                        Model F(3,16)  =    2.65
With Knapp-Hartung modification                   Prob > F       =  0.0842
------------------------------------------------------------------------------
    _ES |     Coef.   Std. Err.      t    P>|t|     [95% Conf. Interval]
------------------------------------------------------------------------------
≤ 30    |  .2964696   .1377381    2.15   0.047     .0044779    .5884613
≤ 31    |  .1398081   .1376124    1.02   0.325    -.1519171    .4315333
≤ 33    |  .2662436   .1046007    2.55   0.022     .0445001    .4879871
  _cons |  .1603217   .0784989    2.04   0.058    -.0060886     .326732
------------------------------------------------------------------------------
```

© 2020 Sabitova A et al. *JAMA Network Open.*

Meta-analysis of the Mean Score for Personal Accomplishment Among Physicians and Dentists in LMICs (Based on Results Provided as Continuous Data)

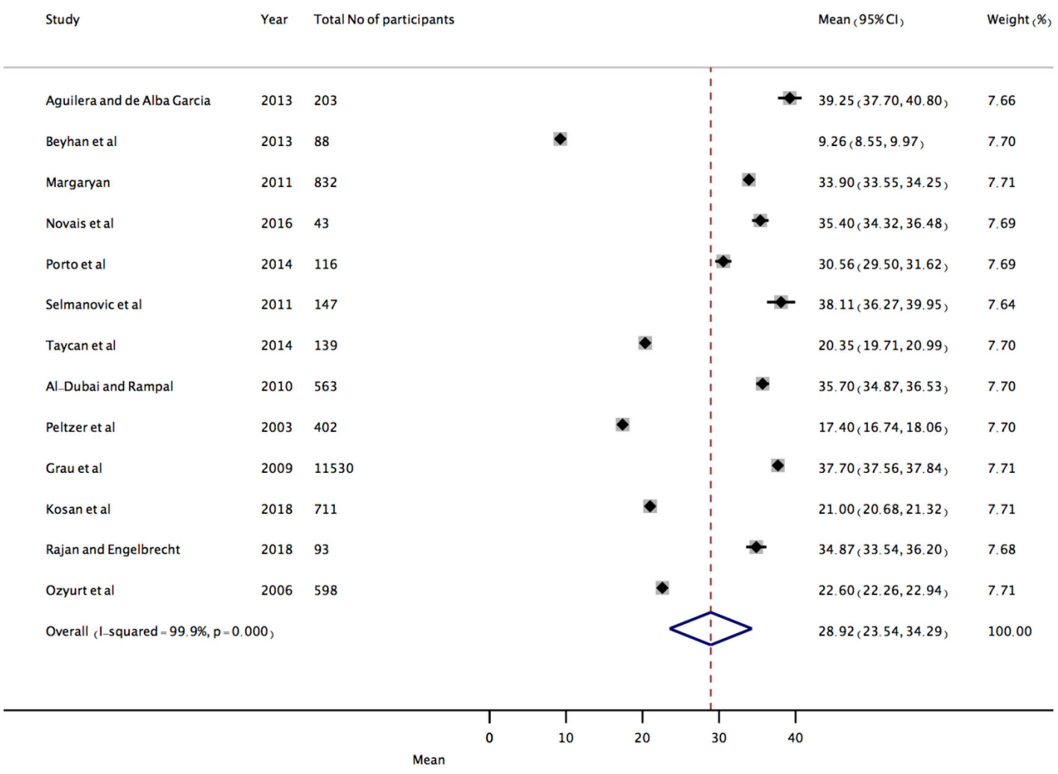

© 2020 Sabitova A et al. *JAMA Network Open.*

Meta-analysis of the Mean Score for Emotional Exhaustion (Based on Results Provided as Continuous Data), Stratified by Geographical Regions
NB: ES=Mean score

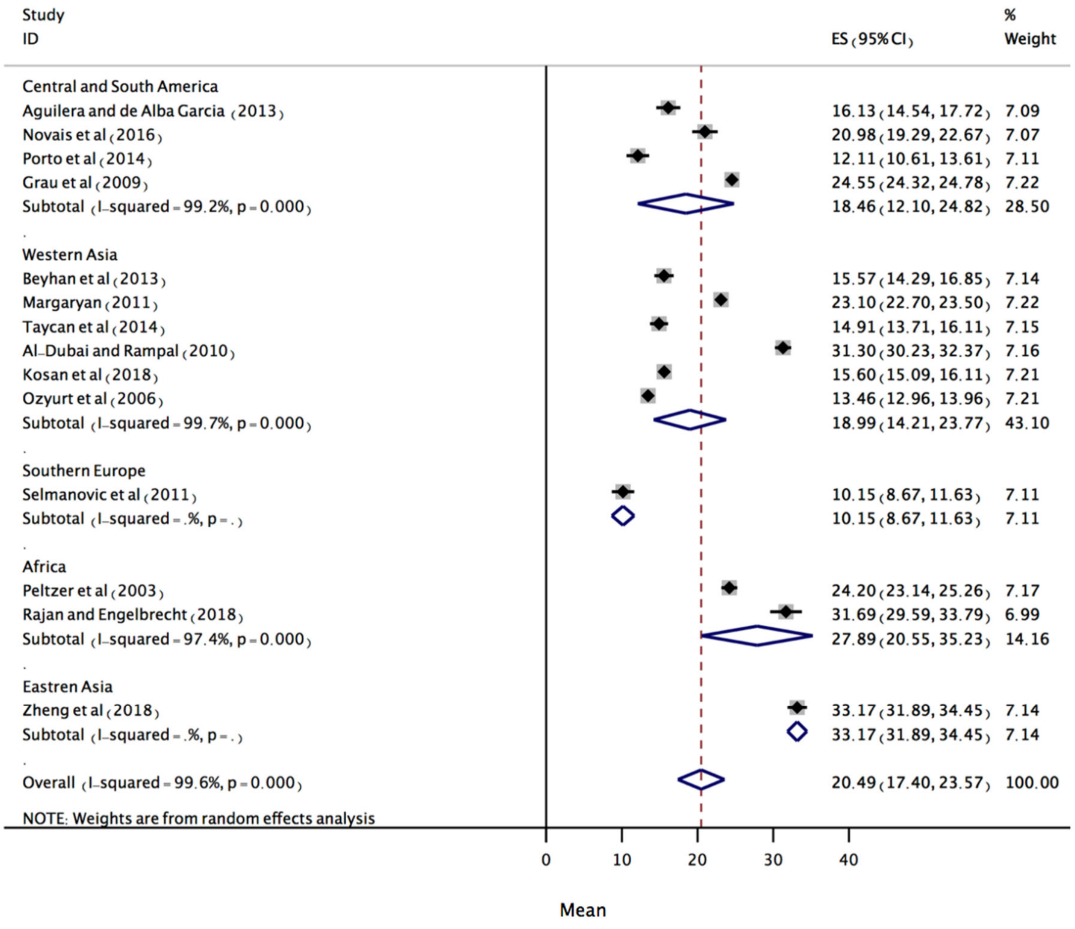

© 2020 Sabitova A et al. *JAMA Network Open.*

Metaregression of the Mean Score for Emotional Exhaustion (Based on Results Provided as Continuous Data), Stratified by Geographical Regions

Meta-regression	Number of obs = 14
REML estimate of between-study variance	tau2 = 39.3
% residual variation due to heterogeneity	I-squared_res = 99.56%
Proportion of between-study variance explained	Adj R-squared = 32.53%
Joint test for all covariates	Model F(4,9) = 2.56
With Knapp-Hartung modification	Prob > F = 0.1113

EEMean	Coef.	Std. Err.	t	P>|t|	[95% Conf. Interval]	
Africa	17.75499	7.737652	2.29	0.047	.2512037	35.25877
Central and South America	8.313342	7.057949	1.18	0.269	-7.652848	24.27953
Eastern Asia	23.02	8.921469	2.58	0.030	2.838236	43.20176
Western Asia	8.838676	6.815478	1.30	0.227	-6.579005	24.25636
_cons	10.15	6.313945	1.61	0.142	-4.133137	24.43314

© 2020 Sabitova A et al. *JAMA Network Open.*

Meta-analysis of the Mean Score for Depersonalization (Based on Results Provided as Continuous Data), Stratified by Geographical Regions

NB: ES=Mean score

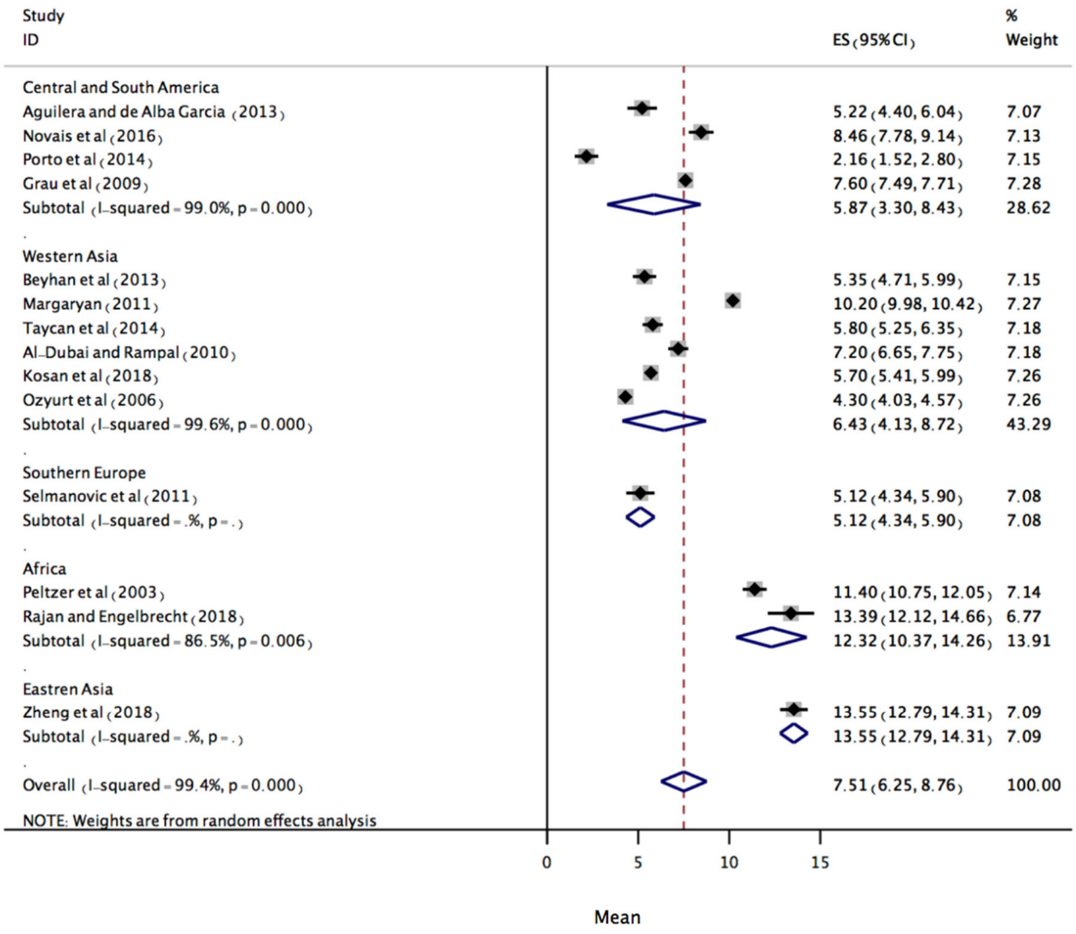

© 2020 Sabitova A et al. *JAMA Network Open.*

Metaregression of the Mean Score for Depersonalization (Based on Results Provided as Continuous Data), Stratified by Geographical Regions

Meta-regression	Number of obs = 14
REML estimate of between-study variance	tau2 = 5.207
% residual variation due to heterogeneity	I-squared_res = 99.44%
Proportion of between-study variance explained	Adj R-squared = 55.47%
Joint test for all covariates	Model F(4,9) = 4.97
With Knapp-Hartung modification	Prob > F = 0.0216

DPMean	Coef.	Std. Err.	t	P>|t|	[95% Conf. Interval]	
Africa	7.246732	2.845965	2.55	0.031	.8087108	13.68475
Central and South America	.7492304	2.586781	0.29	0.779	-5.102474	6.600935
Eastern Asia	8.43	3.274448	2.57	0.030	1.022684	15.83732
Western Asia	1.308482	2.498254	0.52	0.613	-4.342961	6.959925
_cons	5.12	2.31615	2.21	0.054	-.1194954	10.3595

© 2020 Sabitova A et al. *JAMA Network Open.*

Meta-analysis of the Mean Score for Personal Accomplishment (Based on Results Provided as Continuous Data), Stratified by Geographical Regions

NB: ES=Mean score

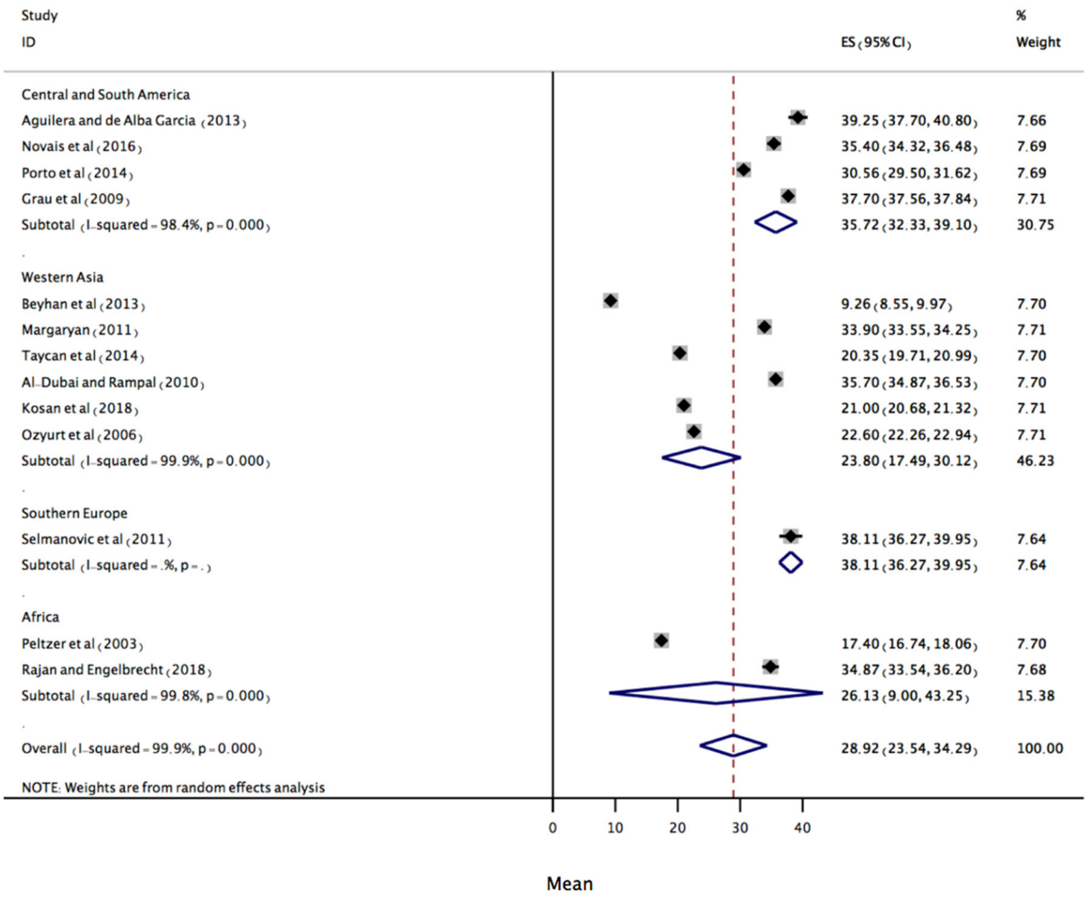

© 2020 Sabitova A et al. *JAMA Network Open.*

Metaregression of the Mean Score for Personal Accomplishment (Based on Results Provided as Continuous Data), Stratified by Geographical Regions

Meta-regression	Number of obs = 13
REML estimate of between-study variance	tau2 = 74.49
% residual variation due to heterogeneity	I-squared_res = 99.86%
Proportion of between-study variance explained	Adj R-squared = 19.61%
Joint test for all covariates	Model F(3,9) = 1.97
With Knapp-Hartung modification	Prob > F = 0.1888

PAMean	Coef.	Std. Err.	t	P>|t|	[95% Conf. Interval]	
Africa	-11.99514	10.6188	-1.13	0.288	-36.01654	12.02625
Central and South America	-2.384445	9.698952	-0.25	0.811	-24.325	19.55611
Western Asia	-14.30852	9.370165	-1.53	0.161	-35.5053	6.888269
_cons	38.11	8.681653	4.39	0.002	18.47074	57.74926

© 2020 Sabitova A et al. *JAMA Network Open.*

Meta-analysis of the Mean Score for Emotional Exhaustion (Based on Results Provided as Continuous Data), Stratified by Physicians' Specialities
NB: ES=Mean score

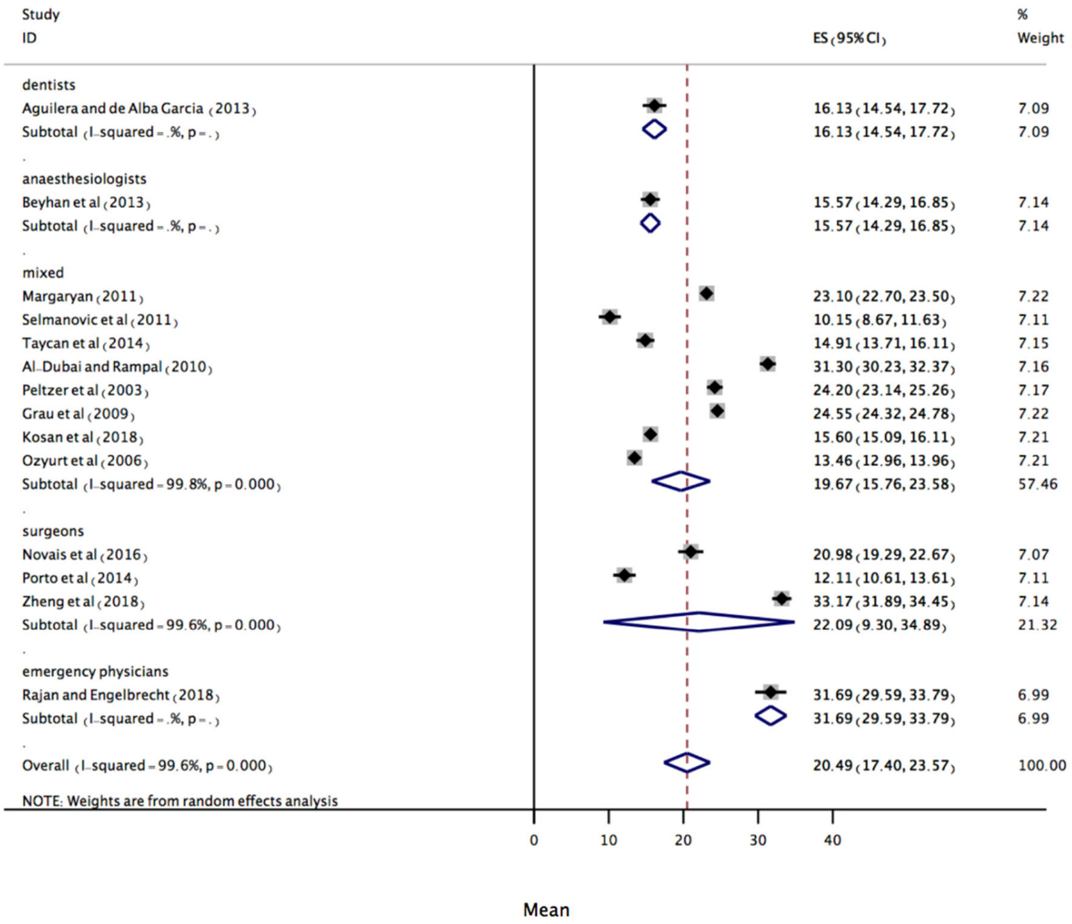

© 2020 Sabitova A et al. *JAMA Network Open.*

Metaregression of Mean Score for Emotional Exhaustion (Based on Results Provided as Continuous Data), Stratified by Physicians' Specialities

Meta-regression	Number of obs = 14
REML estimate of between-study variance	tau2 = 64.35
% residual variation due to heterogeneity	I-squared_res = 99.73%
Proportion of between-study variance explained	Adj R-squared = -10.49%
Joint test for all covariates	Model F(4,9) = 0.70
With Knapp-Hartung modification	Prob > F = 0.6139

EEMean	Coef.	Std. Err.	t	P>|t|	[95% Conf. Interval]	
anaesthesiologists	-4.09419	8.540665	-0.48	0.643	-23.41452	15.22614
dentists	-3.53419	8.554242	-0.41	0.689	-22.88523	15.81685
emergency physicians	12.02581	8.582824	1.40	0.195	-7.389886	31.44151
surgeons	2.432408	5.454883	0.45	0.666	-9.907394	14.77221
_cons	19.66419	2.842786	6.92	0.000	13.23336	26.09502

© 2020 Sabitova A et al. *JAMA Network Open.*

Meta-analysis of the Mean Score for Depersonalization (Based on Results Provided as Continuous Data), Stratified by Physicians' Specialities

NB: ES=Mean score

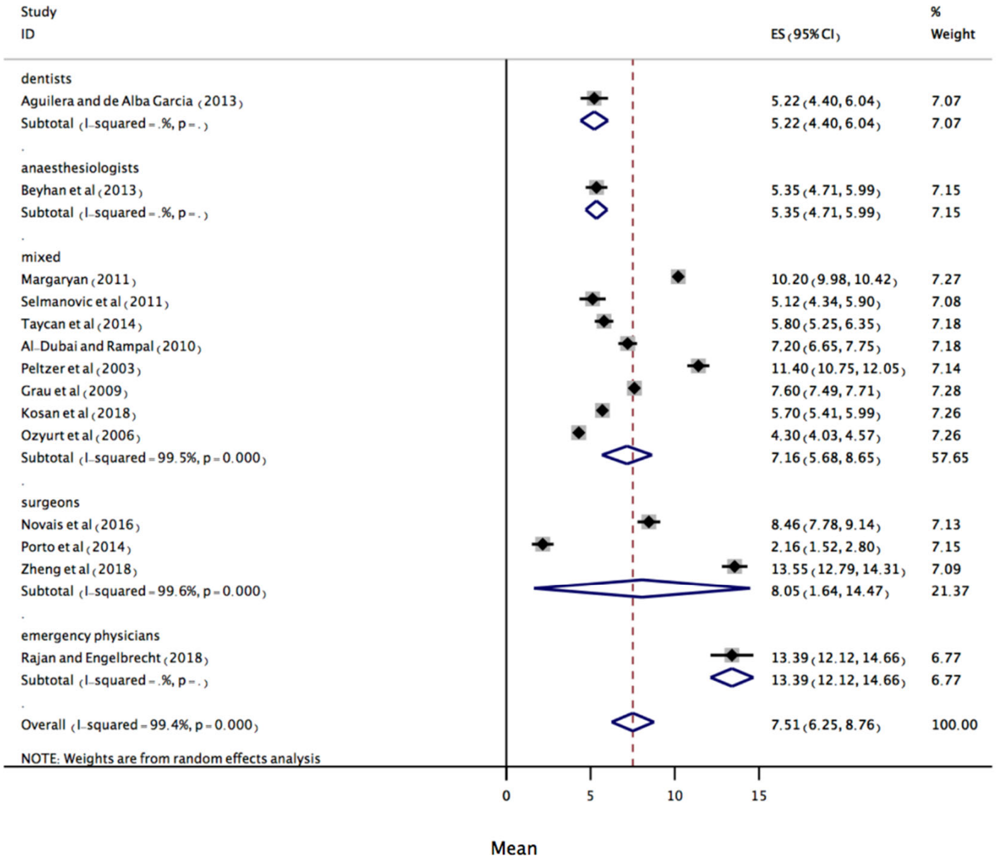

© 2020 Sabitova A et al. *JAMA Network Open.*

Metaregression of the Mean Score for Depersonalization (Based on Results Provided as Continuous Data), Stratified by Physicians' Specialities

Meta-regression	Number of obs	=	14
REML estimate of between-study variance	tau2	=	11.96
% residual variation due to heterogeneity	I-squared_res	=	99.55%
Proportion of between-study variance explained	Adj R-squared	=	-2.28%
Joint test for all covariates	Model F(4,9)	=	0.94
With Knapp-Hartung modification	Prob > F	=	0.4843

DPMean	Coef.	Std. Err.	t	P>|t|	[95% Conf. Interval]	
anaesthesiologists	-1.814983	3.688238	-0.49	0.634	-10.15836	6.528391
dentists	-1.944983	3.697119	-0.53	0.612	-10.30845	6.418481
emergency physicians	6.225017	3.730791	1.67	0.130	-2.214618	14.66465
surgeons	.885151	2.354692	0.38	0.716	-4.441533	6.211835
_cons	7.164983	1.227264	5.84	0.000	4.388719	9.941248

© 2020 Sabitova A et al. *JAMA Network Open.*

Meta-analysis of the Mean Score for Personal Accomplishment Based on Results Provided as Continuous Data), Stratified by Physicians' Specialities
NB: ES=Mean score

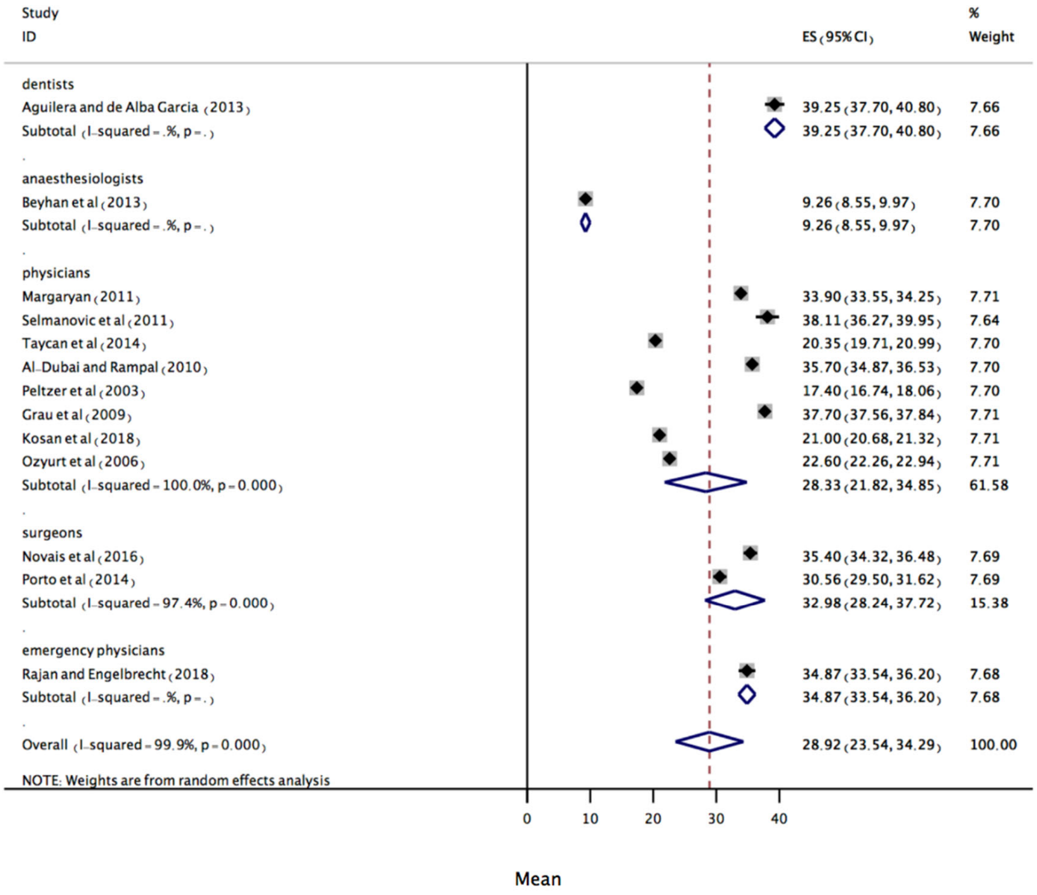

© 2020 Sabitova A et al. *JAMA Network Open.*

Metaregression of the Mean Score for Personal Accomplishment Based on Results Provided as Continuous Data), Stratified by Physicians' Specialities

NB: ES=Mean score

Meta-regression	Number of obs = 13
REML estimate of between-study variance	tau2 = 68.52
% residual variation due to heterogeneity	I-squared_res = 99.95%
Proportion of between-study variance explained	Adj R-squared = 26.04%
Joint test for all covariates	Model F(4,8) = 2.05
With Knapp-Hartung modification	Prob > F = 0.1798

PAMean	Coef.	Std. Err.	t	P>|t|	[95% Conf.	Interval]
anaesthesiologists	-19.07112	8.790796	-2.17	0.062	-39.34273	1.200496
dentists	10.91888	8.818647	1.24	0.251	-9.416953	31.25472
emergency physicians	6.538884	8.809428	0.74	0.479	-13.77569	26.85346
surgeons	4.648678	6.558801	0.71	0.499	-10.47594	19.7733
_cons	28.33112	2.931009	9.67	0.000	21.5722	35.09004

© 2020 Sabitova A et al. *JAMA Network Open.*

Meta-analysis of the Mean Score for Emotional Exhaustion (Based on Results Provided as Continuous Data), Stratified by Physicians' Specialities

NB: ES=Mean score

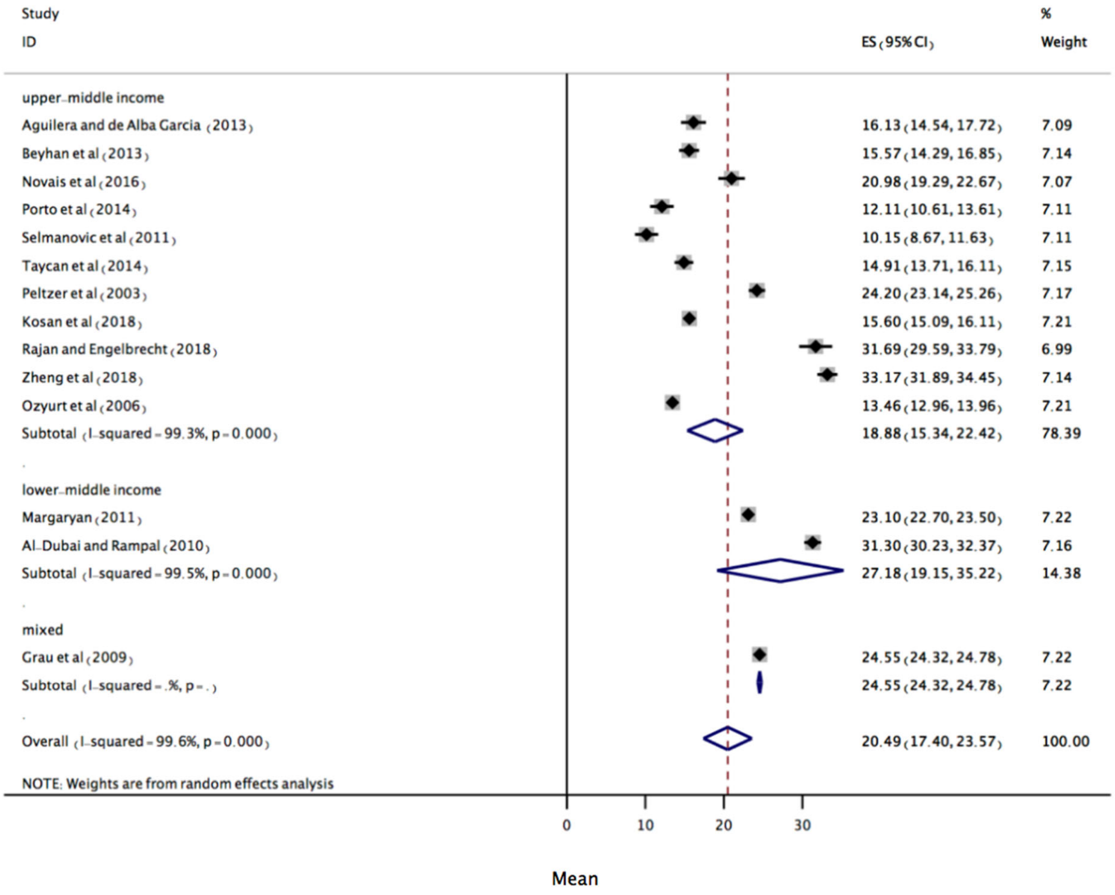

© 2020 Sabitova A et al. *JAMA Network Open.*

Metaregression of the Mean Score for Emotional Exhaustion (Based on Results Provided as Continuous Data), Stratified by Physicians' Specialities

Meta-regression Number of obs = 14
REML estimate of between-study variance tau2 = 56.63
% residual variation due to heterogeneity I-squared_res = 99.29%
Proportion of between-study variance explained Adj R-squared = 2.78%
Joint test for all covariates Model F(2,11) = 1.18
With Knapp-Hartung modification Prob > F = 0.3436

EEMean	Coef.	Std. Err.	t	P>|t|	[95% Conf.	Interval]
lower-middle-income	2.640676	9.233216	0.29	0.780	-17.6815	22.96285
upper-middle income	-5.65873	7.873204	-0.72	0.487	-22.98754	11.67008
_cons	24.55	7.53541	3.26	0.008	7.964674	41.13533

© 2020 Sabitova A et al. *JAMA Network Open.*

Meta-analysis of the Mean Score for Depersonalization (Based on Results Provided as Continuous Data), Stratified by Country's Income Group.
NB: ES=Mean score

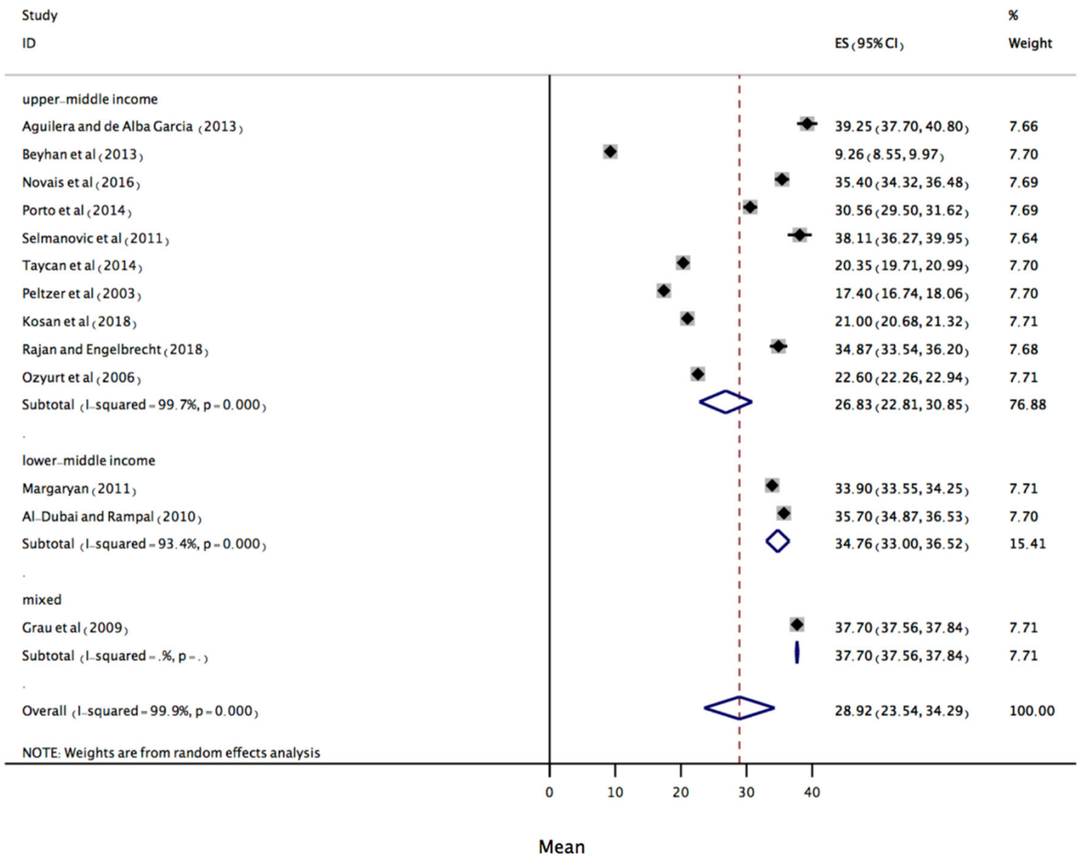

© 2020 Sabitova A et al. *JAMA Network Open.*

Metaregression of the Mean Score for Depersonalization (Based on Results Provided as Continuous Data), Stratified by Country's Income Group

Meta-regression	Number of obs = 13
REML estimate of between-study variance	tau2 = 92.25
% residual variation due to heterogeneity	I-squared_res = 99.71%
Proportion of between-study variance explained	Adj R-squared = 0.44%
Joint test for all covariates	Model F(2,10) = 1.02
With Knapp-Hartung modification	Prob > F = 0.3949

PAMean	Coef.	Std. Err.	t	P>|t|	[95% Conf.	Interval]
lower-middle income	-2.900724	11.76989	-0.25	0.810	-29.12568	23.32423
upper-middle income	-10.84217	10.07873	-1.08	0.307	-33.29899	11.61465
_cons	37.7	9.608317	3.92	0.003	16.29134	59.10866

© 2020 Sabitova A et al. *JAMA Network Open.*

Meta-analysis of the Mean Score for Personal Accomplishment (Based on Results Provided as Continuous Data), Stratified by Country's Income Group
NB: ES=Mean score

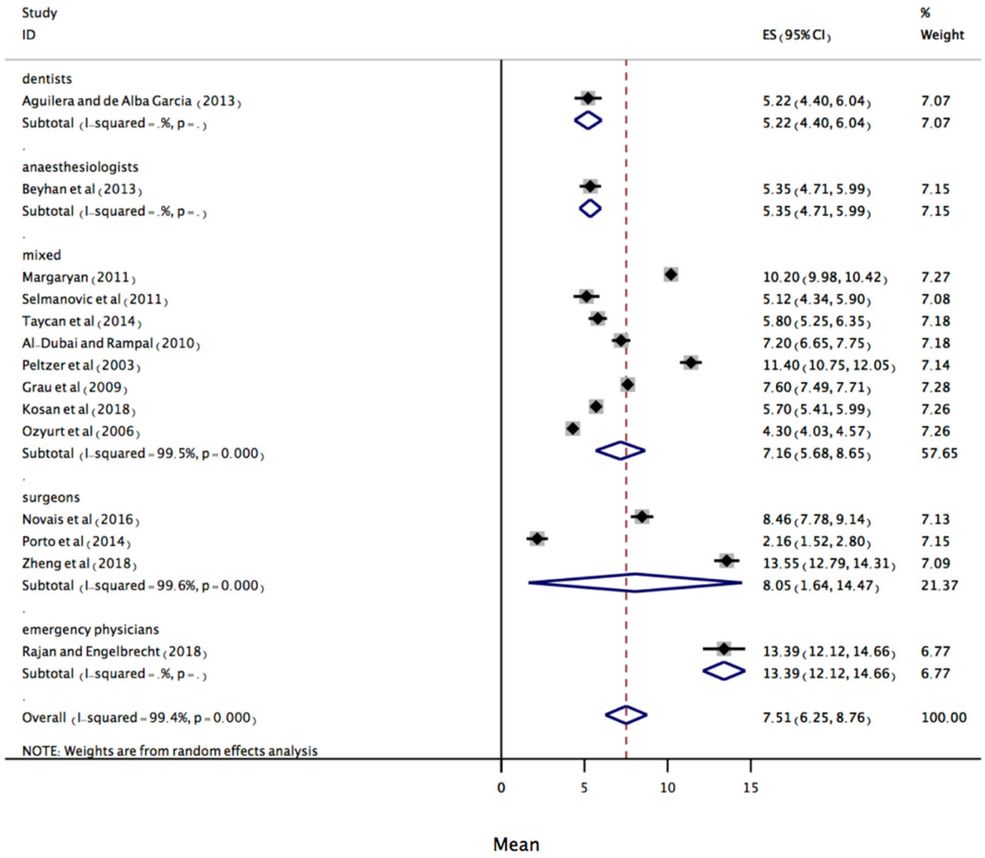

© 2020 Sabitova A et al. *JAMA Network Open.*

Metaregression of the Mean Score for Personal Accomplishment (Based on Results Provided as Continuous Data), Stratified by Country's Income Group

Meta-regression	Number of obs = 14
REML estimate of between-study variance	tau2 = 13.56
% residual variation due to heterogeneity	I-squared_res = 99.10%
Proportion of between-study variance explained	Adj R-squared = -15.95%
Joint test for all covariates	Model F(2,11) = 0.12
With Knapp-Hartung modification	Prob > F = 0.8859

DPMean	Coef.	Std. Err.	t	P>|t|	[95% Conf.	Interval]
lower-middle income	1.103674	4.522974	0.24	0.812	-8.851325	11.05867
upper-middle income	-.3021667	3.856702	-0.08	0.939	-8.79071	8.186377
_cons	7.6	3.691041	2.06	0.064	-.5239257	15.72393

© 2020 Sabitova A et al. *JAMA Network Open.*

Sensitivity Analysis of Emotional Exhaustion Studies (Based on Results Provided as Dichotomous Data), Exclusion of Studies with Lower Quality
NB: ES=Proportion

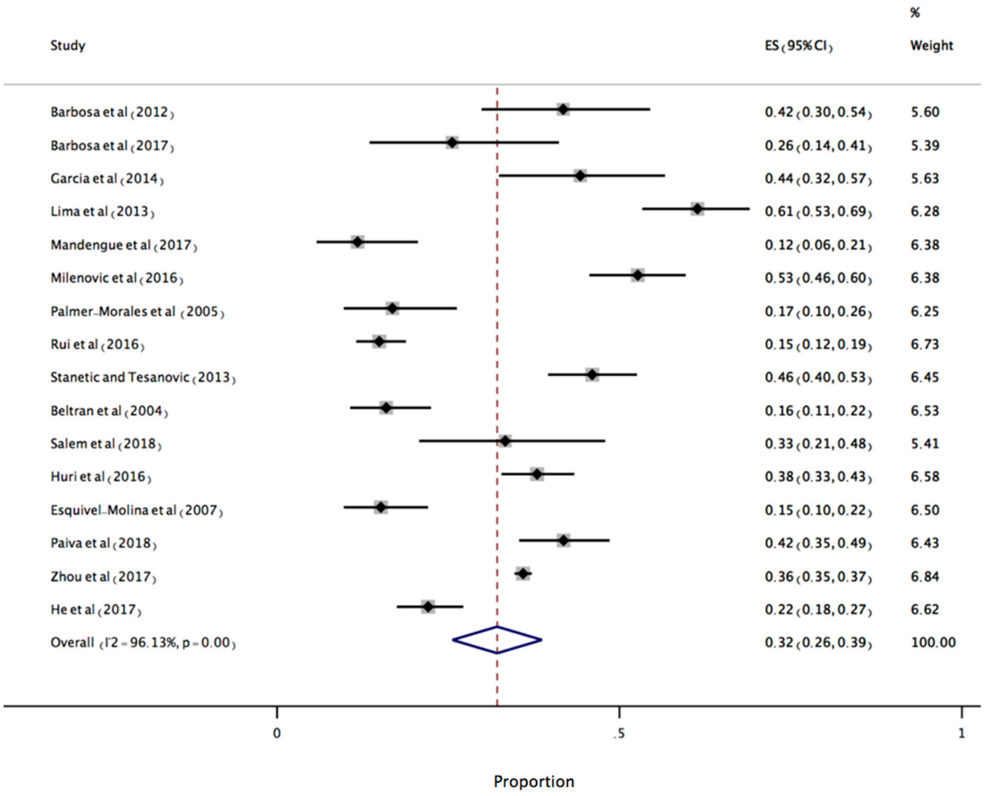

© 2020 Sabitova A et al. *JAMA Network Open.*

Sensitivity Analysis of Depersonalization Studies (Based on Results Provided as Dichotomous Data), Exclusion of Studies with Lower Quality

NB: ES=Proportion

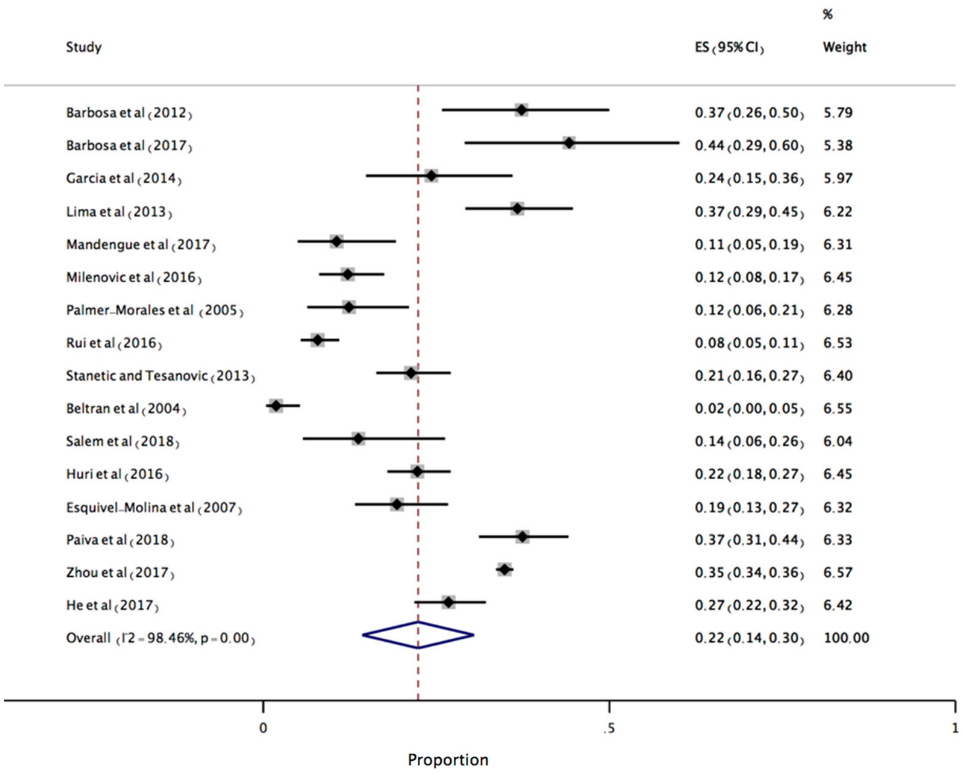

© 2020 Sabitova A et al. *JAMA Network Open.*

Sensitivity Analysis of Personal Accomplishment Studies (Based on Results Provided as Dichotomous Data), Exclusion of Studies with Lower Quality

NB: ES=Proportion

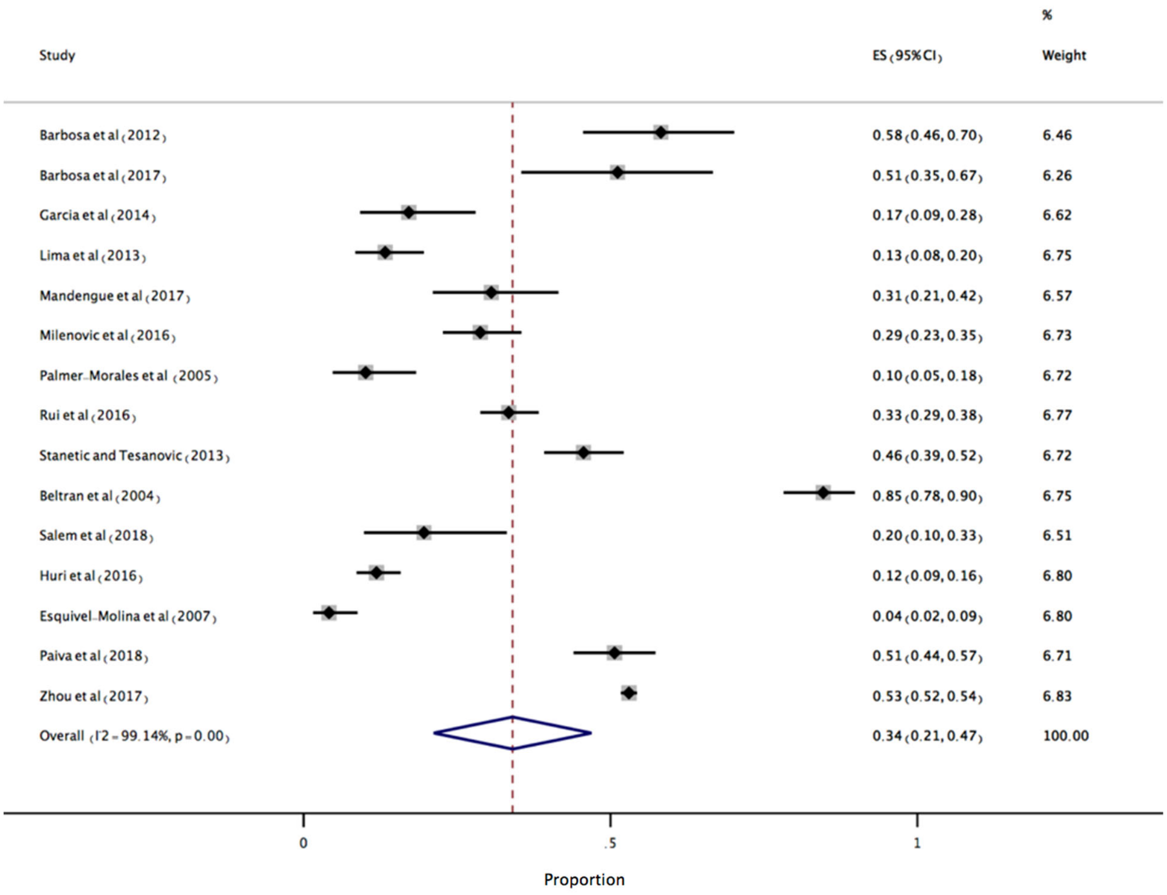

© 2020 Sabitova A et al. *JAMA Network Open.*

Sensitivity Analysis for Emotional Exhaustion Studies (Based on Results Provided as Dichotomous Data), Exclusion of Studies with Lower Quality
NB: ES=Mean score

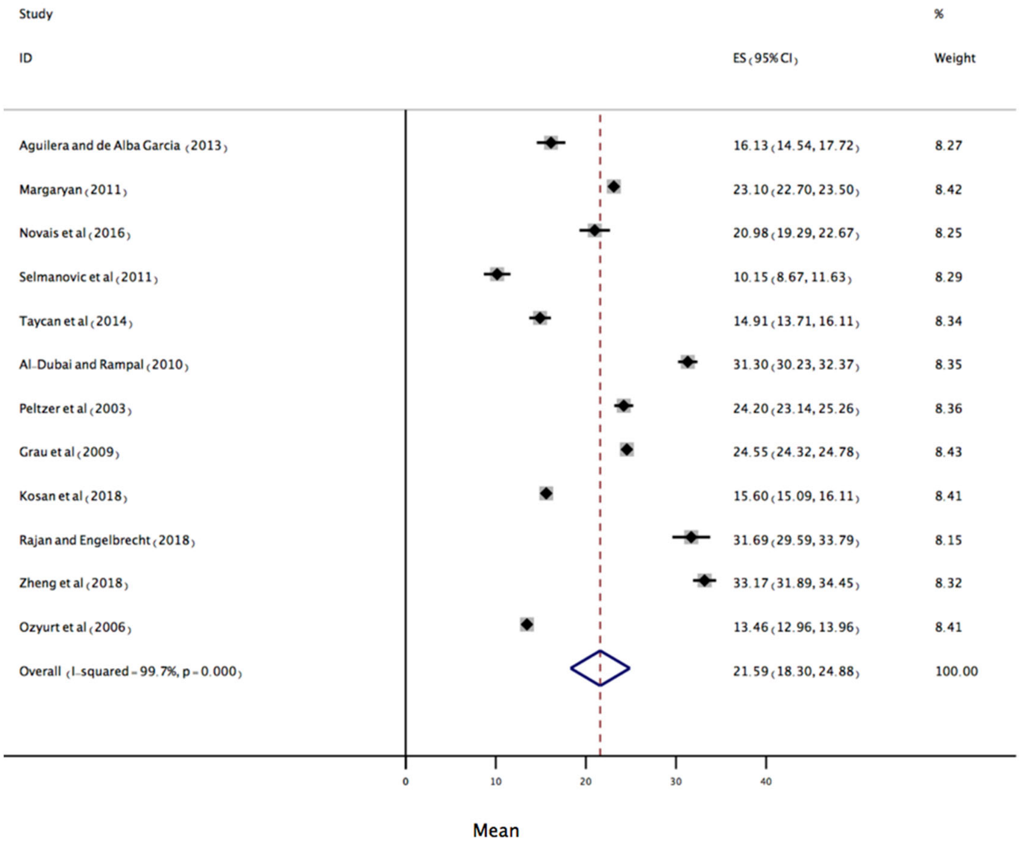

© 2020 Sabitova A et al. *JAMA Network Open.*

Sensitivity Analysis for Depersonalization Studies (Based on Results Provided as Dichotomous Data), Exclusion of Studies with Lower Quality
NB: ES=Mean score

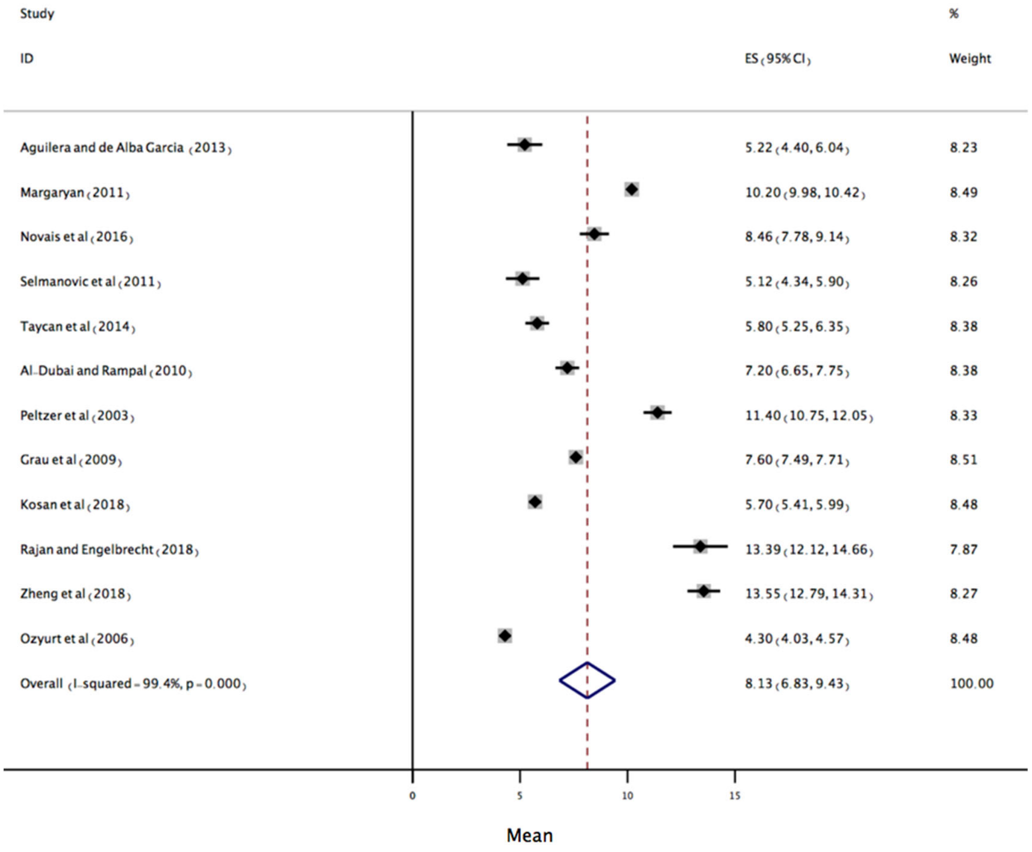

© 2020 Sabitova A et al. *JAMA Network Open.*

Sensitivity Analysis for Personal Accomplishment Studies (Based on Results Provided as Dichotomous Data), Exclusion of Studies with Lower Quality

NB: ES=Mean score

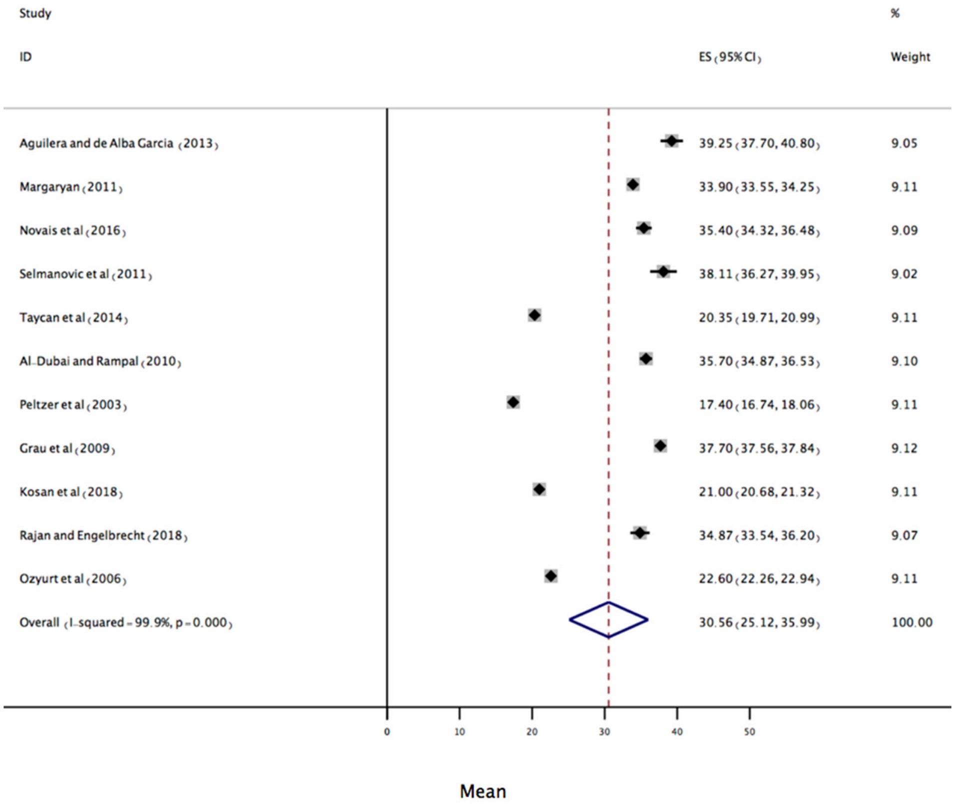

© 2020 Sabitova A et al. *JAMA Network Open.*

Sensitivity Analysis of Emotional Exhaustion Studies (Based on Results Provided as Dichotomous Data), Exclusion of Studies Where Respondents Were Not Only Qualified Physicians and/or Dentists
NB: ES=Proportion

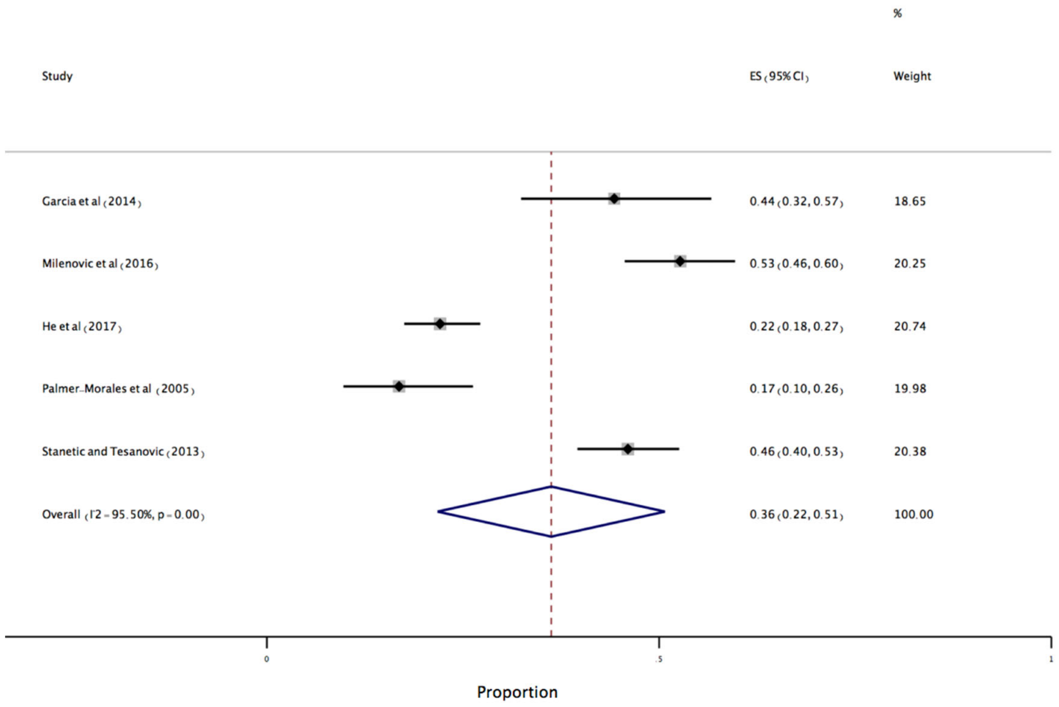

© 2020 Sabitova A et al. *JAMA Network Open.*

Sensitivity Analysis of Depersonalization Studies (Based on Results Provided as Dichotomous Data), Exclusion of Studies Where Respondents Were Not Only Qualified Physicians and/or Dentists

NB: ES=Proportion

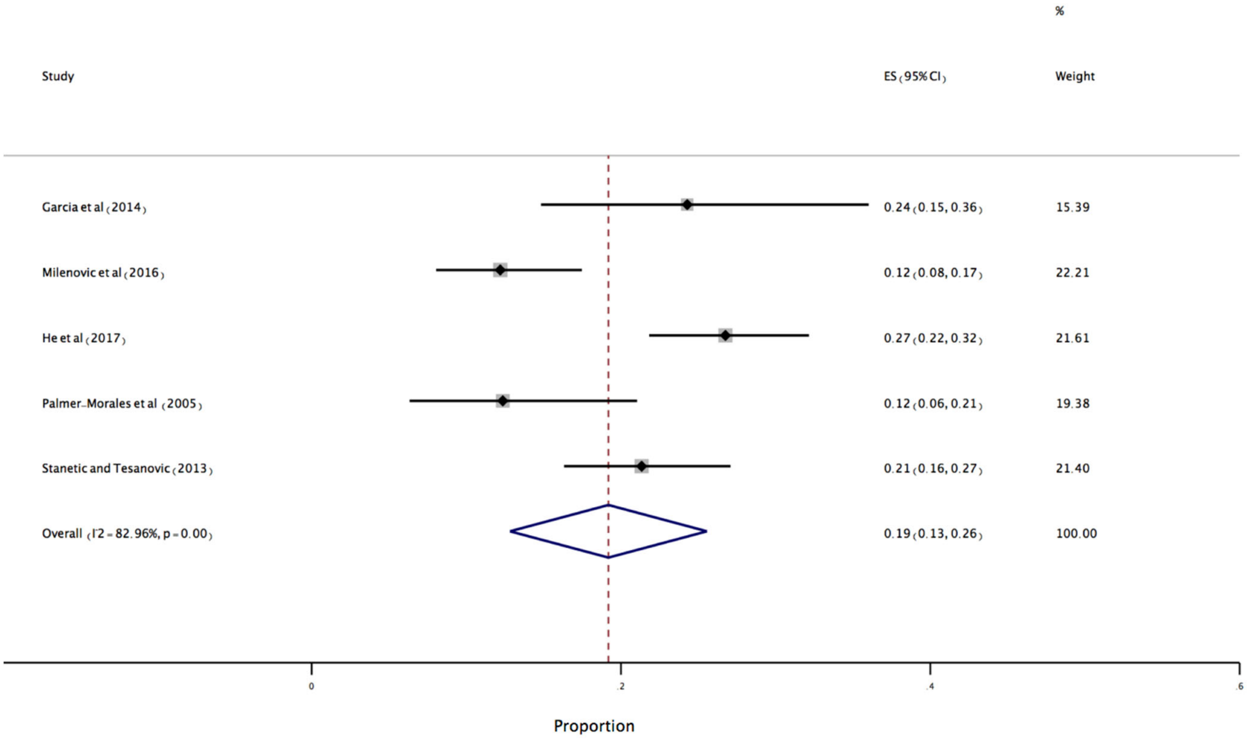

© 2020 Sabitova A et al. *JAMA Network Open.*

Sensitivity Analysis of Personal Accomplishment Studies (Based on Results Provided as Dichotomous Data), Exclusion of Studies Where Respondents Were Not Only Qualified Physicians and/or Dentists

NB: ES=Proportion

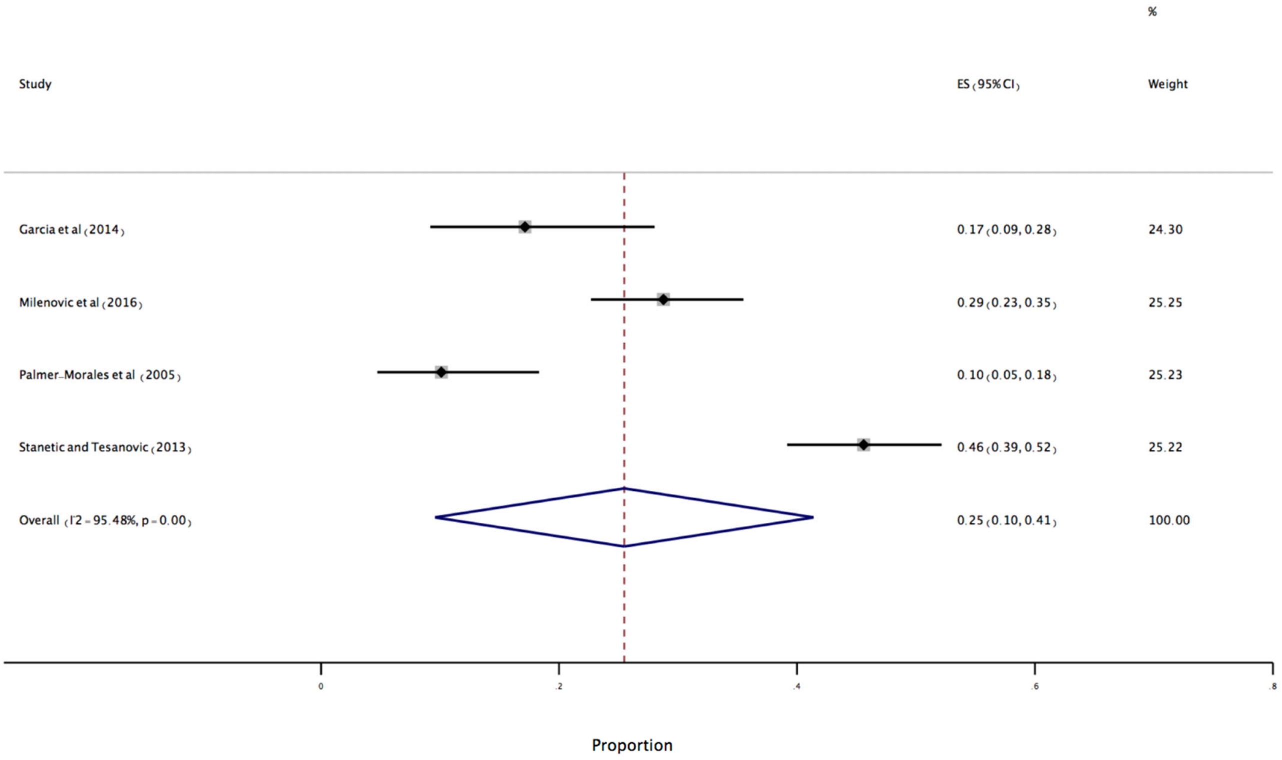

© 2020 Sabitova A et al. *JAMA Network Open.*

Sensitivity Analysis for Emotional Exhaustion Studies (Based on Results Provided as Continuous Data), Exclusion of Studies Where Respondents Were Not Only Qualified Physicians and/or Dentists

NB: ES=Mean score

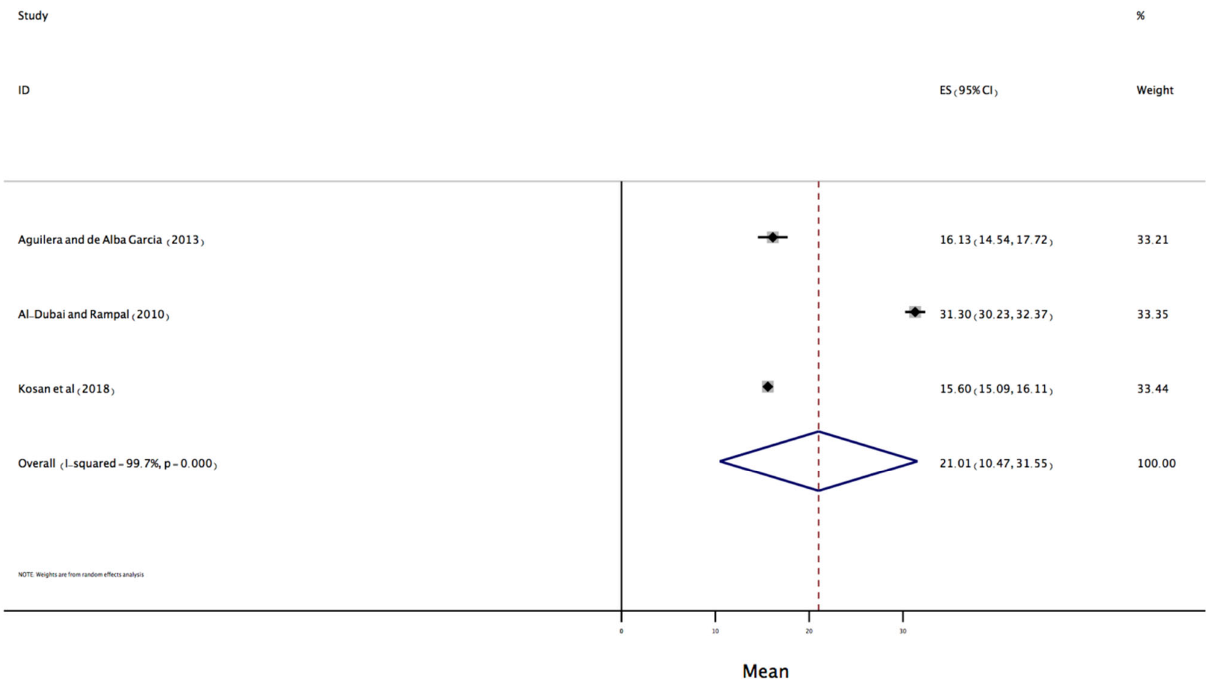

© 2020 Sabitova A et al. *JAMA Network Open.*

Sensitivity Analysis for Depersonalization Studies (Based on Results Provided as Continuous Data), Exclusion of Studies Where Respondents Were Not Only Qualified Physicians and/or Dentists

NB: ES=Mean score

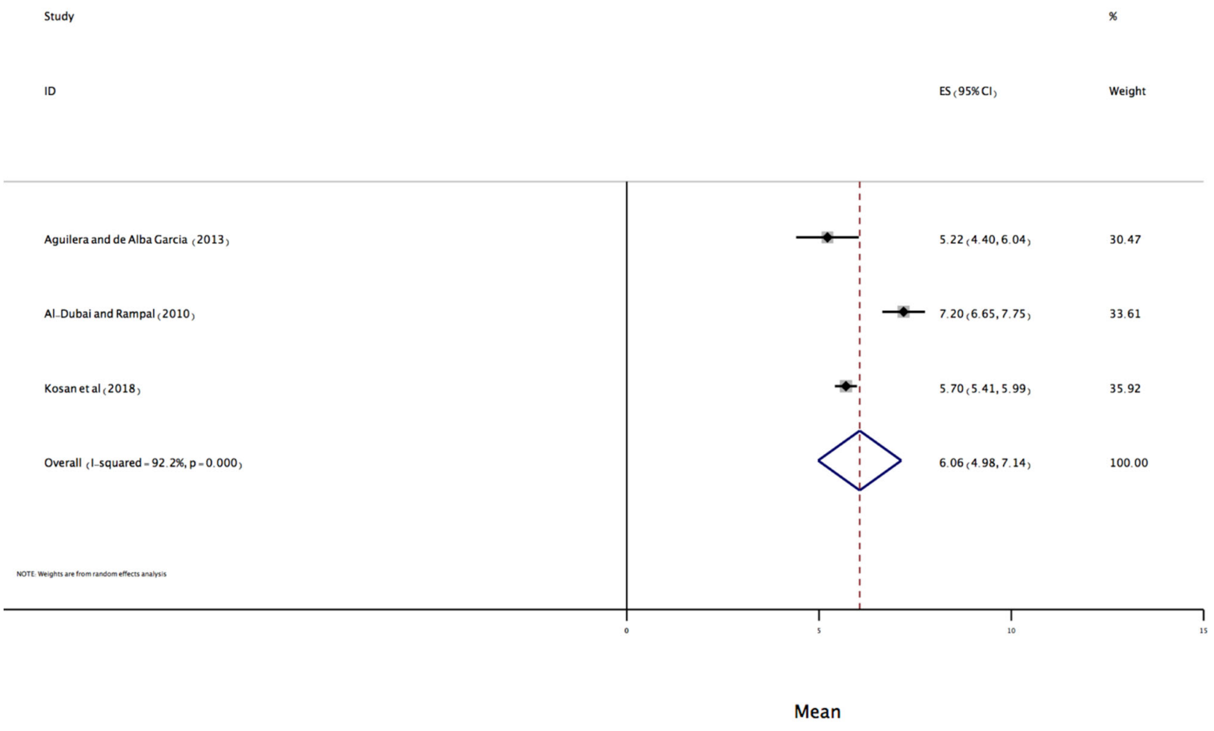

© 2020 Sabitova A et al. *JAMA Network Open.*

Sensitivity Analysis for Personal Accomplishment Studies (Based on Results Provided as Continuous Data), Exclusion of Studies Where Respondents Were Not Only Qualified Physicians and/or Dentists
NB: ES=Mean score

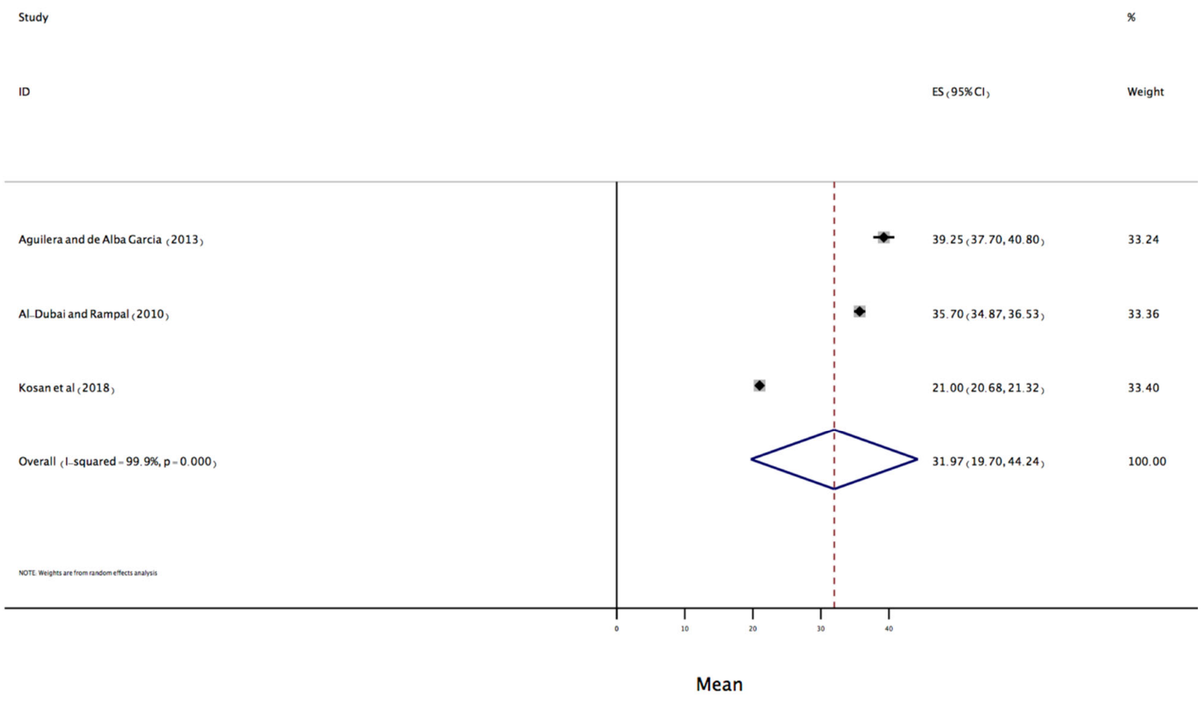

© 2020 Sabitova A et al. *JAMA Network Open.*

Sensitivity Analysis of Emotional Exhaustion Studies (Based on Results Provided as Dichotomous Data), Exclusion of Studies Where Type of Health Care Setting Was Not Reported

NB: ES=Proportion

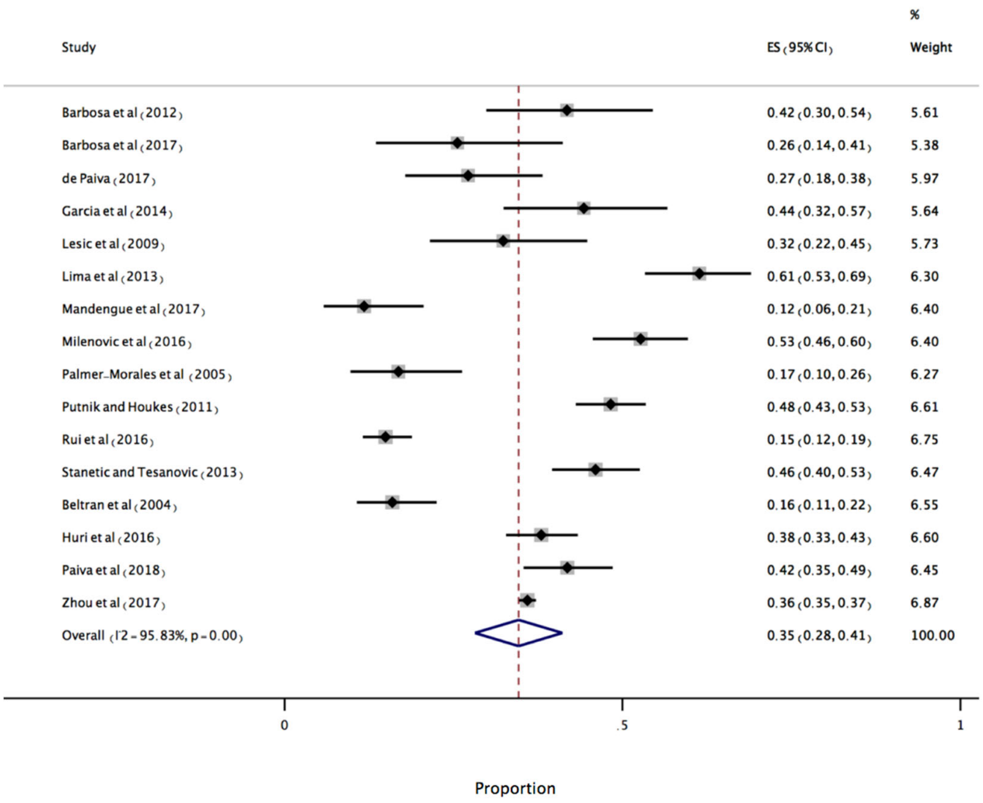

© 2020 Sabitova A et al. *JAMA Network Open.*

Sensitivity Analysis of Depersonalization Studies (Based on Results Provided as Dichotomous Data), Exclusion of Studies Where Type of Health Care Setting Was Not Reported

NB: ES=Proportion

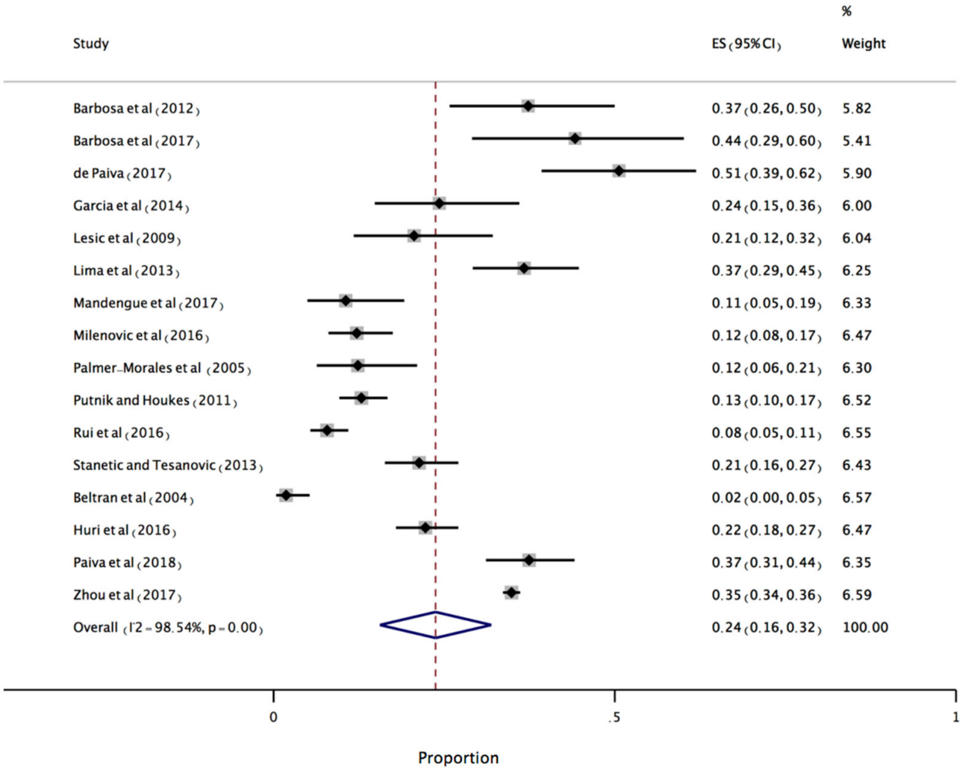

© 2020 Sabitova A et al. *JAMA Network Open.*

Sensitivity Analysis of Personal Accomplishment Studies (Based on Results Provided as Dichotomous Data), Exclusion of Studies Where Type of Health Care Setting Was Not Reported

NB: ES=Proportion

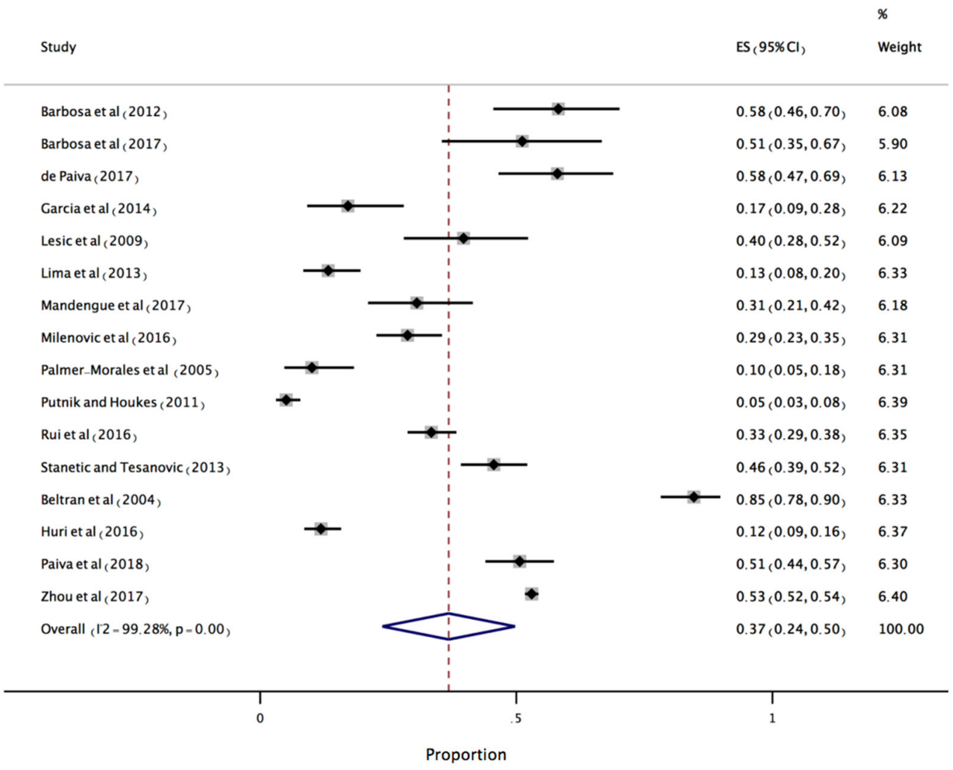

© 2020 Sabitova A et al. *JAMA Network Open.*

Sensitivity Analysis for Emotional Exhaustion Studies (Based on Results Provided as Continuous Data), Exclusion of Studies Where Type of Health Care Setting Was Not Reported
NB: ES=Mean score

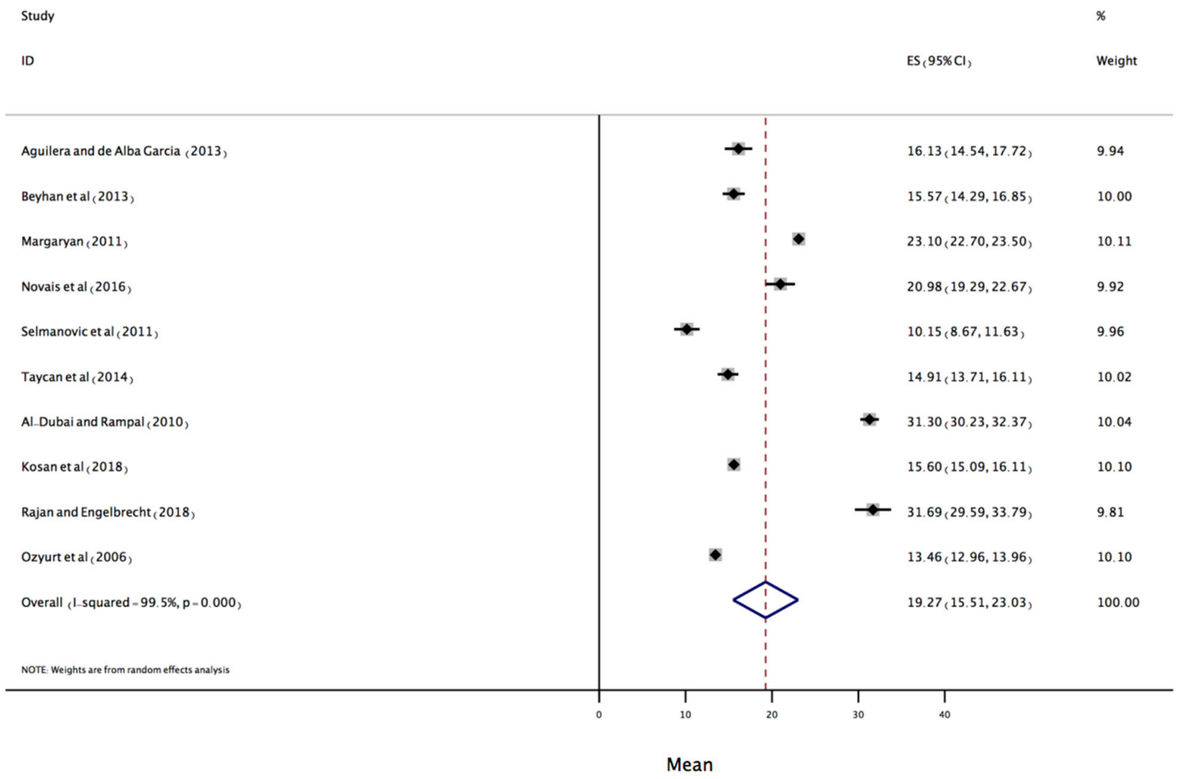

© 2020 Sabitova A et al. *JAMA Network Open.*

Sensitivity Analysis for Depersonalization Studies (Based on Results Provided as Continuous Data), Exclusion of Studies Where Type of Health Care Setting Was Not Reported

NB: ES=Mean score

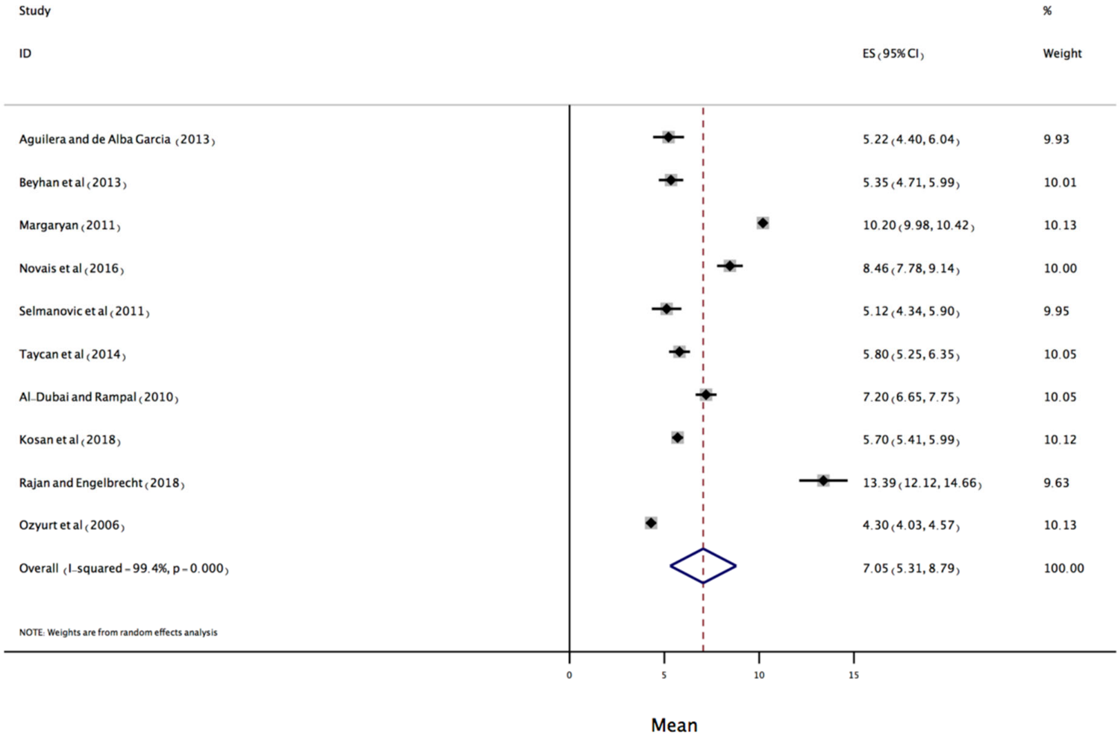

© 2020 Sabitova A et al. *JAMA Network Open.*

Sensitivity Analysis for Personal Accomplishment Studies (Based on Results Provided as Continuous Data), Exclusion of Studies Where Type of Health Care Setting Was Not Reported

NB: ES=Mean score

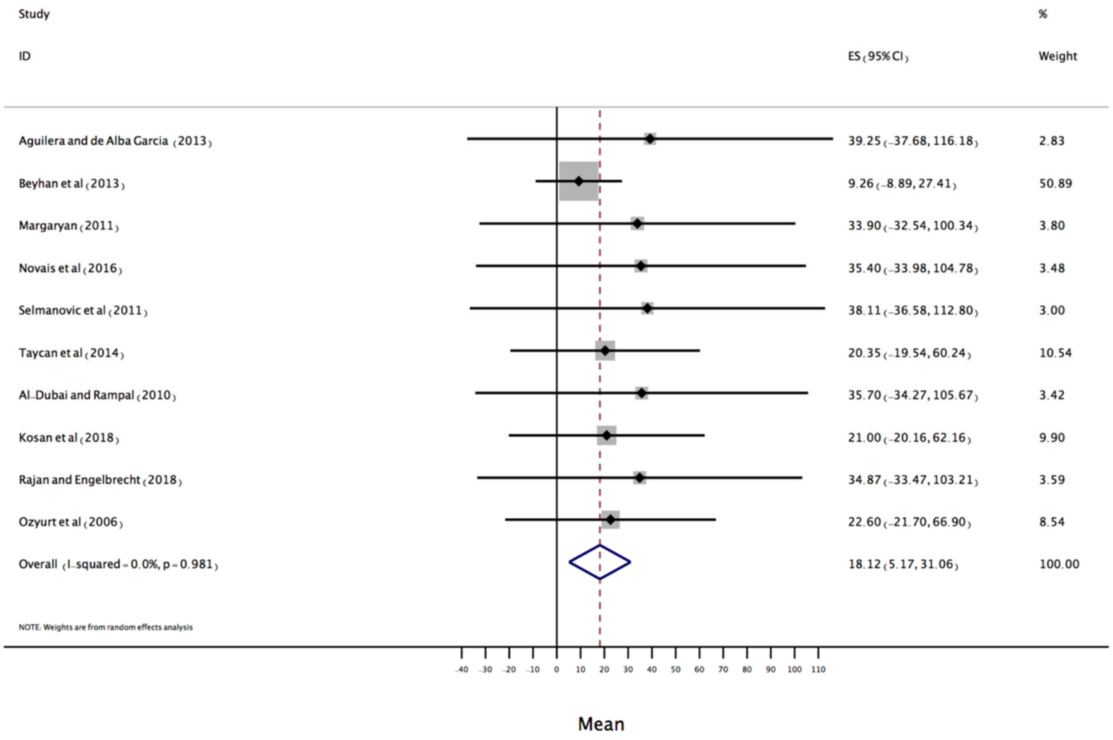

© 2020 Sabitova A et al. *JAMA Network Open.*

Funnel Plot for Emotional Exhaustion Studies (Based on Results Provided as Dichotomous Data)

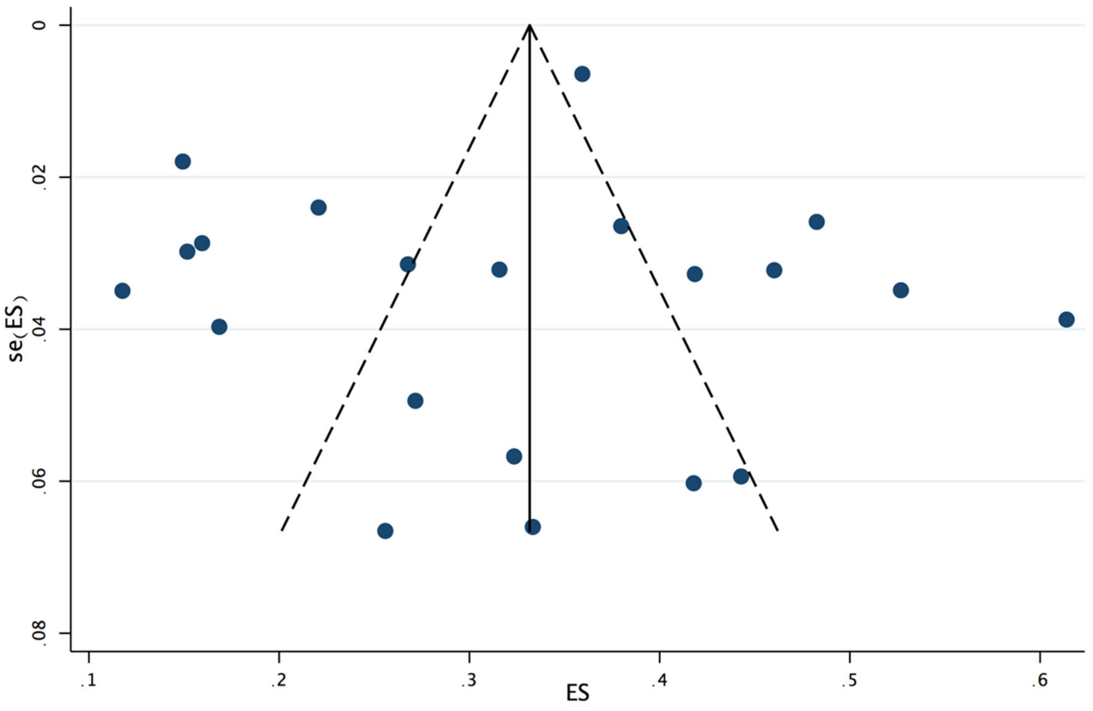

Egger's test for small-study effects:

Number of studies = 21 Root MSE = 4.71

Std_Eff	Coef.	Std. Err.	t	P>|t|	[95% Conf. Interval]	
slope	.3463349	.0357056	9.70	0.000	.2716021	.4210676
bias	-.8575549	1.614648	-0.53	0.601	-4.237052	2.521942

Test of H0: no small-study effects P = 0.601

© 2020 Sabitova A et al. *JAMA Network Open.*

Funnel Plot for Depersonalization Studies (Based on Results Provided as Dichotomous Data)

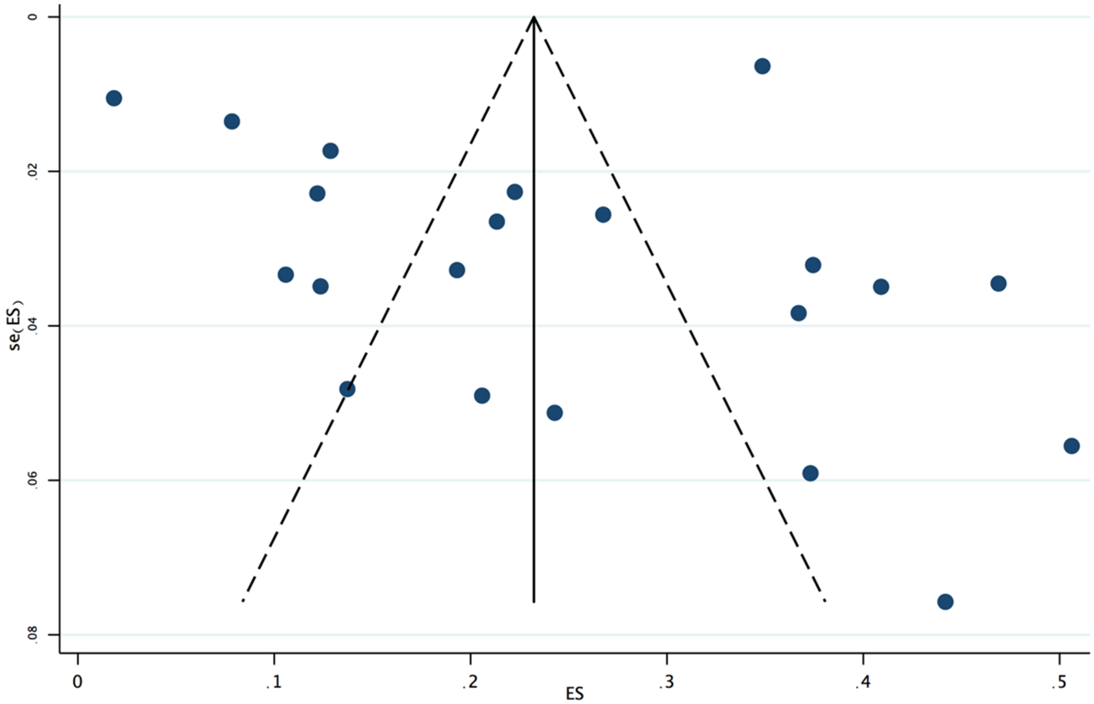

Egger's test for small-study effects:

Number of studies = 21 Root MSE = 7.631

Std_Eff	Coef.	Std. Err.	t	P>|t|	[95% Conf. Interval]	
slope	.2405411	.0515428	4.67	0.000	.1326609	.3484214
bias	-.5506936	2.690197	-0.20	0.840	-6.18134	5.079953

Test of H0: no small-study effects P = 0.840

© 2020 Sabitova A et al. *JAMA Network Open.*

Funnel Plot for Personal Accomplishment Studies (Based on Results Provided as Dichotomous Data)

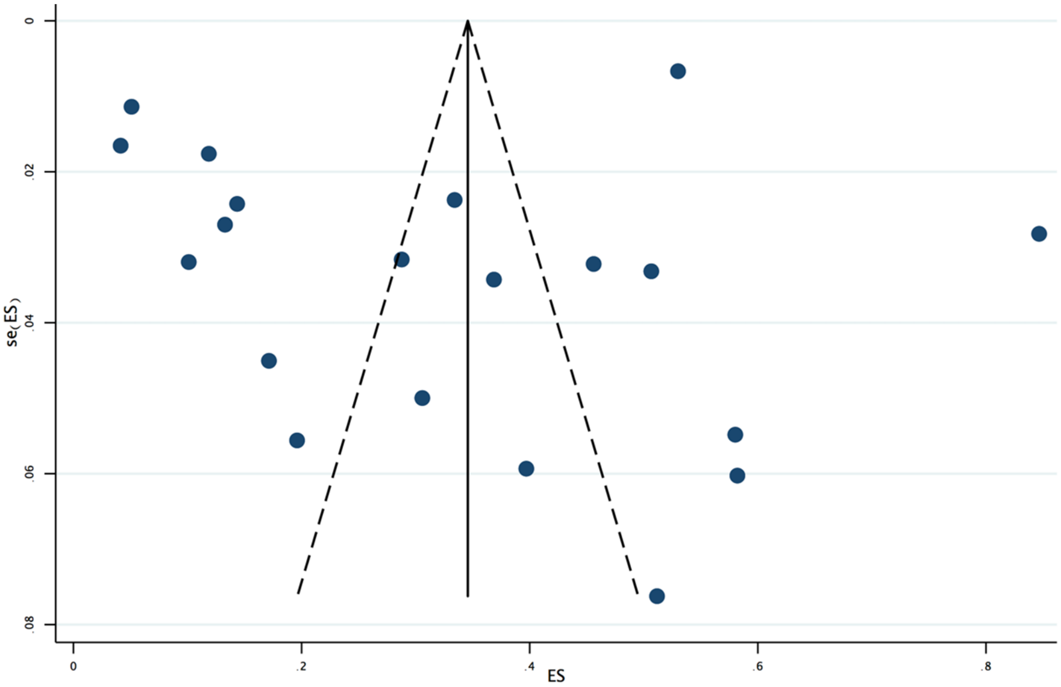

Egger's test for small-study effects:
Number of studies = 20 Root MSE = 11.69

Std_Eff	Coef.	Std. Err.	t	P>|t|	[95% Conf. Interval]	
Slope	.3980944	.0848233	4.69	0.000	.2198873	.5763015
bias	-3.324964	4.24615	-0.78	0.444	-12.24579	5.595867

Test of H0: no small-study effects P = 0.444

© 2020 Sabitova A et al. *JAMA Network Open.*

Funnel Plot for Emotional Exhaustion Studies (Based on Results Provided as Continuous Data)

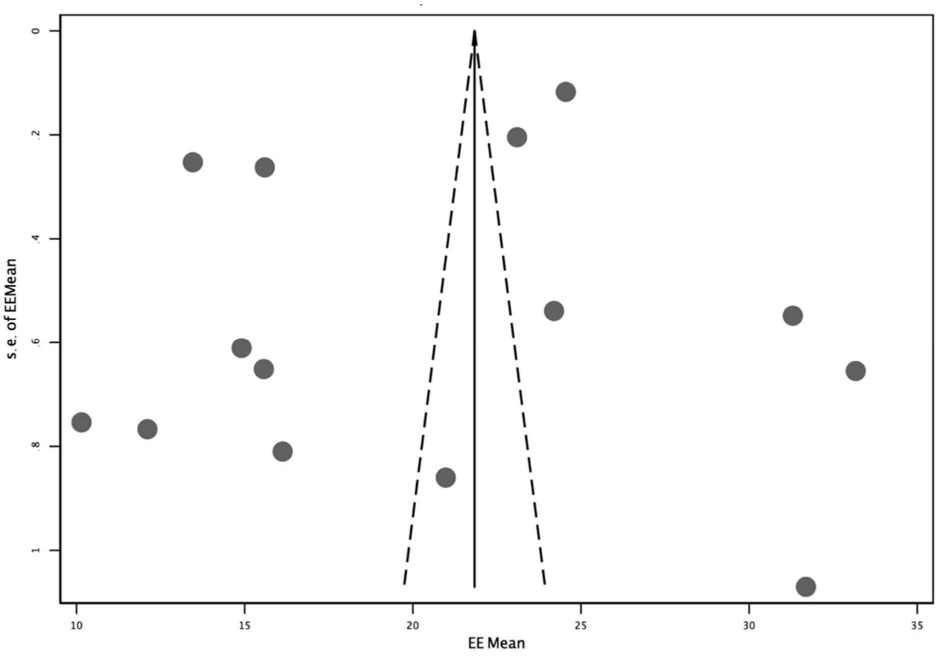

Egger's test for small-study effects:

Number of studies = 14 Root MSE = 16.75

Std_Eff	Coef.	Std. Err.	t	P>|t|	[95% Conf. Interval]	
slope.	23.44048	2.200421	10.65	0.000	18.64617	28.23478
bias	-6.685232	7.155609	-0.93	0.369	-22.27596	8.905499

Test of H0: no small-study effects P = 0.369

© 2020 Sabitova A et al. *JAMA Network Open.*

Funnel Plot for Depersonalization Studies (Based on Results Provided as Continuous Data)

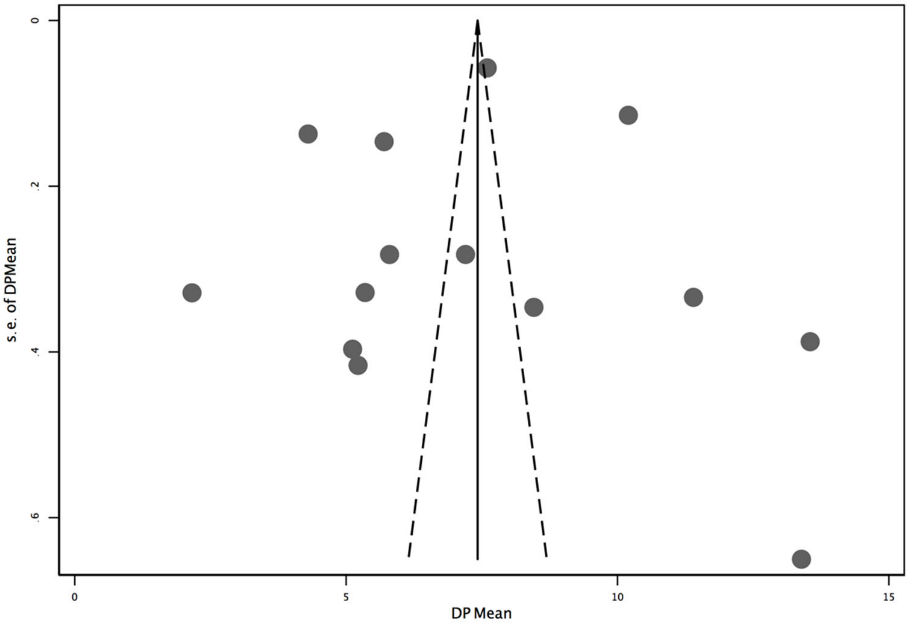

Number of studies = 14 Root MSE = 13.3

Std_Eff	Coef.	Std. Err.	t	P>|t|	[95% Conf. Interval]	
slope	7.578282	.8794551	8.62	0.000	5.662114	9.49445
bias	-1.280102	5.578686	-0.23	0.822	-13.43502	10.87481

Test of H0: no small-study effects P = 0.822

© 2020 Sabitova A et al. *JAMA Network Open.*

Funnel Plot for Personal Accomplishment Studies (Based on Results Provided as Continuous Data)

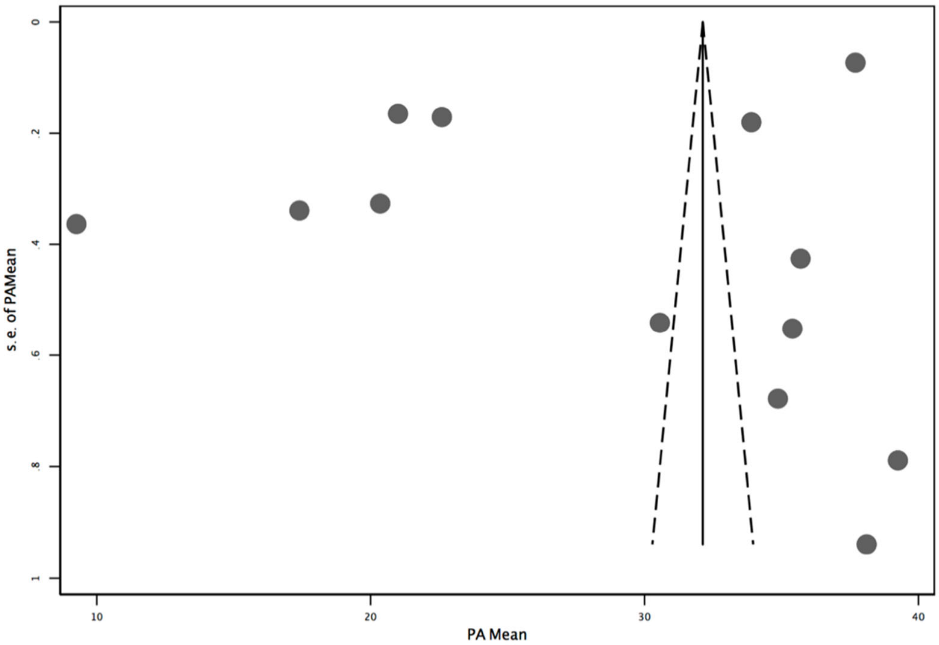

Egger's test for small-study effects:

Number of studies = 13 Root MSE = 39.28

Std_Eff	Coef.	Std. Err.	t	P>|t|	[95% Conf. Interval]	
slope	36.14644	3.302244	10.95	0.000	28.87825	43.41463
bias	-26.80311	16.67787	-1.61	0.136	-63.51085	9.904626

Test of H0: no small-study effects P = 0.136

© 2020 Sabitova A et al. *JAMA Network Open.*

eAppendix 5. Job Satisfaction

Meta-analysis of the Proportions of Physician Job Satisfaction in LMICs (Based on Results Provided as Dichotomous Data), Stratified by Country's Income Group
NB: ES=Proportion

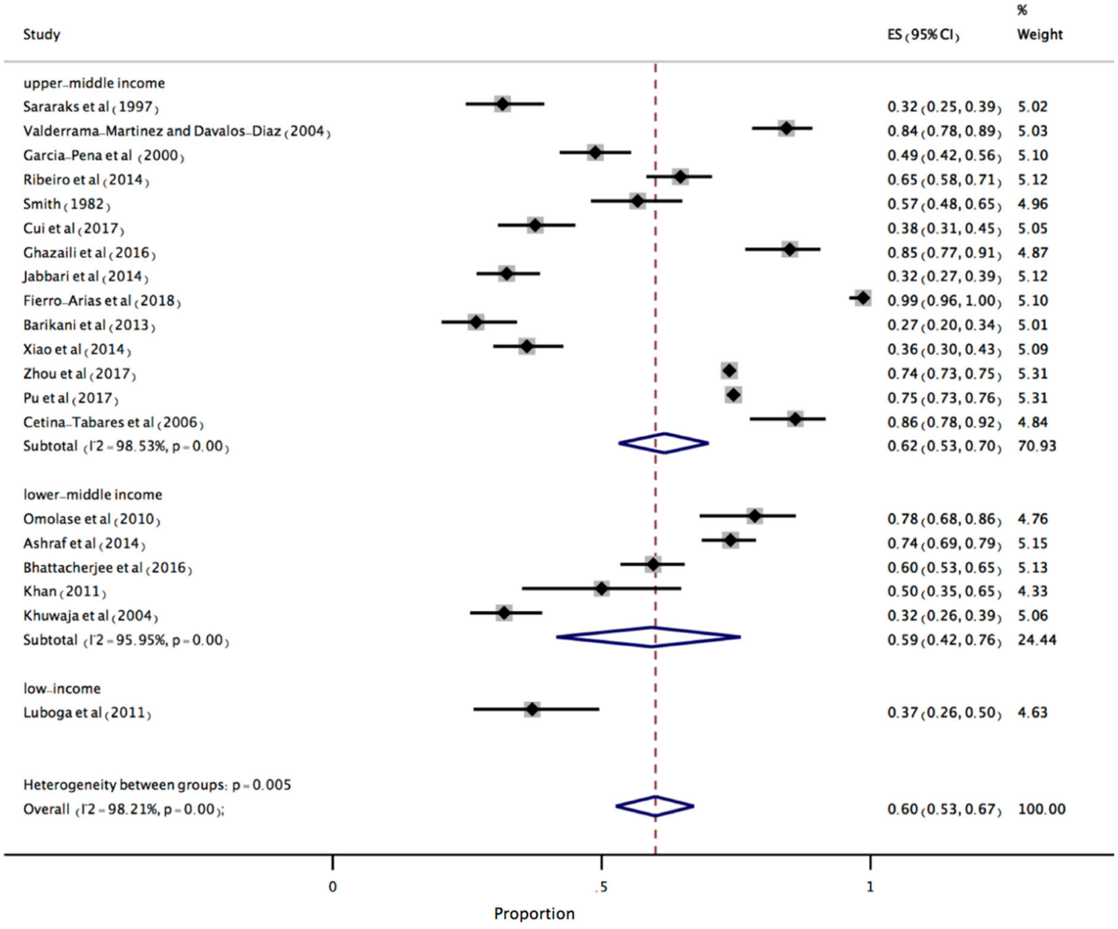

© 2020 Sabitova A et al. *JAMA Network Open.*

Metaregression of the Proportions of Physician Job Satisfaction in LMICs (Based on Results Provided as Dichotomous Data), Stratified by Country's Income Group

Meta-regression	Number of obs = 20
REML estimate of between-study variance	tau2 = 0.04764
% residual variation due to heterogeneity	I-squared_res = 91.70%
Proportion of between-study variance explained	Adj R-squared = -7.57%
Joint test for all covariates	Model F(2,17) = 0.38
With Knapp-Hartung modification	Prob > F = 0.6886

_ES	Coef.	Std. Err.	t	P>|t|	[95% Conf.	Interval]
Lower-middle income	.2182148	.2738143	0.80	0.436	-.3594829	.7959125
Upper-middle income	.2266022	.2596579	0.87	0.395	-.321228	.7744325
_cons	.3709677	.2522682	1.47	0.160	-.1612717	.9032072

© 2020 Sabitova A et al. *JAMA Network Open.*

Meta-analysis of the Proportions of Physician Job Satisfaction in LMICs (Based on Results Provided as Dichotomous Data), Stratified by Physicians' Specialities

NB: ES=Proportion

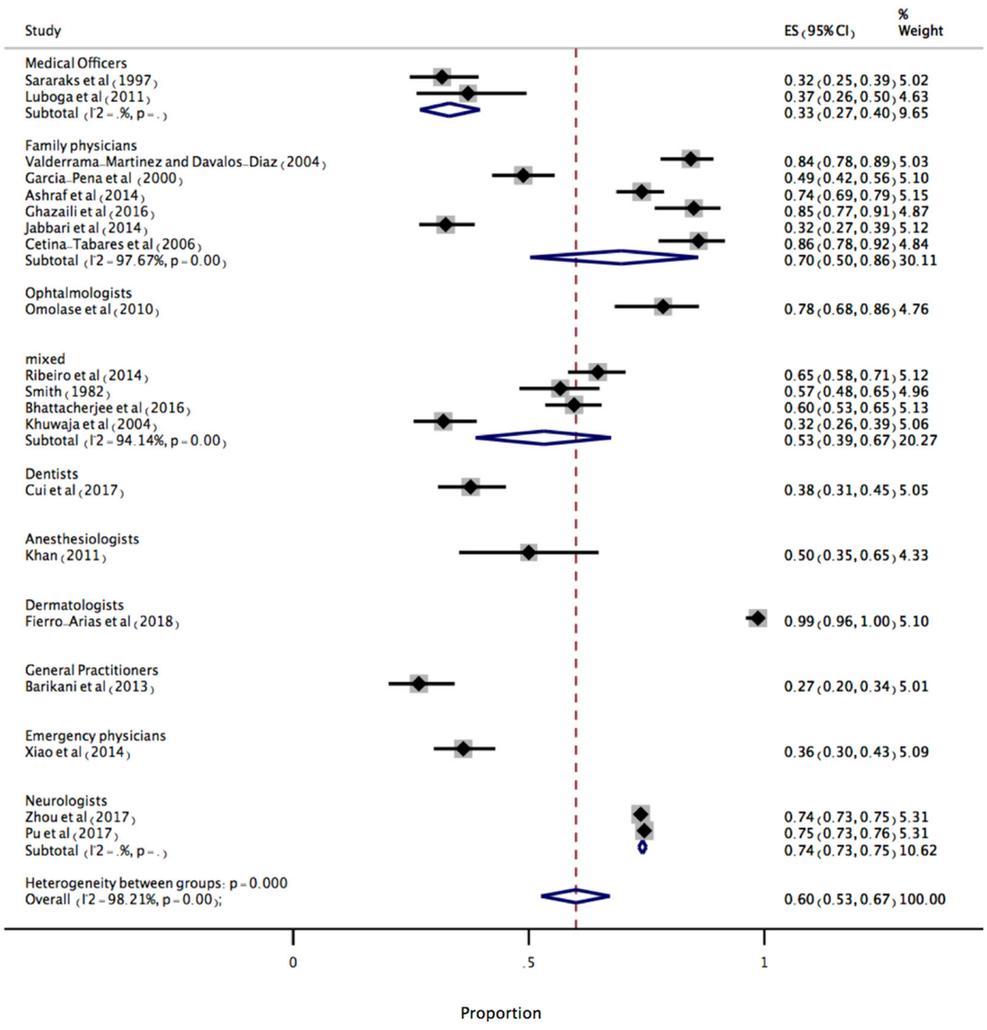

© 2020 Sabitova A et al. *JAMA Network Open.*

Metaregression of the Proportions of Physician Job Satisfaction in LMICs (Based on Results Provided as Dichotomous Data), Stratified by Physicians' Specialities

Meta-regression Number of obs = 20
REML estimate of between-study variance tau2 = .02653
% residual variation due to heterogeneity I-squared_res = 83.34%
Proportion of between-study variance explained Adj R-squared = 40.09%
Joint test for all covariates Model F(9,10) = 2.13
With Knapp-Hartung modification Prob > F = 0.1276

_ES	Coef.	Std. Err.	t	P>|t|	[95% Conf. Interval]	
anaesthesiologists	-.0333708	.2432797	-0.14	0.894	-.5754318	.5086902
dentists	-.1569002	.2008949	-0.78	0.453	-.604522	.2907216
dermatologists	.4529306	.1976094	2.29	0.045	.0126294	.8932318
emergency physicians	-.1723952	.1983932	-0.87	0.405	-.6144427	.2696523
family physicians	.1397373	.1159073	1.21	0.256	-.1185202	.3979949
general practitioners	-.2667041	.2028255	-1.31	0.218	-.7186275	.1852193
medical officers	-.1934371	.1630095	-1.19	0.263	-.556645	.1697707
neurologists	.2080287	.1460042	1.42	0.185	-.1172888	.5333463
ophthalmologists	.2514393	.2169615	1.16	0.273	-.231981	.7348596
_cons	.5333708	.0892266	5.98	0.000	.3345616	.73218

© 2020 Sabitova A et al. *JAMA Network Open.*

Meta-analysis of the Proportions of Physician Job Satisfaction in LMICs (Based on Results Provided as Dichotomous Data), Stratified by Geographical Regions
NB: ES=Proportion

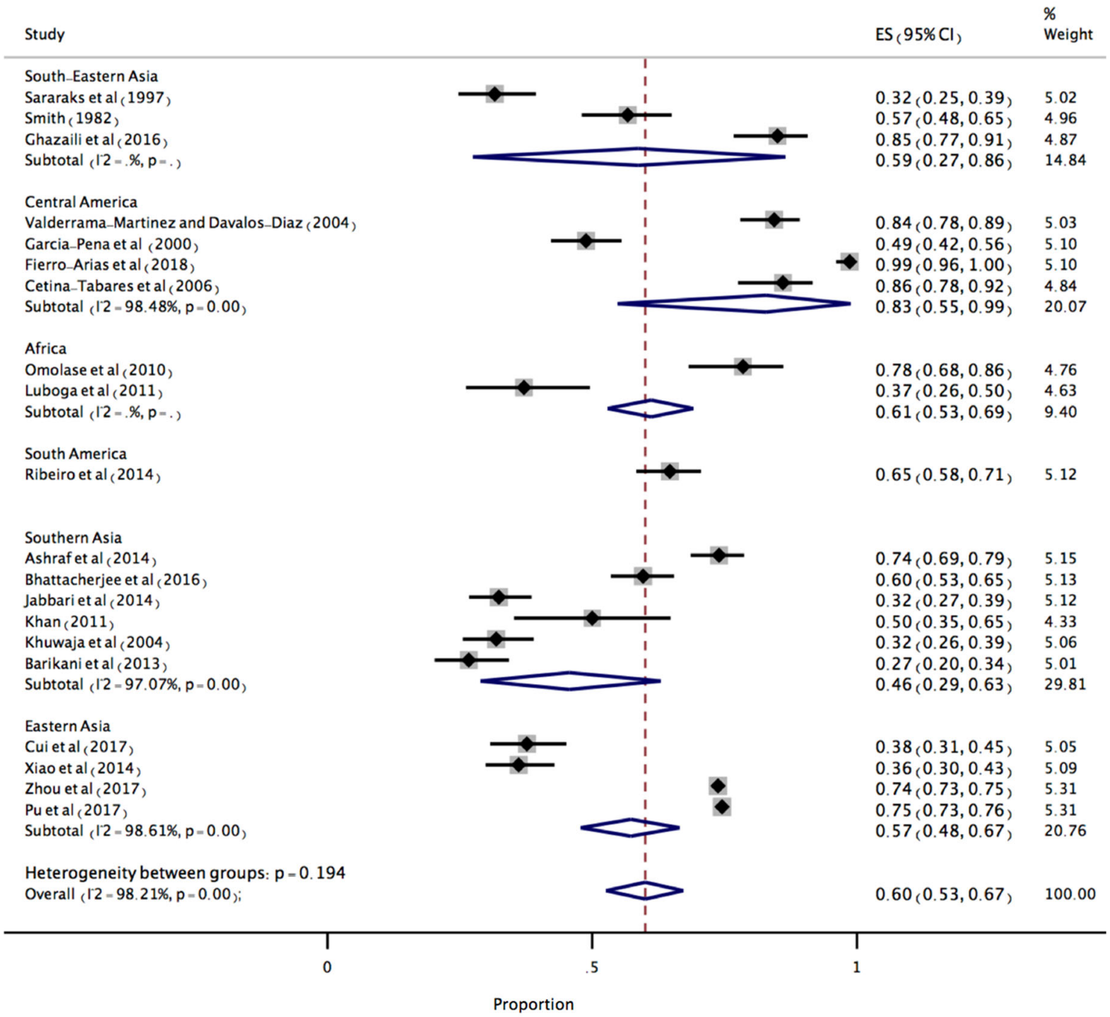

© 2020 Sabitova A et al. *JAMA Network Open.*

Sensitivity Analysis of Job Satisfaction Studies (Based on Results Provided as Dichotomous Data), Exclusion of Studies With Lower Quality
NB: ES=Proportion

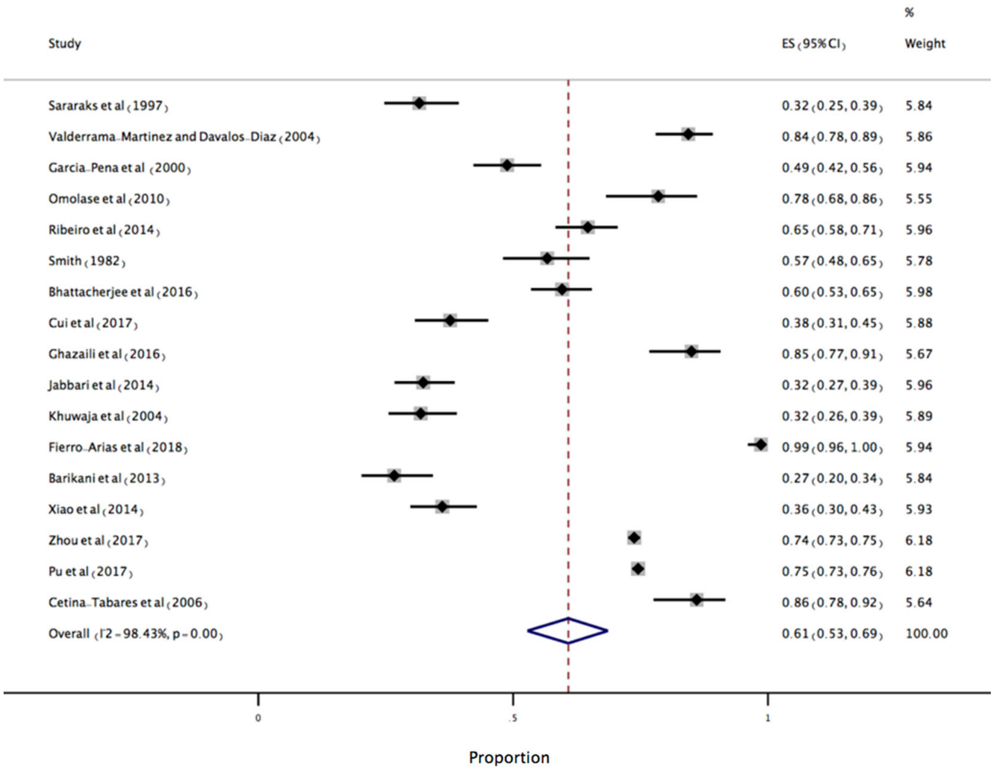

© 2020 Sabitova A et al. *JAMA Network Open.*

Sensitivity Analysis of Job Satisfaction Studies (Based on Results Provided as Dichotomous Data), Exclusion of Studies Where Type of Health Care Setting Was Not Reported

NB: ES=Proportion

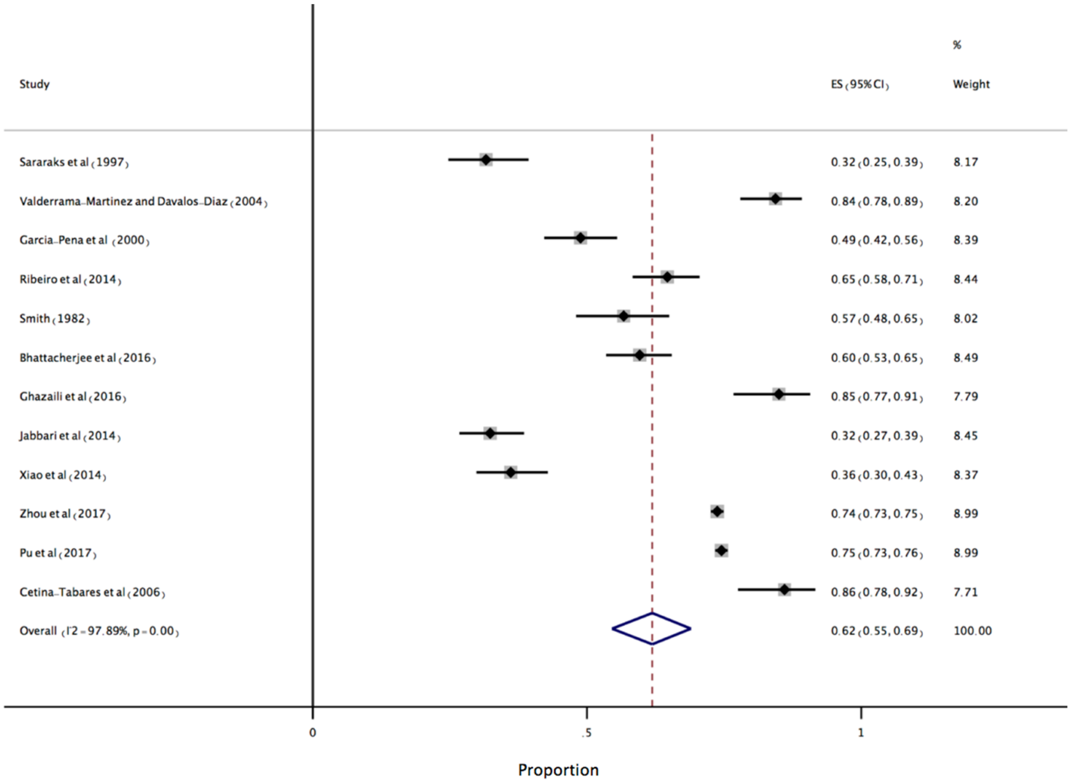

© 2020 Sabitova A et al. *JAMA Network Open.*

Sensitivity Analysis of Job Satisfaction Studies (Based on Results Provided as Dichotomous Data), Exclusion of Studies Where Respondents Were Not Only Wualified Physicians and/or Dentists

NB: ES=Proportion

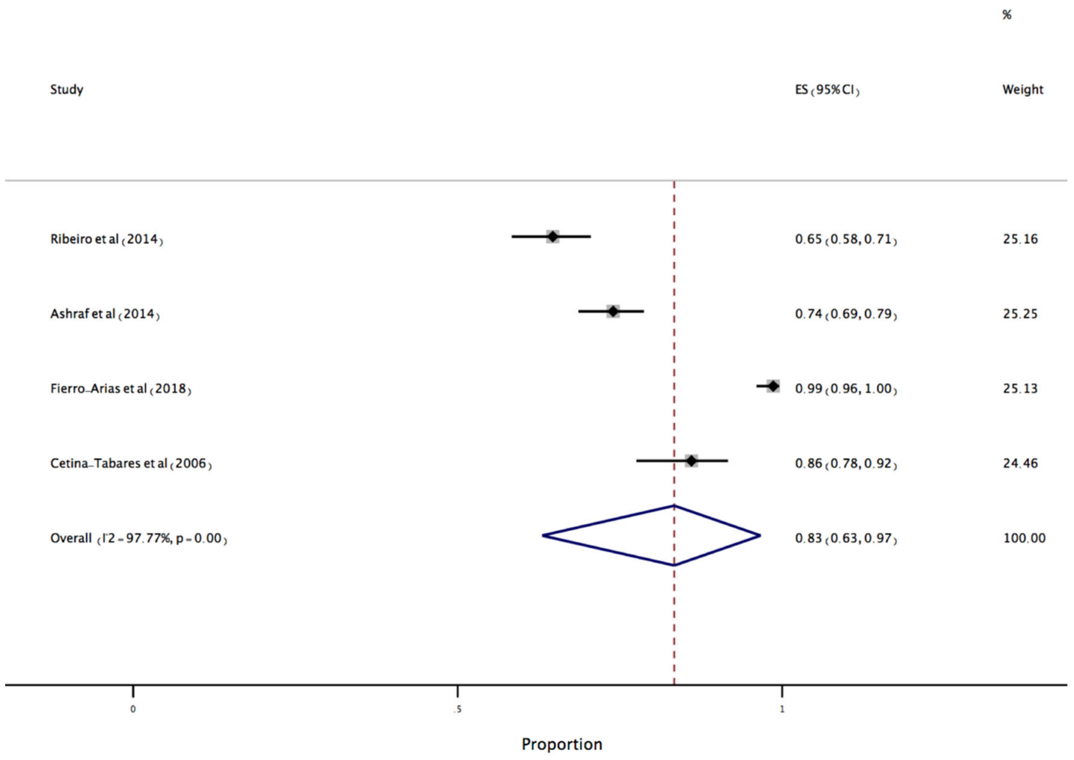

© 2020 Sabitova A et al. *JAMA Network Open.*

Funnel Plot for Job Satisfaction Studies (Based on Results Provided as Dichotomous Data)

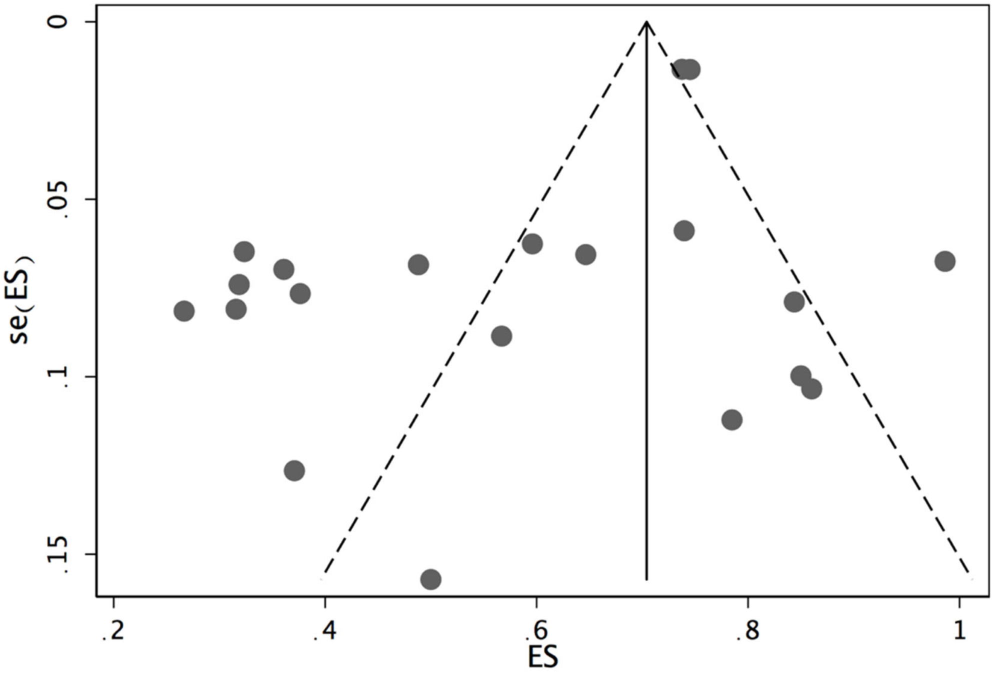

Number of studies = 20 Root MSE = 2.941

Std_Eff	Coef.	Std. Err.	t	P>|t|	[95% Conf. Interval]	
Slope	.7723467	.0349046	22.13	0.000	.6990149	.8456785
Bias	-2.57226	.9275365	-2.77	0.013	-4.520942	-.6235781

Test of H0: no small-study effects P = 0.013

© 2020 Sabitova A et al. *JAMA Network Open.*

Funnel Plot for Job Satisfaction Studies (Based on Results Provided as Continuous Data on a Scale Ranging From 1 to 5)

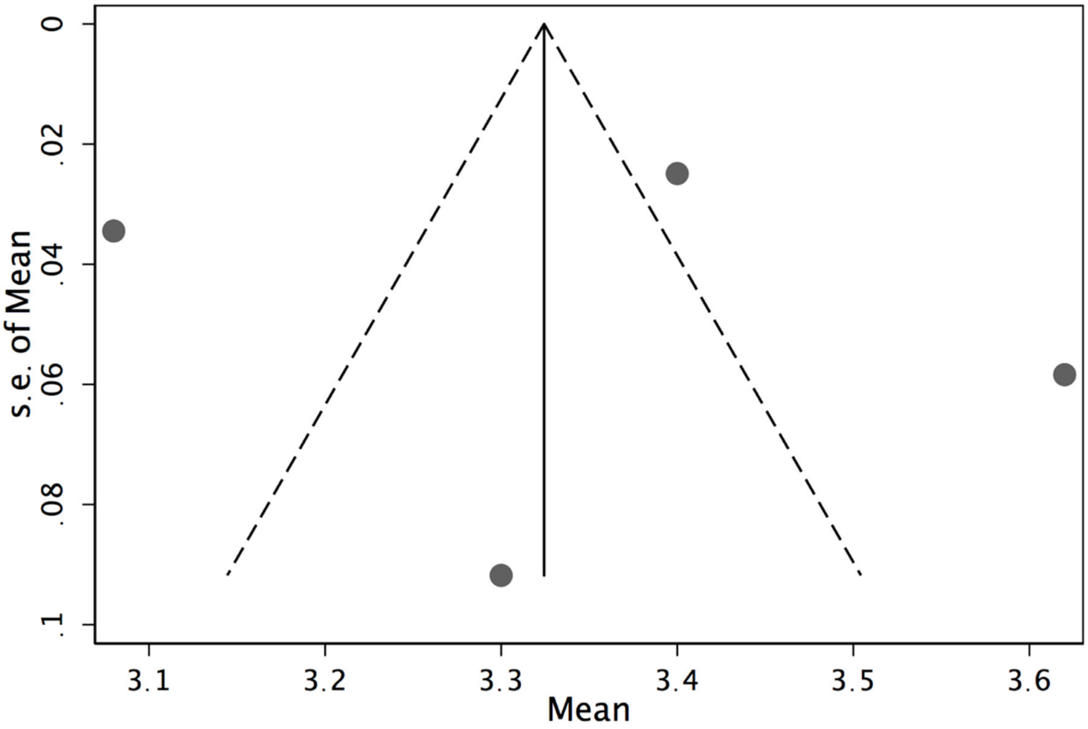

Number of studies = 4 Root MSE = 6.497

Std_Eff	Coef.	Std. Err.	t	P>|t|	[95% Conf. Interval]	
slope	3.288658	.2891712	11.37	0.008	2.044455	4.532861
bias	1.053931	7.74091	0.14	0.904	-32.25252	34.36038

Test of H0: no small-study effects P = 0.904

© 2020 Sabitova A et al. *JAMA Network Open.*

eAppendix 6. Risk of Bias of Included Studies

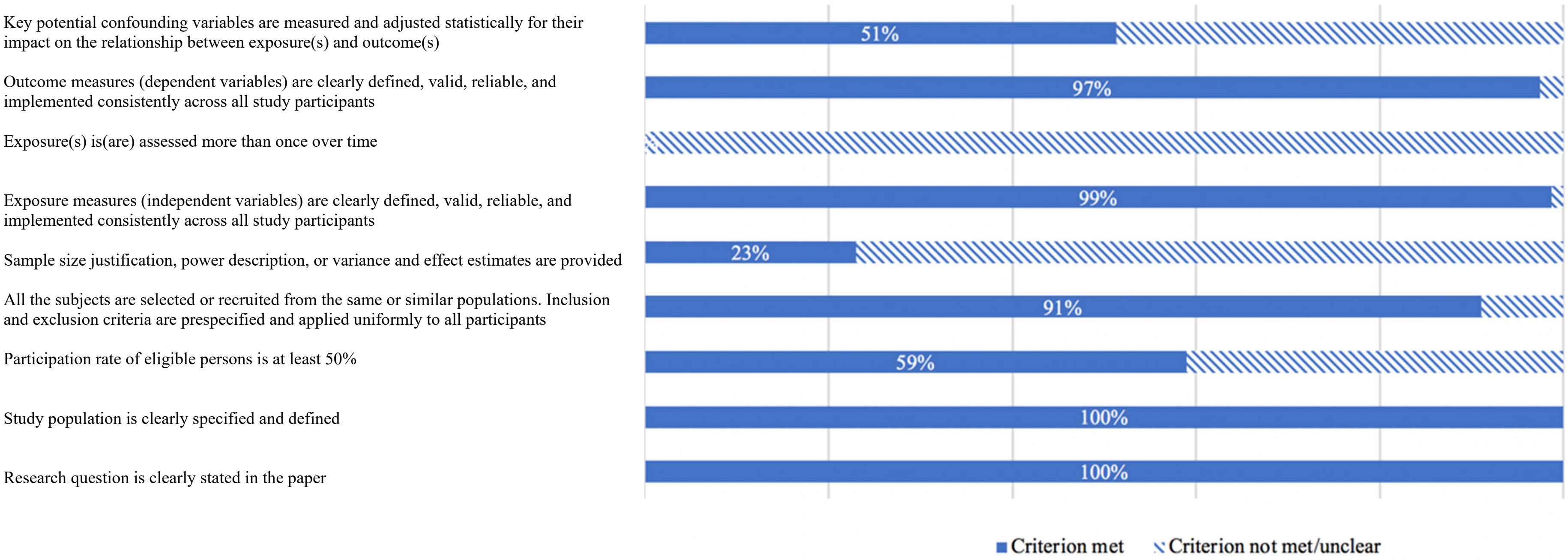

© 2020 Sabitova A et al. *JAMA Network Open.*

eReferences

1. Aguilera EC, De Alba García JEG. Analysis of Possible Sociodemographic and Occupational Risk Factors and the Prevalence of Professional Exhaustion Syndrome (Burnout) in Mexican Dentists. *Revista Colombiana de Psiquiatria.* 2013;42(2):182-190.
2. Barbosa FT, Leao BA, Tavares GMS, dos Santos J. Burnout syndrome and weekly workload of on-call physicians: cross-sectional study. *Sao Paulo Med J.* 2012;130(5):282-288.
3. Barbosa FT, Eloi RJ, dos Santos LM, Leão BA, Lima FJCD, de Sousa-Rodrigues CF. Correlation between weekly working time and burnout syndrome among anesthesiologists of Maceió-AL. *Brazilian Journal of Anesthesiology.* 2017;67(2):115-121.
4. Beyhan S, Güneş Y, Türktan M, Özcengiz D. Investigation of the burnout syndrome among the Eastern Mediterranean Region anaesthesiologists. *Turk Anesteziyoloji ve Reanimasyon Dernegi Dergisi.* 2013;41(1):7-13.
5. de Paiva LC, Canario ACG, de Paiva China ELC, Goncalves AK. Burnout syndrome in health-care professionals in a university hospital. *Clinics (Sao Paulo, Brazil).* 2017;72(5):305-309.
6. Erdur B, Ergin A, Yuksel A, Turkcuer I, Ayrik C, Boz B. Assessment of the relation of violence and burnout among physicians working in the emergency departments in Turkey. *Ulus Travma Acil Cerrahi Derg.* 2015;21(3):175-181.
7. Garcia TT, Garcia PC, Molon ME, et al. Prevalence of burnout in pediatric intensivists: an observational comparison with general pediatricians. *Pediatric critical care medicine : a journal of the Society of Critical Care Medicine and the World Federation of Pediatric Intensive and Critical Care Societies.* 2014;15(8):e347-353.
8. Jugale PV, Mallaiah P, Krishnamurthy A, Sangha R. Burnout and Work Engagement Among Dental Practitioners in Bangalore City: A Cross-Sectional Study. *Journal of clinical and diagnostic research : JCDR.* 2016;10(2):Zc63-67.
9. Lesic AR, Stefanovic NP, Perunicic I, Milenkovic P, Tosevski DL, Bumbasirevic MZ. Burnout in Belgrade orthopaedic surgeons and general practitioners, a preliminary report. *Acta chirurgica Iugoslavica.* 2009;56(2):53-59.
10. Liadova AV, Korkiya ED, Mamedov AK, Panich NA. The burnout among emergency physicians: Evidence from Russia (sociological study). *Man in India.* 2017;97(15):495-507.
11. Lima RAD, de Souza AI, Galindo RH, Feliciano KVD. Vulnerability to burnout among physicians at a public hospital in Recife. *Cienc Saude Coletiva.* 2013;18(4):1051-1058.
12. Mandengue SH, Owona Manga LJ, Lobe-Tanga MY, et al. [Burnout syndrome among generalist medical doctors of Douala region (Cameroon): Can physical activities be a protective factor?]. *Revue medicale de Bruxelles.* 2017;38(1):10-15.
13. Margaryan AG. Health and well-being of Armenian physicians. *New Armenian Medical Journal.* 2011;5(3):37-40.
14. Milenovic M, Matejic B, Vasic V, Frost E, Petrovic N, Simic D. High rate of burnout among anaesthesiologists in Belgrade teaching hospitals: Results of a cross-sectional survey. *European journal of anaesthesiology.* 2016;33(3):187-194.
15. Novais RN, Rocha LM, Eloi RJ, et al. Burnout Syndrome prevalence of on-call surgeons in a trauma reference hospital and its correlation with weekly workload: cross-sectional study. *Revista do Colegio Brasileiro de Cirurgioes.* 2016;43(5):314-319.
16. Hamama L, Tartakovsky E, Patrakov E. Attidudes and burnout among heath care professionals working with HIV/AIDS *Vopr Psikhologii.* 2016(3):79-+.
17. He Y, Pang Y, Zhang Y, Fielding R, Tang L. Dual role as a protective factor for burnout-related depersonalization in oncologists. *Psycho-oncology.* 2017;26(8):1080-1086.
18. Palmer Y, Gómez-Vera A, Cabrera-Pivaral C, Prince-Vélez R, Searcy R. Organizational factors associated to Burnout Syndrome among anesthesiologists. *Salud Mental.* 2005;28(1):82-91.
19. Palmer-Morales LY, Gómez-Vera A, Cabrera-Pivaral C, Prince-Velez R, Searcy-Bernal R. Prevalence of Burnout syndrome among anesthesiologists in Mexicali. *Gaceta Medica de Mexico.* 2005;141(3):181-183.

© 2020 Sabitova A et al. *JAMA Network Open.*

20. Parfenov IA. [Psychophysiological characteristics of professional burnout syndrome in doctors of various specialties and different age groups]. *Advances in gerontology = Uspekhi gerontologii / Rossiĭskai{combining double inverted breve}a akademii{combining double inverted breve}a nauk, Gerontologicheskoe obshchestvo.* 2012;25(4):736-744.
21. Popa F, Arafat R, Purcarea VL, Lala A, Popa-Velea O, Bobirnac G. Occupational burnout levels in emergency medicine--a stage 2 nationwide study and analysis. *Journal of medicine and life.* 2010;3(4):449-453.
22. Porto GG, Carneiro SC, Vasconcelos BC, Nascimento MM, Leal JL. Burnout syndrome in oral and maxillofacial surgeons: a critical analysis. *International journal of oral and maxillofacial surgery.* 2014;43(7):894-899.
23. Putnik K, Houkes I. Work related characteristics, work-home and home-work interference and burnout among primary healthcare physicians: a gender perspective in a Serbian context. *BMC public health.* 2011;11:716.
24. Rui M, Ting C, Fang PQ, Fu XQ. Burnout among anaesthetists in Chinese hospitals: a multicentre, cross-sectional survey in 6 provinces. *J Eval Clin Pract.* 2016;22(3):387-394.
25. Selmanovic S, Ramic E, Pranjic N, Brekalo-Lazarevic S, Pasic Z, Alic A. Stress at work and burnout syndrome in hospital doctors. *Medicinski arhiv.* 2011;65(4):221-224.
26. Stanetic K, Tesanovic G. Influence of age and length of service on the level of stress and burnout syndrome. *Medicinski pregled.* 2013;66(3-4):153-162.
27. Tejada P, Gomez V. Prevalence and both Demographic and Work Factors Associated with Burnout of Psychiatrists in Colombia. *Univ Psychol.* 2012;11(3):863-873.
28. Zhou J, Yang Y, Qiu X, et al. Relationship between Anxiety and Burnout among Chinese Physicians: A Moderated Mediation Model. *PloS one.* 2016;11(8):e0157013.
29. Freire PL, Trentin JP, Quevedo LD. Trends in burnout syndrome and emotional factors: an assessment of anesthesiologists in Southern Brazil, 2012. *Psychology Health & Medicine.* 2016;21(4):413-423.
30. Montenegro S, Elena GA, Tarrés MC, Moscoloni N. Factorial and multiple correspondence analysis of major burnout indicators in anesthesiologists. *Revista Argentina de Anestesiologia.* 2017;75(2):45-52.
31. Sánchez-Cruz J, Mugártegui-Sánchez S. [Burnout syndrome among family physicians]. *Revista medica del Instituto Mexicano del Seguro Social.* 2013;51(4):428-431.
32. Taycan O, Taycan SE, Celik C. Relationship of burnout with personality, alexithymia, and coping behaviors among physicians in a semiurban and rural area in Turkey. *Archives of environmental & occupational health.* 2014;69(3):159-166.
33. Beltrán A, Manuel C, Manuel P-M, et al. Síndrome de Burnout en médicos familiares del Instituto Mexicano del Seguro Social, Guadalajara, México. *Revista Cubana de Salud Pública.* 2004;31.
34. Aftab H, Akram MA, Fatima A, et al. Physician's burnout rate at king edward medical university: A cross sectional survey. *Pakistan Journal of Medical and Health Sciences.* 2016;10(4):1207-1209.
35. Al-Dubai SA, Ganasegeran K, Perianayagam W, Rampal KG. Emotional burnout, perceived sources of job stress, professional fulfillment, and engagement among medical residents in Malaysia. *TheScientificWorldJournal.* 2013;2013:137620.
36. Peltzer K, Mashego TA, Mabeba M. Occupational stress and burnout among South African medical practitioners. *Stress and Health.* 2003;19(5):275-280.
37. Grau A, Flichtentrei D, Suñer R, Prats M, Braga F. Influence of personal, professional and cross-national factors in burnout syndrome in hispanic americans and spanish health workers (2007). *Revista Espanola de Salud Publica.* 2009;83(2):215-230.
38. Kosan Z, Calikoglu EO, Guraksin A. Levels of burnout and their associated factors among physicians working in Northeast Anatolia. In: *Niger J Clin Pract.* Vol 21. India2018:875-881.

© 2020 Sabitova A et al. *JAMA Network Open.*

39. Rajan S, Engelbrecht A. A cross-sectional survey of burnout amongst doctors in a cohort of public sector emergency centres in Gauteng, South Africa. *African Journal of Emergency Medicine.* 2018;8(3):95-99.
40. Salem R, Akel R, Fakhri G, Tfayli A. Burnout among Lebanese Oncologists: Prevalence and Risk Factors. *Asian Pacific journal of cancer prevention : APJCP.* 2018;19(8):2135-2139.
41. Zheng H, Shao H, Zhou Y. Burnout Among Chinese Adult Reconstructive Surgeons: Incidence, Risk Factors, and Relationship With Intraoperative Irritability. In: *J Arthroplasty.* Vol 33. United States: 2017 Elsevier Inc; 2018:1253-1257.
42. Huri M, Bagis N, Eren H, Umaroglu M, Orhan K. Association between burnout and depressive symptoms among Turkish dentists. *J Dental Sci.* 2016;11(4):353-359.
43. Esquivel-Molina CG, Buendia-Cano F, Martinez-Garcia O, Martinez-Mendoza JA, Martinez-Ordaz VA, Velasco-Rodriguez VM. [Burnout syndrome in medical staff affiliated to a tertiary care hospital]. *Revista medica del Instituto Mexicano del Seguro Social.* 2007;45(5):427-436.
44. Paiva CE, Martins BP, Paiva BSR. Doctor, are you healthy? A cross-sectional investigation of oncologist burnout, depression, and anxiety and an investigation of their associated factors. *BMC Cancer.* 2018;18(1):1044.
45. Xiao Y, Wang J, Chen S, et al. Psychological distress, burnout level and job satisfaction in emergency medicine: A cross-sectional study of physicians in China. *Emergency medicine Australasia : EMA.* 2014;26(6):538-542.
46. Zhou XY, Pu JC, Zhong XN, et al. Burnout, psychological morbidity, job stress, and job satisfaction in Chinese neurologists. *Neurology.* 2017;88(18):1727-1735.
47. Pu J, Zhou X, Zhu D, et al. Gender differences in psychological morbidity, burnout, job stress and job satisfaction among Chinese neurologists: a national cross-sectional study. *Psychology, health & medicine.* 2017;22(6):680-692.
48. Ozyurt A, Hayran O, Sur H. Predictors of burnout and job satisfaction among Turkish physicians. *QJM : monthly journal of the Association of Physicians.* 2006;99(3):161-169.
49. Zhang Y, Feng X. The relationship between job satisfaction, burnout, and turnover intention among physicians from urban state-owned medical institutions in Hubei, China: a cross-sectional study. *BMC health services research.* 2011;11:235.
50. Cetina-Tabares RE, Chan-Canul AG, Sandoval-Jurado L. Correlation between the level of work satisfaction and professional burnout in family physicians. *Revista medica del Instituto Mexicano del Seguro Social.* 2006;44(6):535-540.
51. Sararaks S, Jamaluddin R. Job satisfaction of doctors in Negeri Sembilan. *The Medical journal of Malaysia.* 1997;52(3):257-263.
52. Córdoba RN, Cano JF, Alzate M, Olarte AF, Salazar I, Cendales R. The latin American psychiatrist: Profile and degree of satisfaction with the specialty. *Actas Espanolas de Psiquiatria.* 2009;37(1):9-16.
53. Wu D, Wang Y, Lam KF, Hesketh T. Health system reforms, violence against doctors and job satisfaction in the medical profession: a cross-sectional survey in Zhejiang Province, Eastern China. *BMJ open.* 2014;4(12):10.
54. Valderrama-Martinez JA, Davalos-Diaz G. Job satisfaction among primary care physicians at the IMSS. *Revista de investigacion clinica; organo del Hospital de Enfermedades de la Nutricion.* 2009;61(2):119-126.
55. Pau A, Sabri BA. Relationship between emotional intelligence and job satisfaction in newly qualified Malaysian dentists. *Asia Pac J Public Health.* 2015;27(2):NP1733-1741.
56. Lu Y, Hu XM, Huang XL, et al. The relationship between job satisfaction, work stress, work-family conflict, and turnover intention among physicians in Guangdong, China: a cross-sectional study. *BMJ open.* 2017;7(5):e014894.
57. Ali Jadoo SA, Aljunid SM, Dastan I, et al. Job satisfaction and turnover intention among Iraqi doctors--a descriptive cross-sectional multicentre study. *Human resources for health.* 2015;13:21.

© 2020 Sabitova A et al. *JAMA Network Open.*

58. García-Peña C, Reyes-Frausto S, Reyes-Lagunes I, Muñoz-Hernández O. Family physician job satisfaction in different medical care organization models. *Family practice.* 2000;17(4):309-313.
59. O'Leary P, Wharton N, Quinlan T. Job satisfaction of physicians in Russia. *International Journal of Health Care Quality Assurance.* 2009;22(3):221-231.
60. Omolase C, Seidu M, Omolase B, Agborubere D. Job satisfaction amongst Nigerian ophthalmologists: an exploratory study. *The Libyan journal of medicine.* 2010;5(1):4629.
61. Ribeiro RB, Assuncao AA, de Araujo TM. Factors associated with job satisfaction among public-sector physicians in Belo Horizonte, Brazil. *International journal of health services : planning, administration, evaluation.* 2014;44(4):787-804.
62. Smith HE. Doctors and society: a Northern Thailand study. *Social science & medicine (1982).* 1982;16(5):515-526.
63. Yao Y, Wang W, Wang F, Yao W. General self-efficacy and the effect of hospital workplace violence on doctors' stress and job satisfaction in China. 2014.
64. Zhang MQ, Zhu CJ, Dowling PJ, Bartram T. Exploring the effects of high-performance work systems (HPWS) on the work-related well-being of Chinese hospital employees. *Int J Hum Resour Manag.* 2013;24(16):3196-3212.
65. Ashraf H, Shah N, Anwer F, Akhtar H, Abro MA, Khan A. Professional satisfaction of family physicians in Pakistan--results of a cross-sectional postal survey. *JPMA The Journal of the Pakistan Medical Association.* 2014;64(4):442-446.
66. Bhattacherjee S, Ray K, Roy JK, Mukherjee A, Roy H, Datta S. Job Satisfaction among Doctors of a Government Medical College and Hospital of Eastern India. *Nepal J Epidemiol.* 2016;6(3):595-602.
67. Cui XX, Dunning DG, An N. Satisfaction among early and mid-career dentists in a metropolitan dental hospital in China. *J Healthc Leadersh.* 2017;9:35-45.
68. Ghazaili SNAS, Daud N. Malaysian family physicians: Are they satisfied with their job? *Malaysian Journal of Public Health Medicine.* 2016;16(2):24-29.
69. Jabbari H, Pezeshki MZ, Naghavi-Behzad M, Asghari M, Bakhshian F. Relationship between job satisfaction and performance of primary care physicians after the family physician reform of east Azerbaijan province in Northwest Iran. *Indian journal of public health.* 2014;58(4):256-260.
70. Kaipa S, Pydi SK, Krishna Kumar RV, Srinivasulu G, Darsi VR, Sode M. Career satisfaction among dental practitioners in Srikakulam, India. *Journal of International Society of Preventive & Community Dentistry.* 2015;5(1):40-46.
71. Khan TH. Job satisfaction in Pakistani anesthesiologists. *Anaesthesia, Pain and Intensive Care.* 2011;15(2):93-101.
72. Khuwaja AK, Qureshi R, Andrades M, Fatmi Z, Khuwaja NK. Comparison of job satisfaction and stress among male and female doctors in teaching hospitals of Karachi. *Journal of Ayub Medical College, Abbottabad : JAMC.* 2004;16(1):23-27.
73. Kisa S, Kisa A. Job dissatisfaction among public hospital physicians is a universal problem: evidence from Turkey. *The health care manager.* 2006;25(2):122-129.
74. Luboga S, Hagopian A, Ndiku J, Bancroft E, McQuide P. Satisfaction, motivation, and intent to stay among Ugandan physicians: a survey from 18 national hospitals. *The International journal of health planning and management.* 2011;26(1):2-17.
75. Çakır M, İlhan MN. Occupational physicians and job satisfaction in Ankara *ANKARA'DA İŞYERİ HEKİMLERİ VE İŞ DOYUMU.* 2018;14(1):14-22.
76. Fierro-Arias L, Simon-Diaz P, Ponce-Olivera RM, Arenas-Guzman R. Dermatologists happiness and satisfaction. In: *Gac Med Mex.* Vol 154. Mexico: : 2018 SecretarIa de Salud; 2018:26-35.
77. Barikani A, Javadi M, Mohammad A, Firooze B, Shahnazi M. Satisfaction and motivation of general physicians toward their career. *Global journal of health science.* 2013;5(1):166-173.

78. Chew BH, Ramli AS, Omar M, Ismail IZ. A preliminary study of job satisfaction and motivation among the Malaysian primary healthcare professionals. *Malaysian Family Physician.* 2013;8(2):15-25.
79. de Oliveira Vasconcelos Filho P, de Souza MR, Elias PE, D'Avila Viana AL. Physicians' job satisfaction and motivation in a public academic hospital. *Human resources for health.* 2016;14(1):75.
80. Mendes AC, de Araújo Júnior JL, Furtado BM, Duarte PO, da Silva AL, Miranda GM. [Conditions and motivations for the work of nurses and physicians in high complexity emergency services]. *Revista brasileira de enfermagem.* 2013;66(2):161-166.

© 2020 Sabitova A et al. *JAMA Network Open.*
